# Supplementary material for: Quality of life in elderly ICU survivors before the COVID-19 pandemic: a systematic review and meta-analysis of cohort studies
Source: BMJ Open. 2021 Oct 11;11(10):e045086. doi: 10.1136/bmjopen-2020-045086 (PMC8506050; doi:10.1136/bmjopen-2020-045086)
Supplement: Supplementary data [file bmjopen-2020-045086supp001.pdf]

ICU Systematic Review: Appendix

1. STUDY CHARACTERISTICS

1.1 Meta-Analysis

First Author	Year	Country	Study Design	Journal	Setting	Min Age	Avg. Age (SD)	% Male	Mortality	ICU LoS (SD)	HLoS (SD)	Severity	Raw Measure	Follow up	Comparison	Study Quality	Participant No.	Control No.	Effect Size	Variance
Abelha[39]	2007	Portugal	Cohort (unspecified)	BMC Anaesthesiology	Surgical ICU	65+		61.00%	28.00%				SF-36 *	6 months	ICU survivors younger than 65 years old	M	112	114	-.07	.02
Ali [38]	2018	Australia	Prospective Cohort	Journal of Critical Care	Medical-Surgical ICU	65+	73 (5)	80.00% [a]		4.64 (2.32)	16.29 (9.28)	.24	EQ-5D	12 months	Age-matched South Australian controls	H	32	572	.03	.03
Andersen [37]	2015	Norway	Retrospective Cohort	Annals of Intensive Care	General Hospital ICU	80+	87.4 (4)	69.00%	81.52%	1.9 (NR)		.27	EQ-5D	40.8 months	Age and sex-matched Norwegian population	M	53	170	-.18	.02
De Rooij [35]	2008	Netherlands	Retrospective Cohort	Journal of the American Geriatric Society	Medical-Surgical ICU	80+	81.7 (2.4)	51.00%	61.52%	1.29 (1.13)		.21	EQ-5D	44.4 months	Age-matched British population	M	187	142	-.24	.01
Eddleston [34]	2000	UK	Prospective Cohort	Critical Care Medicine	General Hospital ICU	65+		52.45%[a]					SF-36 *	3 months	ICU survivors younger than 65 years old	M	39	97	-.21	.04
Ferrao [33]	2015	Portugal	Retrospective Cohort	Critical Care	Medical-Surgical ICU	66+ [b]		26.00%					EQ-5D	27.6 months	ICU survivors younger than 65 years old	M	290	652	-.37	.01
Grace [31]	2007	Australia/NZ	Retrospective Cohort	Critical Care and Resuscitation	Mixed ICUs	60+		NR	60.00%			.28	EQ-5D	28 months	Retrospective patient ratings for one week before ICU	L	99	99	-.36	.02
Hofhuis [30]	2011	Netherlands	Prospective Cohort	Chest	Medical-Surgical ICU	80+ [b]	83 (3.06)	46.90%	40.83%	5.35 (2.29)	25.48 (16.04)	.25	SF-36 *	6 months	Age-matched Dutch population	M	49	49 [c]	.26	.04
															Retrospective proxy ratings for four weeks before ICU		49	49	.01	.04
Honselmann	2015	Germany	Retrospective Cohort	Journal of Critical Care (part unpublished)	Mixed ICU (unpublished)	65+	75.84	53.00%	43.00%	2.58 (NR)			EQ-5D	12 months	ICU survivors younger than 65 years old	N/A (unpublished)	352	249	.90	.00
							75.16	54.00%	43.00%	2.34			EQ-5D	12 months	Age-matched German controls	N/A (unpublished)	291	828	.41	.00

ICU Systematic Review: Appendix

Jeitziner [29]	2015	Switzerland	Retrospective Cohort	Journal of Clinical Nursing	Medical-Surgical ICU	65+	68.72 (5.39)	73.00%		4.57 (5.81)		.29	SF-36 *	12 months	Age matched Swiss controls;	M	124	145	-.59	.02
															Retrospective patient ratings for one week before ICU		124	135	-.08	.01
Kaarola [28]	2006	Finland	Cross-Sectional	Critical Care Medicine	Medical-Surgical ICU	65+		75.00%	57.00%				EQ-5D	47 months	ICU survivors younger than 65 years old	M	299	800	-.67	.00
Levinson [26]	2016	Australia	Prospective Cohort	Internal Medicine Journal	Private ICU	80+	84.59 (NR)	58.00%[a]	21.45%	1.28 (NR)	12.91 (NR)		SF-36 *	24 months	Age and sex-matched Australian population	H	322	907	.04	.00
Merlani [25]	2007	Switzerland	Retrospective Cohort	Acta Anaesthesiologica Scandinavica	Surgical ICU	70+	78 (5)	52.00%	63.00%	3.00 (13.72)	22.50 (93.88)	.26	SF-36 *	24 months	Age-matched Swiss population	M	36	87	-.23	.04
Oeyen [24]	2007	Netherlands	Prospective Cohort	Minerva Medica	Medical-Surgical ICU	80+	83 (3)	60.00%[a]	49.60%	3.35 (2.26)	26.93 (27.11)	.26	EQ-5D	12 months	Retrospective patient or proxy ratings for one week before ICU	M	63	63	-.30	.03
Sacanella [23]	2011	Spain	Prospective Cohort	Critical Care	Medical ICU	65+	73.4 (5.5)	57.00%	48.70%	9.4 (10.20)		.27	EQ-5D	12 months	Retrospective patient or proxy ratings before feeling ill and requiring ICU	M	112	112	-.49	.02
Schroder [22]	2011	Denmark	Cohort (unspecified)	Danish Medical Bulletin	Mixed ICUs	75+		56.00%	53.85%				SF-36 *	12 months	Age-matched Danish population	L	36	229	-.03	.03
Sznajer [21]	2001	France	Prospective Cohort	Intensive Care Medicine	Mixed ICUs	65+ [b]		55.90%[a]					EQ-5D	6 months	ICU survivors younger than 65 years old	M	65	53	-.16	.03
Villa [19]	2016	Spain	Prospective Cohort	Journal of the American Geriatric Society	Medical-Surgical ICU	75+	80.8 (3.3)	50.00%	43.18%			.23	SF-36 *	12 months	Spanish population aged 75+	M	54	1363 [d]	-.15	.02

Table A1 Full study characteristics for all effect sizes included in the meta-analysis

[a] Reported for study level only
[b] Combined elderly groups
[c] Assumed N based on matched sample
[d] Retrieved from López-García, E., Banegas, J. R., Graciani, A. P. R., Gutiérrez-Fisac, J. L., Alonso, J., & Rodríguez-Artalejo, F. (2003). Population-based reference values for the Spanish version of the SF-36 Health Survey in the elderly. Medicina clinica, 120(15), 568-573; a follow-up to the previous study, which was unavailable
[e] Unless specified, we do not report data where it is not representative of at least 66.67% of the included sample
[f] Abbreviations: Avg. Age (average age); ICU LoS (average length of stay in intensive care; days); HLoS (average length of stay in hospital; days); SD (standard deviation; sometimes estimated- see methods)

ICU Systematic Review: Appendix

NOTE: If studies are reported in duplicate, for the second row, assume blank cells are the same value as the row above, unless otherwise specified.

1.2 Qualitative Only Studies

First Author	Year	Country	Study Design	Journal	Setting	Min Age	Participant No.	Avg. Age (SD)	% Male	ICU LoS (SD)	HLoS (SD)	Severity	Ineligible Measure	Follow up	Comparison
Cuthbertson	2010	Scotland	Prospective Cohort	Critical Care	Medical-Surgical ICU	65+	116						SF-36 (MCS/PCS only)	12 months (paper reports up to 60 months)	ICU survivors younger than 65 years old AND retrospective ratings for a period before ICU
Garrouste-Orgeas	2006	France	Prospective Cohort	Intensive Care Medicine	Medical ICU	80+	28	84 (3.92)		12.6 (15.5)		.28	Nottingham Health Profile (NHP)	12 months	Age and sex-matched French population controls
Kleinpell	2002	USA	Retrospective Cohort	Research in Nursing and Health	Mixed ICUs	66+	128		42.00%	4.2 (6.17)	10.28 (9.63)	.18	Quality of Life Index (QLI)	4-6 months	ICU survivors aged between 45 and 64 years old
Tabah	2010	France	Prospective Cohort	Critical Care	Medical-Surgical ICU	80+	23	84 (3)	73.90%	5.72 (4.74)	18.08 (15.01)	.23	WHO-QOL-BREF	16 months	Age and sex-matched French population controls

Table A2 Full study characteristics of all records that were only included in the qualitative synthesis
[a] Reported for study level only
[b] Abbreviations: Avg. Age (average age); ICU LoS (average length of stay in intensive care; days); HLoS (average length of stay in hospital; days), SD (standard deviation; sometimes estimated- see methods)
[c] Unless specified, we do not report data where it is not representative of at least 66.67% of the included sample.

ICU Systematic Review: Appendix

2. SENSITIVITY ANALYSES FOR INFLUENTIAL CASES

2.1 Overview of Outliers: Meta-Analysis

Comparison	k	First Author	Cook's Distance (Critical d)	Leave out Effect Size	Leave out P value	I^2 Change	Effect Size Change
Community	11	Pavoni	.97 (.36)	-1.97	.27	-12%	+1.74
Community	10	Honselmann	.56 (.40)	-.13	.10	-21%	+.08

Table A3 A summary of cases that fit our criteria as potentially influential
[a] Excluded cases are highlighted in red

First Author	Year	Country	Study Design	Journal	Setting	Min Age	Participant No.	Avg. Age (SD)	% Male	ICU LoS (SD)	HLoS (SD)	Severity	Mortality	Follow up	Comparison
Pavoni	2012	Italy	Prospective Cohort	Archives of Gerontology and Geriatrics	Mixed ICUs	80+	143	86.51 [a] (1.81)	26.74% [a]	5.27 [a] (5.80)	14.20 [a] (8.96)	.20 [a]	50% [a]	12 months	Age-matched Italian retirement community population

Table A4 Study characteristics of the lone study excluded as an outlier
[a] Reported for study level only
[b] Abbreviations: Avg. Age (average age); ICU LoS (average length of stay in intensive care; days); HLoS (average length of stay in hospital; days), SD (standard deviation; sometimes estimated- see methods)

ICU Systematic Review: Appendix

3. QUALITATIVE SYNTHESIS

3.1 Qualitative analysis procedure

Scale	Mental Health Subscale(s)	Physcial Health Subscale(s)	Additional Notes
EQ-5D	Anxiety/Depression	Mobility, Self-Care, Usual Activities, Pain/Discomfort	Raw scores scaled between 1-3
SF-36	Social Functioning, Role Emotional, Mental Health, Vitality	Physical Functioning, Bodily Pain, General Health, Role Physical	
NHP	Sleep, Emotional Reaction, Social Isolation	Pain, Energy, Physical Mobility	Reverse scoring
WHO-QOL-BREF	Psychological Health, Social Relationships	Overall perception of Health, Physical Health, Environment	
QLI	Socio-economic, Family, Psychological/Spiritual	Health and Functioning	Raw scores scaled between 0-30

Table A5 Subscales used to estimate mental and physical health QoL within the qualitative synthesis

ICU Systematic Review: Appendix

4. SENSITIVITY ANALYSES FOR OBSERVED EFFECTS

4.1 Forest Plots

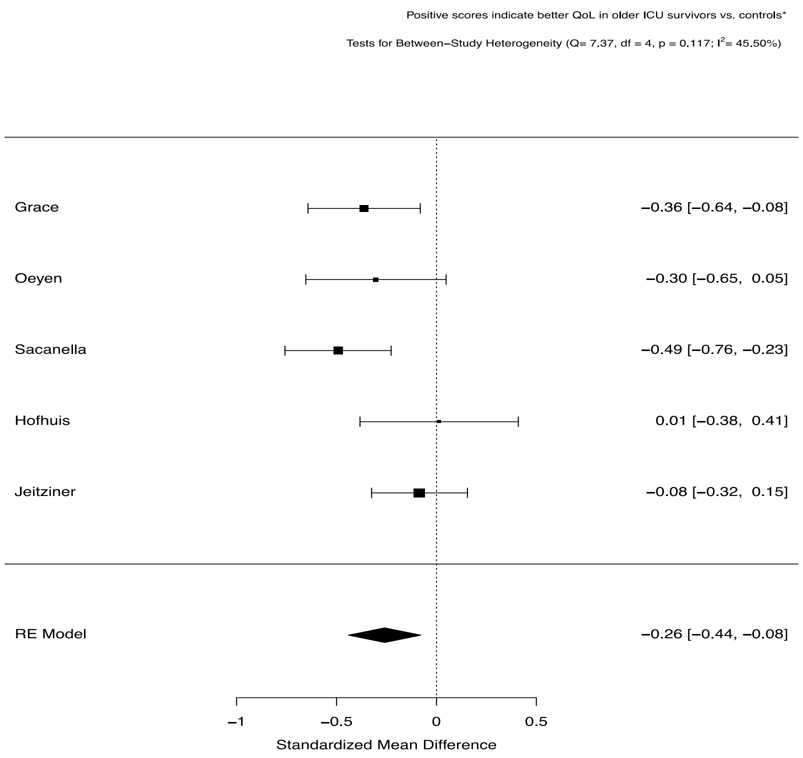

Fig. A1 Forest plot of differences in EQ-5D composite scores in elderly survivors, comparing pre-ICU and post-ICU scores

ICU Systematic Review: Appendix

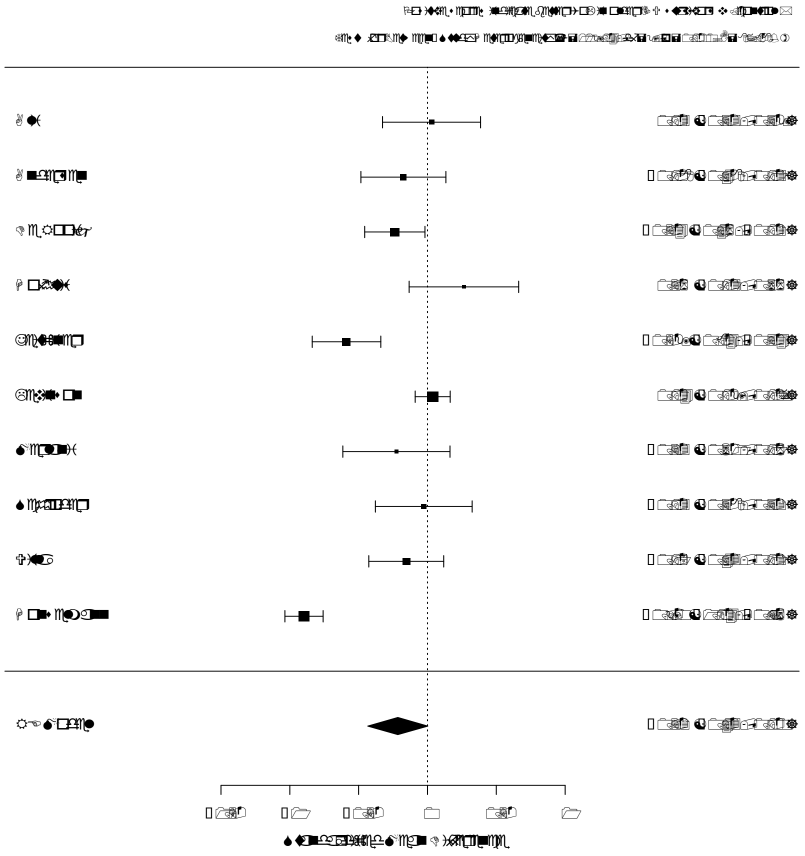

Fig. A2 Forest plot of differences in EQ-5D composite scores, comparing elderly ICU survivors at follow-up and age-matched community controls

ICU Systematic Review: Appendix

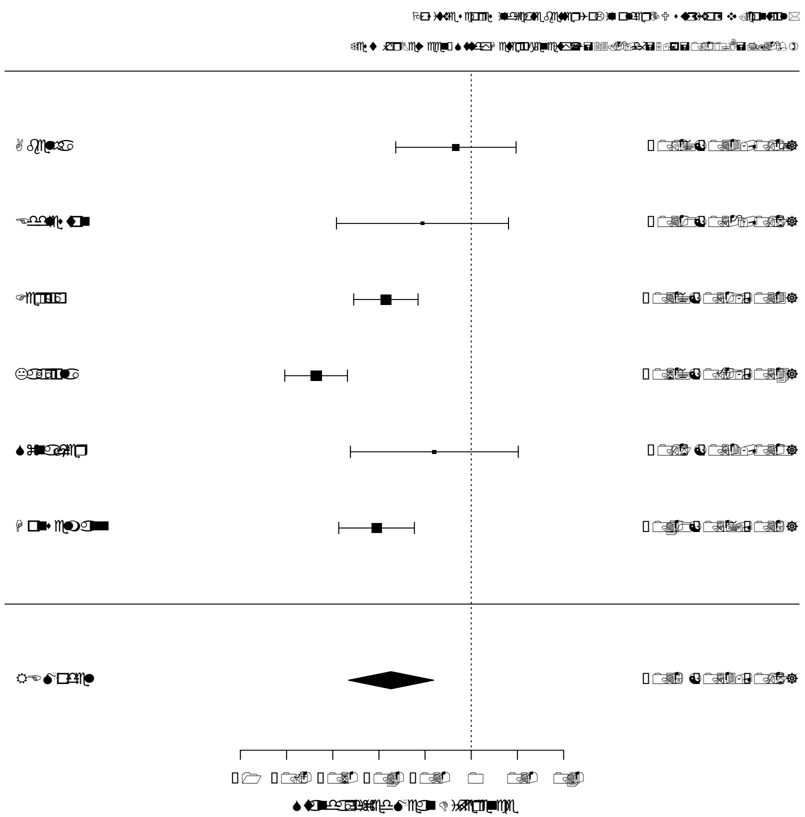

Fig. A3 Forest plot of differences in EQ-5D composite scores at follow-up, comparing elderly ICU survivors (aged 65+) and younger ICU survivors (aged under 65), both at follow-up

Appendix: Disparity or Discrimination?
A systematic review of socio-demographic associations of insight

4.2 Funnel Plots

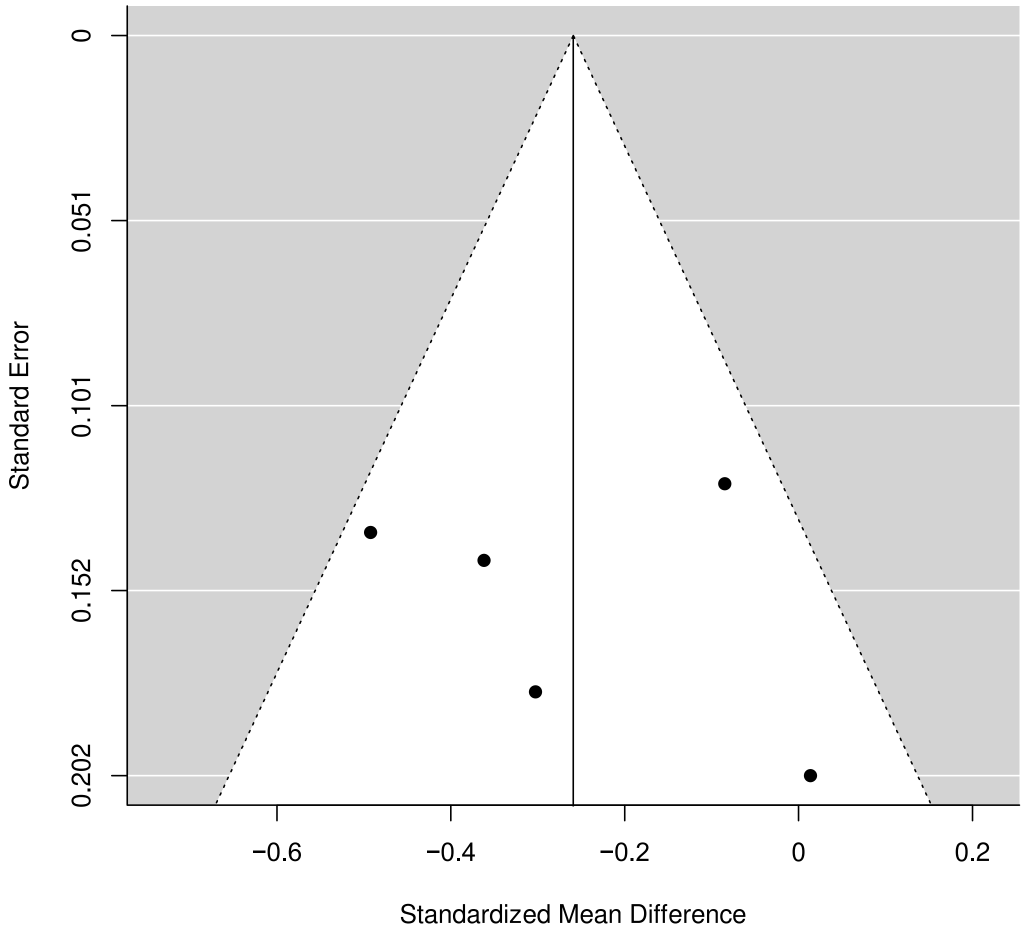

Fig. A4 Funnel plot of studies that investigated differences in EQ-5D composite scores in elderly survivors, comparing pre-ICU and post-ICU scores

Appendix: Disparity or Discrimination?
A systematic review of socio-demographic associations of insight

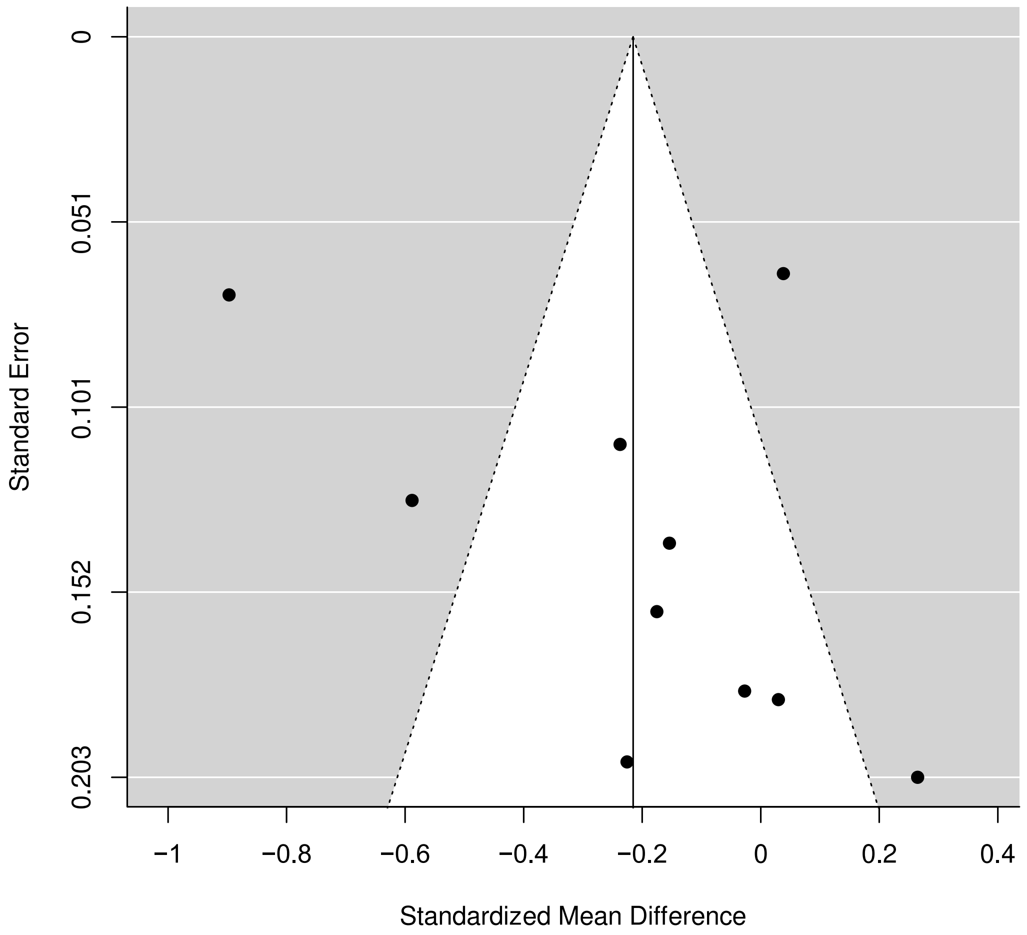

Fig. A5 Funnel plot of studies that compared EQ-5D scores in elderly ICU survivors at follow-up and age-matched community controls

Appendix: Disparity or Discrimination?
A systematic review of socio-demographic associations of insight

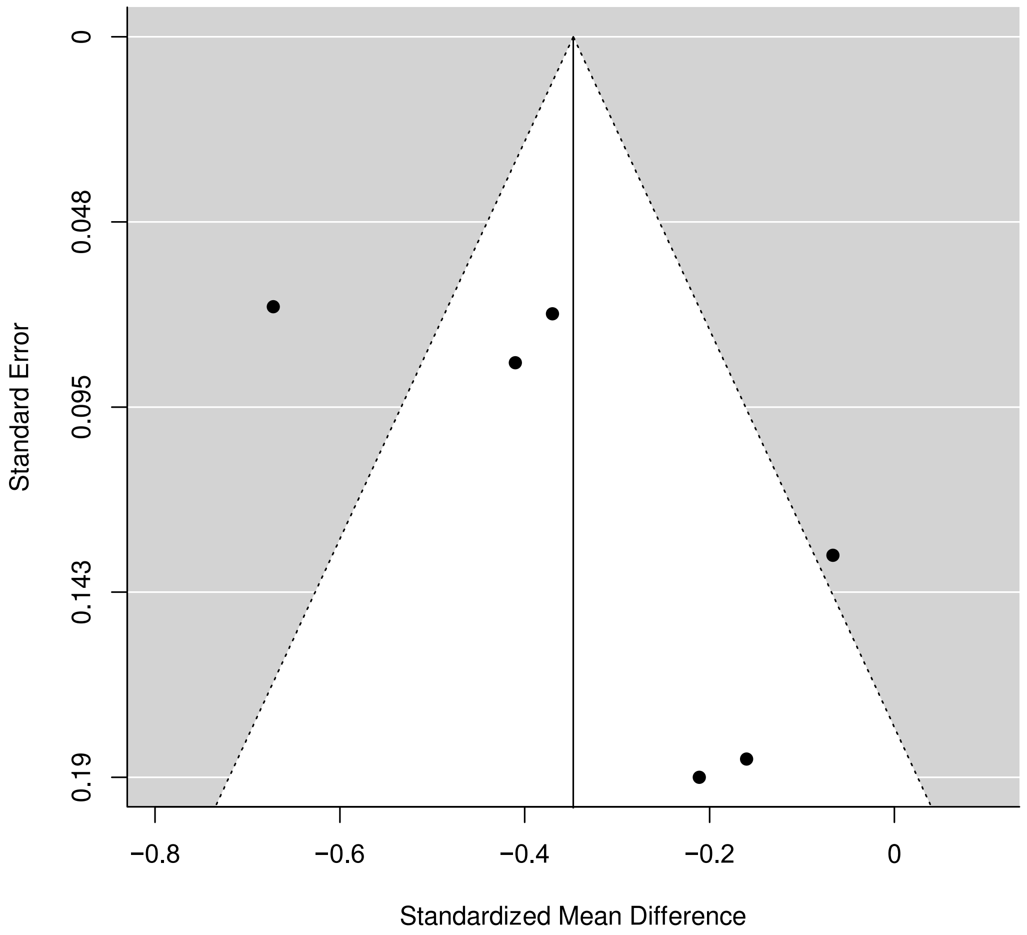

Fig. A6 Funnel plot of studies that compared EQ-5D scores in elderly ICU survivors (aged 65+) and younger ICU survivors (aged under 65), both at follow-up

Appendix: Disparity or Discrimination?
A systematic review of socio-demographic associations of insight

4.3 Cook's Distance Plots

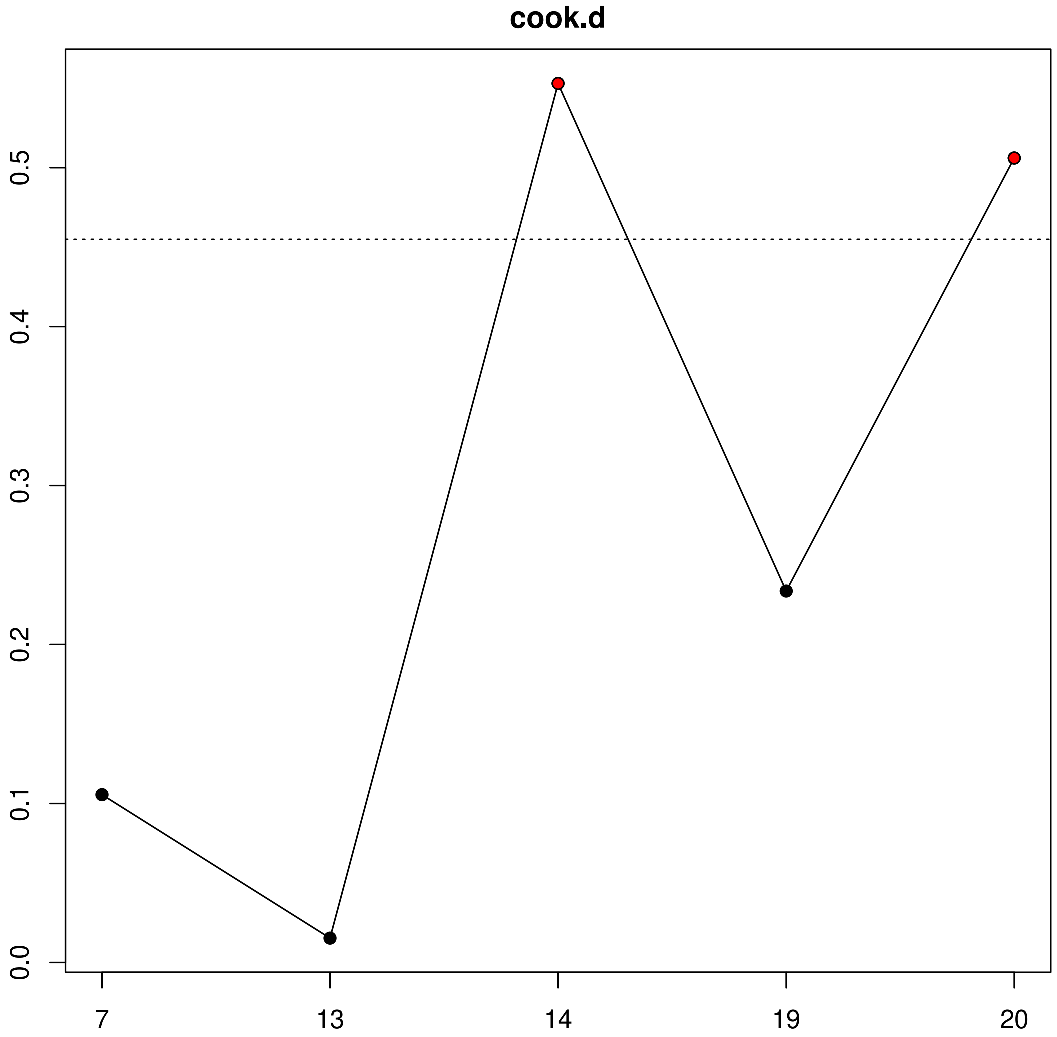

Fig. A7 Cook's distance plot of studies that investigated differences in EQ-5D composite scores in elderly survivors, comparing pre-ICU and post-ICU scores

Appendix: Disparity or Discrimination?
A systematic review of socio-demographic associations of insight

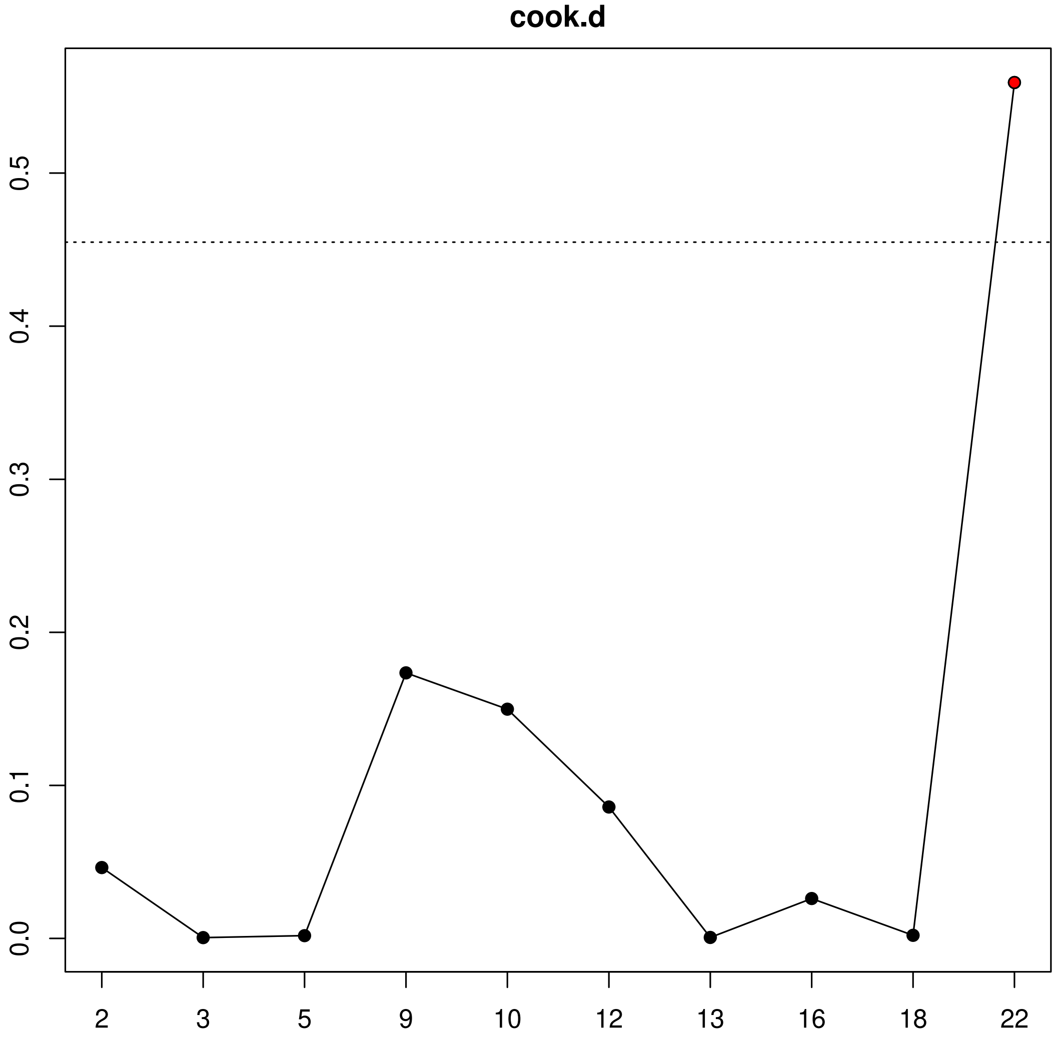

Fig. A8 Cook's distance plot of studies that compared EQ-5D scores in elderly ICU survivors at follow-up and age-matched community controls

Appendix: Disparity or Discrimination?
A systematic review of socio-demographic associations of insight

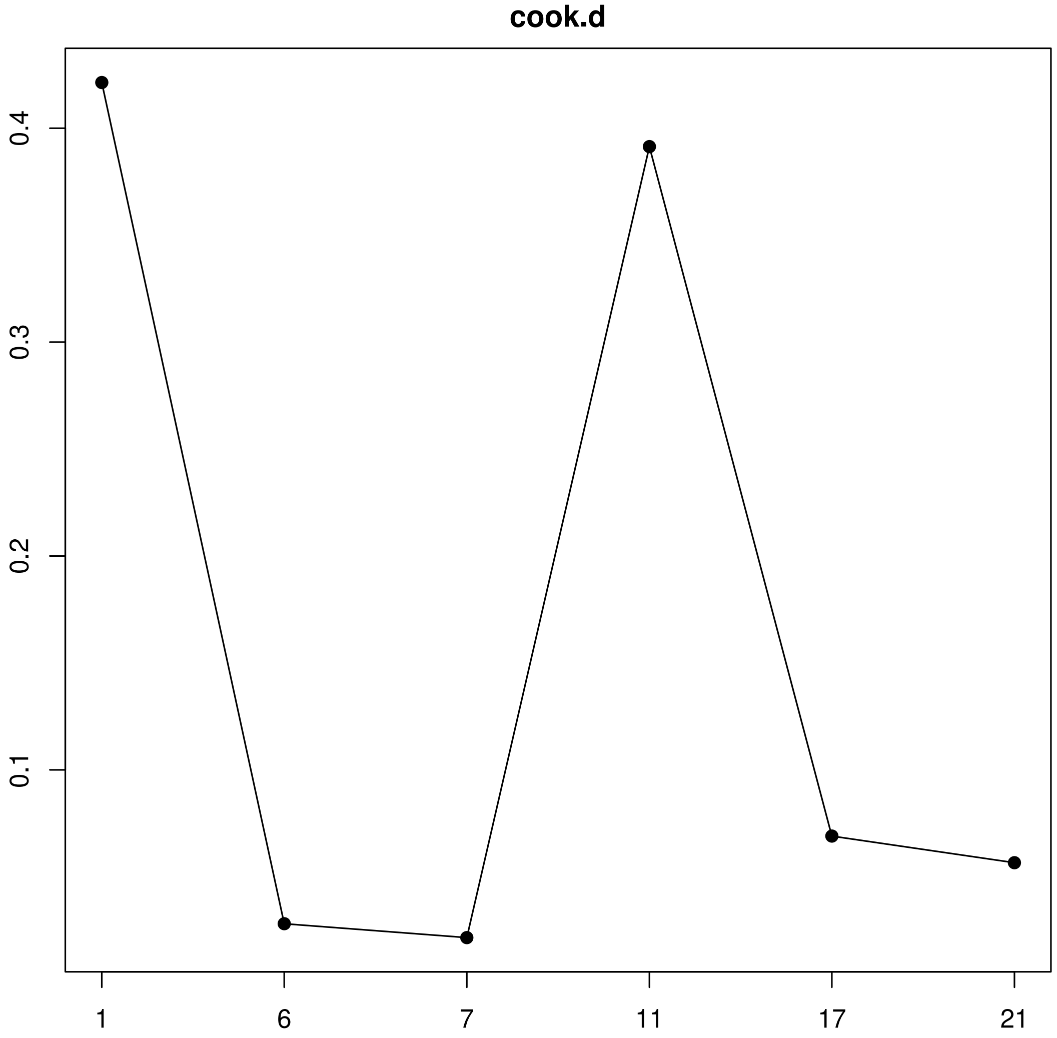

Fig. A9 Cook's distance plot of studies that compared EQ-5D scores in elderly ICU survivors (aged 65+) and younger ICU survivors (aged under 65), both at follow-up

Appendix: Disparity or Discrimination?
A systematic review of socio-demographic associations of insight

5. REVIEW PROTOCOL

5.1 ICU Review Protocol

**Included**	**Excluded**
**Design**	
Case note analyses (longitudinal)<br>Case control<br>Retrospective cohort<br>Prospective cohort<br>Unpublished dissertations of the above	Qualitative only studies<br>Systematic review or meta-analysis (categorise in separate folder)<br>Narrative review<br>Non-English language (if translation can't be found)<br>Commentaries<br>Case studies<br>Small N samples (<20 eligible participants)<br>Conference abstracts<br>Brief reports<br>Books
**Population**	
Patients aged 60+ who have undergone ICU<br>Medical, Surgical or Mixed ICU settings	<20 eligible patients aged 60+<br>Veteran, trauma or emergency care setting<br>Non-OECD country<br>Non-human participants<br>Palliative care<br>Non-ICU patients
**Focus**	
Patients aged 60+ who have undergone ICU	Neurological ICU patients only<br>Cardiosurgical ICU patients only
Follow up of at least 3 months	No follow up/Follow up less than three months
At least one of the following comparison groups:<br>• Age-matched community controls<br>• Scores taken before ICU<br>• Younger ICU patients	No comparison group
QoL at follow up measured by patients (carers may help but cannot do assessment on their own)	QoL at follow up all measured by proxy (ie. doctors or carers)
**Data/Outcomes**	
Validated QoL measure (EQ-5D, SF-36, NHP, WHOQOLBREF, QLI or variants of these)	Non-validated QoL measure only (eg. a simple question of whether QoL improved)
QoL summary score reported in paper for both groups, or:<br>• Subscores can be used to calculate summary scores<br>• Study references data for age-matched control that is fully reported elsewhere	No eligible data on QoL (or insufficient data to calculate summary scores)<br>QoL not reported for both groups (regression analyses do not count)

Ariyo K, *et al. BMJ Open* 2021; 11:e045086. doi: 10.1136/bmjopen-2020-045086

Appendix: Disparity or Discrimination?
A systematic review of socio-demographic associations of insight

6. REVIEW SEARCH TERMS

6.1 MEDLINE

(("intensive care"[title/abstract] OR "critical care"[title/abstract] OR "critical illness"[title/abstract] OR "Respiratory Distress Syndrome"[title/abstract] OR "Sepsis"[title/abstract] OR intensive care[MeSH Terms] OR critical care[MeSH Terms] OR "critical illness"[MeSH Terms] OR "Sepsis"[MeSH Terms]))

AND (("elderly"[title/abstract] OR "older adult*"[title/abstract] OR “geriatr*”[title/abstract] OR “dement*”[title/abstract] OR “Alzheimer*”[title/abstract] OR “parkinson’s disease”[title/abstract] OR elderly [MeSH Terms] OR older adult*[MeSH Terms] OR geriatr*[MeSH Terms] OR dement*[MeSH Terms] OR septugenaria*[All Fields] OR octogenaria*[All Fields] OR nonagenaria*[All Fields] OR "over 5*"[title/abstract] OR "over 6*"[title/abstract] OR "over 7*"[title/abstract] OR "over 8*"[title/abstract] OR "over 9*"[title/abstract] OR "over 5*"[title/abstract] OR "over 6*"[title/abstract] OR "over 7*"[title/abstract] OR "over 8*"[title/abstract] OR "over 9*"[title/abstract]))

AND (("quality of life"[title/abstract] OR "EuroQol*"[All Fields] OR "Nottingham Health Profile"[All Fields] OR "NHP*"[All Fields] OR "SF-36"[All Fields] OR "RAND-36*"[All Fields]))

Filters: English Language, Humans, 01/01/2000 to 23/04/2020

6.2 Cochrane Database for Systematic Reviews & Cochrane Controlled Register of Trials (CENTRAL)

#1 ("intensive care" OR "critical care" OR "critical illness" OR "Respiratory Distress Syndrome" OR "Sepsis"):ti,ab,kw
#2 ("elderly" OR "older adult*" OR “geriatr*” OR “dement*” OR “Alzheimer*” OR “parkinson’s disease”):ti,ab,kw
#3 (critical care OR critical illness OR Sepsis)
#4 (Aged OR geriatrics OR dementia)
#5 ("quality of life")
#6 ("EuroQol" OR "Nottingham Health Profile" OR "NHP" OR "SF-36" OR "RAND-36")
#7 MeSH descriptor: [Aged]
#8 MeSH descriptor: [Geriatrics]
#9 MeSH descriptor: [Dementia]
#10 MeSH descriptor: [Critical Care]
#11 MeSH descriptor: [Critical Illness]
#12 MeSH descriptor: [Sepsis]
#13 #1 OR #3 OR #10 OR #11 OR #12
#14 #2 OR #4 OR #7 OR #8 OR #9
#15 #5 AND #6
#16 #13 AND #14 AND #15= 124 (78 reviews, 36 trials).

6.3 Web of Science

Indexes = SCI-EXPANDED, SSCI, CPCI-S, CPCI-SHH, ESCI. **LANGUAGE** = English, **DOCUMENT TYPES** = (Article OR Abstract of Published Item), Timespan = All years (2000-2020)

#1 ALL=("intensive care" OR "critical care" OR "critical illness" OR "Respiratory Distress Syndrome" OR "Sepsis" OR “ICU”)
#2 ALL=("elderly" OR "older adult*" OR “geriatr*” OR “dement*” OR “Alzheimer*” OR “parkinson’s disease”)
#3 ALL= ("quality of life" OR "EuroQol" OR "Nottingham Health Profile" OR "NHP" OR "SF-36" OR "RAND-36")
#4 #1 AND #2 AND #3
#5 #4 AND **LANGUAGE**: (English) AND **DOCUMENT TYPES**: (Article OR Abstract of Published Item) AND **Timespan**= 2000-2020

Appendix: Disparity or Discrimination?
A systematic review of socio-demographic associations of insight

6.4 EMBASE (& EMBASE Classic)

Dates: 2000-2020, Limits: Human participants only, English language, Articles only

#1 All Field: "intensive care" or "critical care" or "critical illness" or "Respiratory Distress Syndrome" or Sepsis or "ICU"
#2 Text Word: elderly or "older adult*" or "geriatr*" or "dement*" or "Alzheimer*" or "parkinson*"
#3 All Field: "quality of life" or EuroQol or Nottingham Health Profile or NHP or SF-36 OR RAND-36

6.5 CINAHL

Limits: English language only, Human participants, All adult, Peer-reviewed, Jan 2000 – April 2020

#1 TX: "intensive care" or "critical care" or "critical illness" or "Respiratory Distress Syndrome" or Sepsis or "ICU"
#2: SU: "Intensive Care Units" or "Intensive Care Units or Neonatal" or "Critical Care Nursing" or "Respiratory Distress Syndrome" or Acute or "Neonatal Intensive Care Nursing" or "Critical Care or Critical Path" or "Canadian Association of Critical Care Nurses" or "British Association of Critical Care Nurses" or "ventilator patients"
#3: TX: elderly or "older adult*" or "geriatr*" or "dement*" or "Alzheimer*" or "parkinson*"
#4: SU: "Older Adult Care (Saba CCC)" or "Frail Elderly" or "elderly patients" or "ventilator patients"
#5: TX: "quality of life" or EuroQol or “Nottingham Health Profile” or NHP or SF-36 OR RAND-36
#6: (S1 OR S2) AND (S3 OR S4) AND S5

6.6 PsycINFO

Limits: Date filter (2000-2020), English language, Human participants, Peer Reviewed Journal

#1 All Fields: "intensive care" or "critical care" or "critical illness" or "Respiratory Distress Syndrome" or Sepsis or "ICU"
#2 Text Word: elderly or "older adult*" or "geriatr*" or "dement*" or "Alzheimer*" or "parkinson*"
#3 All Fields: "quality of life" or EuroQol or Nottingham Health Profile or NHP or SF-36 OR RAND-36
