# Supplementary material for: Quality of life in elderly ICU survivors before the COVID-19 pandemic: a systematic review and meta-analysis of cohort studies
Source: BMJ Open. 2021 Oct 11;11(10):e045086. doi: 10.1136/bmjopen-2020-045086 (PMC8506050; doi:10.1136/bmjopen-2020-045086)
Supplement: Author's manuscript [file bmjopen-2020-045086.draft_revisions.pdf]

BMJ Open

BMJ Open is committed to open peer review. As part of this commitment we make the peer review history of every article we publish publicly available.

When an article is published we post the peer reviewers' comments and the authors' responses online. We also post the versions of the paper that were used during peer review. These are the versions that the peer review comments apply to.

The versions of the paper that follow are the versions that were submitted during the peer review process. They are not the versions of record or the final published versions. They should not be cited or distributed as the published version of this manuscript.

BMJ Open is an open access journal and the full, final, typeset and author-corrected version of record of the manuscript is available on our site with no access controls, subscription charges or pay-per-view fees (http://bmjopen.bmj.com).

If you have any questions on BMJ Open's open peer review process please email info.bmjopen@bmj.com

Quality of Life in elderly ICU survivors: A Rapid Systematic Review and Meta-Analysis of Cohort Studies.

Journal:	*BMJ Open*
Manuscript ID	bmjopen-2020-045086
Article Type:	Original research
Date Submitted by the Author:	21-Sep-2020

Complete List of Authors:	Ariyo, Kevin; King's College London Department of Psychological Medicine<br>Canestrini, Sergio; King's College Hospital NHS Foundation Trust, Critical Care<br>David, Anthony; UCL, Institute of Mental Health<br>Ruck Keene, Alex; King's College London Department of Psychological Medicine; King's College London Dickson Poon School of Law<br>Owen, Gareth; King's College London Department of Psychological Medicine

Keywords:	COVID-19, INTENSIVE & CRITICAL CARE, STATISTICS & RESEARCH METHODS, ETHICS (see Medical Ethics), Rationing < HEALTH SERVICES ADMINISTRATION & MANAGEMENT

SCHOLARONE™
Manuscripts

I, the Submitting Author has the right to grant and does grant on behalf of all authors of the Work (as defined in the below author licence), an exclusive licence and/or a non-exclusive licence for contributions from authors who are: i) UK Crown employees; ii) where BMJ has agreed a CC-BY licence shall apply, and/or iii) in accordance with the terms applicable for US Federal Government officers or employees acting as part of their official duties; on a worldwide, perpetual, irrevocable, royalty-free basis to BMJ Publishing Group Ltd ("BMJ") its licensees and where the relevant Journal is co-owned by BMJ to the co-owners of the Journal, to publish the Work in this journal and any other BMJ products and to exploit all rights, as set out in our licence.

The Submitting Author accepts and understands that any supply made under these terms is made by BMJ to the Submitting Author unless you are acting as an employee on behalf of your employer or a postgraduate student of an affiliated institution which is paying any applicable article publishing charge ("APC") for Open Access articles. Where the Submitting Author wishes to make the Work available on an Open Access basis (and intends to pay the relevant APC), the terms of reuse of such Open Access shall be governed by a Creative Commons licence – details of these licences and which Creative Commons licence will apply to this Work are set out in our licence referred to above.

Other than as permitted in any relevant BMJ Author's Self Archiving Policies, I confirm this Work has not been accepted for publication elsewhere, is not being considered for publication elsewhere and does not duplicate material already published. I confirm all authors consent to publication of this Work and authorise the granting of this licence.

For peer review only

Quality of Life in elderly ICU survivors:
A Rapid Systematic Review and Meta-Analysis of Cohort Studies.

ABSTRACT

Kevin Ariyo [1], Sergio Canestrini [2,3], Anthony S. David [4], Alex Ruck Keene [1,5], Gareth S. Owen [1].

1. Department of Psychological Medicine, Institute of Psychiatry, Psychology and Neuroscience, King's College London, UK
2. Department of Critical Care, King's College Hospital, Denmark Hill, London, UK
3. Centre for Social Ethics and Policy, Department of Law, School of Social Science, University of Manchester, UK
4. UCL Institute of Mental Health, Division of Psychiatry, University College London, UK
5. Dickson Pool School of Law, King's College London, UK

BACKGROUND

The influence of age upon intensive care unit (ICU) decision-making is complex and it is unclear if it is based on expected subjective or objective patient outcomes. To address recent concerns over age-based ICU decision-making we explored patient-assessed quality of life (QoL) in ICU survivors.

METHODS

We searched online databases for cohort studies published between January 2000 to April 2020, of elderly patients admitted to ICUs. We extracted data on self-reported QoL (EQ-5D composite score), demographic and clinical variables. Using a random-effects meta-analysis, we then compared QoL scores at follow-up to scores either before admission, age-matched population controls or younger ICU survivors. We conducted sensitivity analyses to study heterogeneity and bias, and a qualitative synthesis of subscores.

FINDINGS

We identified 2536 studies and included 21 for qualitative synthesis and 18 for meta-analysis (N= 2090 elderly survivors). Elderly survivors' QoL was not significantly different between one month before ICU and follow-up, or between follow-up and age-matched community controls. Elderly survivors' QoL was significantly worse than younger ICU survivors, with a small-to-medium effect size (d= .33 [.10 to .55]). Mortality rates and length of follow up partly explained heterogeneity. Reductions in QoL seemed primarily due to physical health, rather than mental health items.

INTERPRETATION

The results suggest that the proportionality of age as a determinant of ICU resource allocation should be kept under close review and that subjective QoL outcomes should inform person-centred decision making in elderly ICU patients.

DECLARATIONS OF INTEREST

Alex Ruck Keene is an adviser on the Faculty of Intensive Care Medicine's Legal and Ethical Policy Unit. We report no other competing interests.

For peer review only - http://bmjopen.bmj.com/site/about/guidelines.xhtml

Quality of Life in elderly ICU survivors:
A Rapid Systematic Review and Meta-Analysis of Cohort Studies.

Strengths and limitations of this study

- Although it is commonly accepted that ICU mortality rates increase with age, it is less clear whether elderly adults who survive ICU can expect a reasonable quality of life (QoL). To our knowledge, this is the first systematic review and meta-analysis to explore quality of life outcomes in elderly ICU survivors. We also believe this systematic review is the first attempt to explore sources of variation between these studies.
- While mortality rates were high, elderly patients who survived ICU did not experience significantly impaired QoL at follow up, compared to before ICU or their healthy peers. Elderly patients who survive ICU can be expected to have slightly worse QoL compared to younger patients, especially in the long-term.
- We could estimate the population QoL with reasonable precision, as evidenced by narrow confidence intervals. Wide prediction intervals suggest that our results should not be used to make individual-level predictions.
- Moderation analyses suggested possible subgroups of elderly patients who have a worse QoL prognosis following ICU. However, this data was reported inconsistently and often at study-level, so we could not explore most of the outcomes for specific clinical and demographic subgroups. Future research with individual-level data will be needed to better stratify these outcomes.
- To ensure consistency and policy relevance, we only included one type of measure within the meta-analysis (EQ-5D). Where possible we converted SF-36 scores to EQ-5D using an established mapping algorithm, previously used by the National Institute for Care Excellence (NICE).

For peer review only

Quality of Life in elderly ICU survivors:
A Rapid Systematic Review and Meta-Analysis of Cohort Studies.

INTRODUCTION

The influence that age should have upon intensive care decision making has been debated across policy and clinical practice [1 2]. Age associates (inversely) with the probability of intensive care unit (ICU) survival and length of life after ICU [3 4], outcomes generally considered to be relevant to resource allocation [2]. However age is also a protected characteristic in several jurisdictions, and in England and Wales, resource allocation based on age must be a “proportionate means of achieving a legitimate aim” , if it is not to be contrary to the Equality Act (2010) .

For elderly patients for whom admission to ICU is clinically appropriate, an important part of person-centred decision-making is for them, or their families, to be given information about the likely outcome of admission. Patients may seek to integrate survival and biomedical outcomes with subjective outcomes, including quality of life (QoL). Subjective QoL in elderly ICU survivors has been studied less frequently than these objective measures [3 5]. This is notable given that subjective QoL (via Quality-Adjusted Life Years, or QALYs) is very influential in clinical resource allocation (e.g. NICE). Person-centred decision making requires consideration of patient experience since physician-rated quality of life is not always well correlated with patient-rated quality of life .

We considered a rapid review to be urgent because age is a strong risk factor for severe COVID-19 infection [6] and severe COVID-19 has placed considerable pressure on ICU resource allocation. [7] and is likely to do so in the future. Additionally, some have expressed concerns that elderly adults may be disproportionately less likely to receive ICU [1 2 8-10]. It is therefore important older persons’ subjective outcomes are better understood.

We conducted a meta-analysis on patient reported QoL in elderly adults undergoing ICU. Following a systematic review, we addressed three questions:

1) At follow up, do elderly ICU survivors have better/worse QoL compared to their scores before ICU?
2) At follow up, do elderly ICU survivors have better/worse QoL than age-matched community controls?
3) At follow up, do elderly ICU survivors have better/worse QoL than ICU survivors aged under 65?

Determining the effect of illness and ICU on QoL is complicated because QoL is itself influenced by many variables [11] and some are non-clinical. These influences are too complex to resolve completely, but where possible, we sought to model relevant variables (illness severity, ICU length of stay and mortality rate) as predictors of QoL in elderly ICU survivors at follow up, compared to controls.

For peer review only - http://bmjopen.bmj.com/site/about/guidelines.xhtml

Quality of Life in elderly ICU survivors:
A Rapid Systematic Review and Meta-Analysis of Cohort Studies.

METHODS

SEARCH STRATEGY

We searched for English-language journal articles, published between January 2000 and April 2020. Six online bibliographic databases were used: CENTRAL, CINAHL, Cochrane Library, EMBASE, MEDLINE and PsycINFO. Our search followed pre-published PROSPERO protocol (ID: CRD42020181181).

The search terms focused on intensive care, elderly adults and QoL. We supplemented this with a forward citations and reference list search based on the eligible articles as well as consultation with experts.

SELECTION CRITERIA

We undertook study selection using EndNote X9 using a standardised CRIB sheet. At the title and abstract level, we identified potentially eligible studies that took place in an ICU and referred to either QoL life or elderly adults. Full texts were eligible if a) all participants underwent ICU; b) there were at least 20 elderly patients and controls; c) scores from a validated QoL scale were reported, for a group aged at least 60+, with at least 3 months follow up review; d) the follow up QoL scores were derived from the patient, rather than a professional ; and e) the study reported QoL scores from the same scale for either the same patients before the ICU admission, age-matched community controls or ICU survivors aged under 65.

We considered whether to include studies that focused only on cardio or neuro-surgical patients, given the effects of the diagnostic heterogeneity that characterises the reference population of the studies included in our review (general ICU patients with various conditions). However, none of these studies met the other inclusion criteria.

K.A led the study selection at all stages and a post-doctoral research assistant conducted reliability checks for 50% of full text articles. We found nearly perfect inter-rater agreement, as measured by Cohen's kappa (k= .86) [12]. Queries were resolved by G.O.

DATA EXTRACTION

One reviewer (K.A) extracted relevant data from all eligible studies, recording this on a standardised spreadsheet. M.K. independently extracted data from 10% of eligible studies, to evaluate consistency. The primary outcome was the QoL composite scores. Secondary variables included demographics, QoL subscale scores, mortality (from ICU to follow up), illness severity (APACHE-II or SAPS-II), length of ICU stay, length of hospital stay, and average follow up time. When one dataset was used for multiple studies, we included the study with the clearest data reporting.

To ensure consistency, we included only composite scores from the EuroQoL health related quality of life instrument (EQ-5D) within the meta-analysis. Where possible, we also converted the eight SF-36 subscales to an EQ-5D index score, using an established mapping algorithm. [13] The remaining studies were included within the qualitative synthesis only.

For peer review only - http://bmjopen.bmj.com/site/about/guidelines.xhtml

Quality of Life in elderly ICU survivors:
A Rapid Systematic Review and Meta-Analysis of Cohort Studies.

DATA ANALYSIS

We explored the effect of age on EQ-5D composite scores using random effects meta-analyses. KA conducted the analysis using R Statistics. We used the Restricted Maximum Likelihood (REML) method to calculate the effect sizes (Cohen's d), which were weighted by the inverse of the sampling variance: meaning that studies with higher variance contributed less to the summary effect size. We interpreted these effect sizes using conventional criteria as a guide (0.2 = small; 0.5 = medium; 0.8 = large) [14]. We then conducted sensitivity analyses for each meta-analysis to assess risk of bias at the study level, including heterogeneity (e.g. I^2 statistic), influential studies (e.g. Cook's distance), and publication bias (funnel plots and Egger's test).

To investigate the remaining heterogeneity, we then conducted two secondary analyses: a moderator analysis to explore variation within a specific predictor, and a random-effects meta-regression to explore relationships between multiple predictors.

We used several strategies to handle missing data. When the study only reported median values and interquartile ranges, we estimated the mean and standard deviation using conventional formulae [15 16]. When neither the standard deviation nor interquartile range was reported, we estimated the standard deviation using prognostic imputation [17]. This calculates the average of observed variances to estimate the missing standard deviation values. We excluded studies with missing data if these methods were inapplicable.

One reviewer (K.A) assessed the methodological rigour of the included studies using an 11-item quality checklist (three irrelevant items were excluded) [18]. The criteria were scored as either 2 (complete fulfilment), 1 (partial fulfilment) or 0 (not fulfilled). We then calculated a total score for each study and rated them as either high quality (17/22 or higher), moderate quality (between 10/22 and 16/22) or low quality (9/22 or lower). Queries were resolved through discussion with G.O and S.C.

For the qualitative synthesis, we defined a set of criteria for each measure to allocate subscores to either 'mental health' or 'physical health' categories. We then calculated a crude average for subscales within these two categories and weighted them on a scale of 1-100 (0= minimum QoL; 100 = maximum QoL). As this approach is subjective, we present these findings only as a qualitative supplement.

This study follows methodological guidance from PRISMA.

For peer review only

For peer review only - http://bmjopen.bmj.com/site/about/guidelines.xhtml

Quality of Life in elderly ICU survivors:
A Rapid Systematic Review and Meta-Analysis of Cohort Studies.

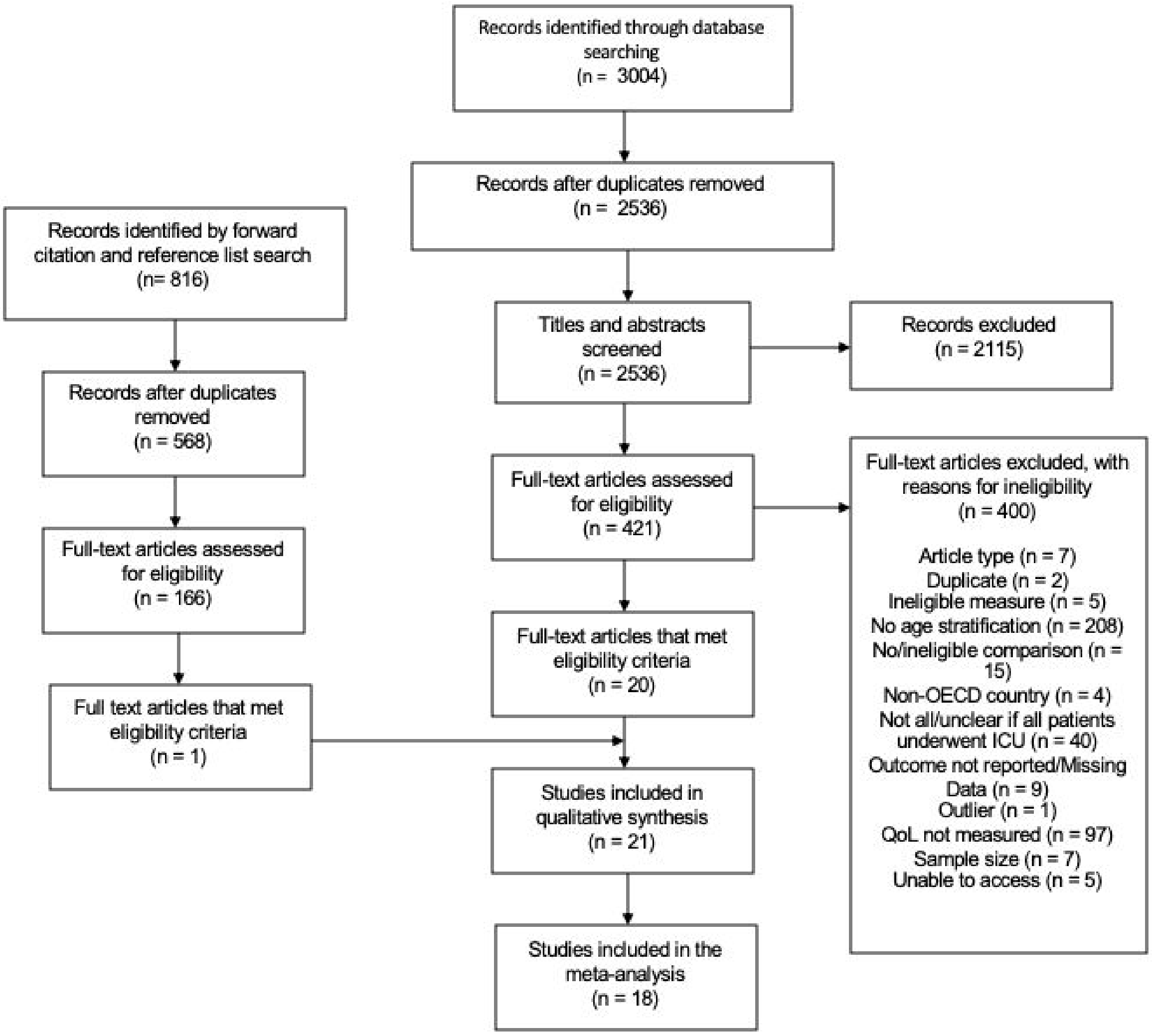

Figure 1. A PRISMA flow diagram that outlines the study selection process.

For peer review only - http://bmjopen.bmj.com/site/about/guidelines.xhtml

Quality of Life in elderly ICU survivors:
A Rapid Systematic Review and Meta-Analysis of Cohort Studies.

RESULTS

DESCRIPTIVE STATISTICS

After screening duplicates, the database search revealed 2536 records for title and abstract screening. From these, we reviewed 421 potentially relevant full text articles for eligibility. 18 studies met the full criteria and were included in the meta-analysis (N= 2090 elderly ICU survivors) [19-39]. Eight of these studies reported age characteristics for the elderly patients (M= 78.53, SD= 4.17), while the others reported the minimum age only.

Most of the studies included both medical and surgical ICU patients (fifteen studies). The remaining studies focused on surgical (two studies) or medical (one study) patients only. Three types of outcome were included in the meta-analysis. These results compared QoL at follow up to either pre-ICU scores (five studies), age-matched community controls (nine studies), or younger survivors of ICU (six studies). We provide a full summary in Table 1.

For the qualitative analysis, four different measurement scales were reported: the EuroQoL EQ-5D health related quality of life instrument (ten studies), the short form medical outcome questionnaire (SF-36; eight studies), the Nottingham health profile (NHP; one study), the quality of life index (QLI; one study) and the World Health Organisation quality of life instruments (WHO-QOL-BREF; one study). SF-36 scores were converted to EQ-5D index scores for the meta-analysis, while the other measures were excluded (see methods).

For peer review only

Quality of Life in elderly ICU survivors:
A Rapid Systematic Review and Meta-Analysis of Cohort Studies.

First Author	Year	Country	N	Min Age	% Male	Follow-up (avg. months)	ICU LoS (days)	Mortality	Severity (scaled avg.)	Raw Measure	Comparison	Quality
Abelha[39]	2007	Portugal	114	65+	61.00%	6		28.00%		SF-36 [*]	ICU survivors younger than 65 years old	M
Ali [38]	2018	Australia	32	65+	80.00% [a]	12	5		.24	EQ-5D	Age-matched South Australian controls	H
Andersen [37]	2015	Norway	53	80+	69.00%	40.8	1.9	81.52%	.27	EQ-5D	Age and sex-matched Norwegian population	M
Cuthbertson [36]	2005	UK	62	65+	59.00%[a]	12		33.00%		SF-36 [*]	ICU survivors younger than 65 years old	M
De Rooij [35]	2008	Netherlands	187	80+	51.00%	44.4	1.29	61.52%	.21	EQ-5D	Age-matched British population	M
Eddleston [34]	2000	UK	39	65+	52.45%[a]	3				SF-36 [*]	ICU survivors younger than 65 years old	M
Ferrao [33]	2015	Portugal	290	66+ [b]	26.00%	27.6				EQ-5D	ICU survivors younger than 65 years old	M
Grace [31]	2007	Australia/NZ	99	60+	NR	28		60.00%	.28	EQ-5D	Retrospective patient ratings for one week before ICU	L
Hofhuis [30]	2011	Netherlands	49	80+ [b]	46.90%	6	5.35	40.83%	.25	SF-36 [*]	Age-matched Dutch population	M
Jeitziner [29]	2015	Switzerland	124	65+	73.00%	12	4.57		.29	SF-36 [*]	Retrospective proxy ratings for four weeks before ICU<br>Age matched Swiss controls;<br>Retrospective patient ratings for one week before ICU	M
Kaarola [28]	2006	Finland	299	65+	75.00%	47		57.00%		EQ-5D	ICU survivors younger than 65 years old	M
Levinson [26]	2016	Australia	322	80+	58.00%[a]	24	1.28	21.45%		SF-36 [*]	Age and sex-matched Australian population	H
Merlani [25]	2007	Switzerland	36	70+	52.00%	24	3.00	63.00%	.26	EQ-5D	Age-matched Swiss population	M
Oeyen [24]	2007	Netherlands	63	80+	60.00%[a]	12		49.60%	.26	EQ-5D	Retrospective patient or proxy ratings for one week before ICU	M
Sacanella [23]	2011	Spain	112	65+	57.00%	12	3.35	48.70%	.27	EQ-5D	Retrospective patient or proxy ratings before feeling ill and requiring ICU	M
Schroder [22]	2011	Denmark	36	75+	56.00%	12	9.4	53.85%		SF-36 [*]	Age-matched Danish population	L
Sznajer [21]	2001	France	65	65+ [b]	55.90%[a]	6				EQ-5D	ICU survivors younger than 65 years old	M
Villa [19]	2016	Spain	54	75+	50.00%	12		43.18%	.23	SF-36 [*]	Spanish population aged 75+	M
Weighted avg.			*108.05*	*71.23*	*55.67%*	*23.43*	*3.63*	*46.01%*	*.23*			
Range			*23-322*	*60-80*	*26-80%*	*3-100.8*	*1.28-12.6*	*21.45-81.52%*	*.12-.34*			

Table 1. The main characteristics of the studies and the relevant data included in the meta-analyses.
[a] Reported for study level only, so not included in meta-analysis
[b] Combined elderly groups
[*] Converted to EQ-5D composite score
Abbreviations: ICU (intensive care unit); LoS (length of stay); H = High quality; M= Moderate quality; L= Low quality. See above for measures.
Unless specified, we do not report data where it is not representative of at least 66.67% of the included sample.

For peer review only - http://bmjopen.bmj.com/site/about/guidelines.xhtml

Quality of Life in Older Adults following Intensive Care:
A Rapid Systematic Review of Cohort Studies.

META ANALYSES

Comparison	k	Cohen d	95% CI	95% PI	P	I^2
Pre-ICU scores	6	-.18	-.39, .03	-.65, .29	.097	67.91%
Community	9	-.15	-.31, .01	-.58, .28	.075	70.06%
Under 65s	5	-.33	-.55, -.10	-.83, .18	.006	81.93%

Table 2. A summary of effect sizes, confidence intervals, prediction intervals, significance and heterogeneity for each meta-analysis (k= number of independent samples, I^2= between study heterogeneity)

Table 2 outlines the results of the three meta analyses. There was no significant difference in EQ-5D composite scores between elderly patients before and after ICU (d= -.18, n.s). There was also no significant difference in EQ-5D composite scores between elderly ICU survivors and age-matched community controls (d= -.15, n.s). These results suggest that there were no average differences in QoL between these groups.

Elderly ICU survivors (aged over 65) had significantly lower composite scores on the EQ-5D, compared to younger ICU survivors (aged under 65), with a small-to-medium effect size (d= -.33, p= <.01). This suggests that on average, QoL in elderly ICU survivors is slightly worse than younger ICU survivors.

SENSITIVITY ANALYSES

We reviewed the impact of influential cases within each analysis. One study was excluded from the community meta-analysis as a substantial outlier and influential result [40]. If the result had not been excluded, the effect size would have been stronger (d= -1.80 – ie a larger difference in QoL favouring younger controls) but non-significant (p= .27), mainly due to large heterogeneity (I^2 = 100%). It is unclear why this study reported substantially outlying results, although the reported standard deviations were considerably lower than other studies.

After excluding this, one other study was marginally influential within the community analysis (see Appendix). [29] This study was retained as the between study heterogeneity was moderate and excluding the case would have had little impact on the effect size or interpretation. We identified no further outliers according to our criteria.

SECONDARY ANALYSES

There was moderate-to-large heterogeneity between studies, therefore we explored the role of other variables using post-hoc subgroup analyses and meta-regressions. These results should be interpreted with caution, due to low sample sizes.

Length of follow up significantly predicted greater differences in QoL between elderly ICU survivors and patients aged under 65 (k= 5, p< .0001). This suggests that elderly survivors may have worse QoL in the long term and comparable QoL in the medium term.

Mortality rate significantly predicted greater differences in QoL between elderly ICU survivors and age-matched community controls (k= 7, p= .01). This revealed that elderly patients had worse QoL than controls in studies with high mortality rates, compared to studies with low mortality rates.

For peer review only - http://bmjopen.bmj.com/site/about/guidelines.xhtml

Quality of Life in Older Adults following Intensive Care:
A Rapid Systematic Review of Cohort Studies.

Controlling for these variables reduced heterogeneity between studies to 0% in both cases. No model significantly accounted for variance when the outlier [40] was included in the community analysis.

Neither severity of illness, year of publication, sex nor minimum age significantly accounted for heterogeneity between the studies, either individually or within a meta-regression (p> .05).

RISK OF BIAS

We found no evidence of publication bias for the community or pre-ICU meta-analyses, from either funnel plots or Egger's test (all p> .05). Most studies had a moderate degree of methodological quality (14/18). We had insufficient power to explore the effect of study quality on quantitative outcomes.

QUALITATIVE SYNTHESIS

To compare different aspects of QoL, we categorised the subscales into either mental or physical health QoL and calculated a scaled average to enable comparisons (see Table 3). 16/21 studies reported the subscales for both conditions. Our estimates suggest that elderly ICU survivors reported higher average scores on mental health items (M= 57.90/100) than physical health items (M= 50.99/100). Trends in physical health scores compared less favourably to age-matched community controls than did mental health scores (mean differences = -5.23 and -1.71, respectively). Trends in physical health scores were also lower in comparison to younger ICU controls (mean difference = -1.40) whereas mental health scores were higher (mean difference = 2.98).

For peer review only

For peer review only - http://bmjopen.bmj.com/site/about/guidelines.xhtml

Quality of Life in Older Adults following Intensive Care:
A Rapid Systematic Review of Cohort Studies.

First Author	Comparison	Measure	Mean MH (Elder ICU survivor)	Mean MH (Comparison)	Mean Difference	Mean PH Score (Elder ICU survivor)	Mean PH (Comparison)	Mean Difference
Anderson	Community	EQ5D	58.62	55.87	2.75	47.27	48.46	-1.19
De Rooij	Community	EQ5D	56.86	58.22	-1.35	48.89	50.49	-1.60
Merlani	Community	SF36	43.00	47.00	-4.00	36.00	42.00	-6.00
Jeitziner	Community	SF36	69.72	80.37	-10.65	62.71	77.91	-15.20
Villa	Community	SF36	62.40	61.50	0.90	66.60	67.90	-1.30
Garrouste-Orgeas	Community	NHP	67.13	83.00	-15.87	53.63	70.23	-16.60
Schroder	Community	SF36	56.93	54.30	2.64	38.36	43.71	-5.35
Tabah	Community	WHOQOL	73.30	61.40	11.90	62.10	56.70	5.40
Average	*Community*		*61.00*	*62.71*	*-1.71*	*51.94*	*57.18*	*-5.23*
Grace	PreICU	EQ5D	61.67	54.00	7.67	58.50	53.22	5.28
Cuthbertson	PreICU	SF36	51.40	50.80	0.60	37.30	31.40	5.90
Hofhuis	PreICU	SF36	51.20	50.10	1.10	38.60	38.80	-0.20
Jeitziner	PreICU	SF36	69.72	69.02	0.70	62.71	63.63	-0.92
Average	*PreICU*		*58.50*	*55.98*	*2.52*	*49.28*	*46.76*	*2.51*
Abelha	Young	SF36	48.50	47.50	1.00	46.50	48.50	-2.00
Cuthbertson	Young	SF36	51.40	51.30	0.10	37.30	37.50	-0.20
Hofhuis	Young	SF36	51.20	50.40	0.80	38.60	38.70	-0.10
Schroder	Young	SF36	56.93	54.30	2.64	38.36	43.71	-5.35
Eddleston	Young	SF36	63.59	58.58	5.01	58.76	63.25	-4.49
Kleinpell	Young	QLI	76.26	67.93	8.32	66.33	62.60	3.73
Average	*Young*		*57.98*	*55.00*	*2.98*	*47.64*	*49.04*	*-1.40*

Table 3. An overview of Quality of Life subscores, by mental health and physical health categories, for elderly ICU survivors and comparison groups. All scores were recalculated on a 0-100 (0 = minimum QoL; 100 = maximum QoL).
Abbreviations: MH= Mental Health; PH=Physical Health

For peer review only - http://bmjopen.bmj.com/site/about/guidelines.xhtml

Quality of Life in Older Adults following Intensive Care:
A Rapid Systematic Review of Cohort Studies.

DISCUSSION

This review has systematically evaluated the literature on QoL for elderly ICU survivors in the medium to long term, using EQ-5D composite scores. To our knowledge this is the first meta-analysis to address this issue. We found no evidence of worse QoL after ICU, compared to a period before ICU or compared to healthy community peers. However, elderly patients who survive ICU can be expected to have slightly worse QoL, compared to younger survivors. The wide prediction intervals also suggest that age differences can vary considerably in either direction.

STRENGTHS IN RELATION TO THE LITERATURE

For the meta-analysis, we identified 2090 elderly ICU survivors within an international sample of 18 cohort studies. We only included recent studies that used validated QoL measures and we rated most studies as having moderate or higher methodological quality. By pooling these samples using rigorous methods, we have been able to overcome several methodological limitations associated with generalising from individual studies, including small samples, choice of analysis and site selection bias. Our sensitivity analyses showed that the remaining heterogeneity was mostly due to conceptually relevant variables. Given the relatively small literature, these methods ensure that valid, transparent results inform policy and clinical practice decisions.

Although contested, previous reviews have generally concluded that age alone is not a suitable determinant of potential benefit from ICU, especially for survivors [3 5 41 42] . The present study supports these conclusions overall, although the differences compared to younger ICU survivors are still noteworthy. Decisions on whether to admit patients can be extremely difficult for all involved, with seriously ill elderly people overrepresented among the most contentious cases [43]. These challenges are amplified further when healthcare resources are under pressure, such as during the COVID-19 pandemic.

The age-QoL associations we have found may be explained by intermediary variables. Some research suggests that frailty may best explain age differences in QoL following ICU [5 44], and clinical outcome in COVID-19 patients [45]. Frailty is a more integrative approach to conceptualising ageing, but it was not reported within the eligible studies. We would recommend a meta-analysis of individual patient data to further stratify clinical variables of interest, including frailty, to better predict QoL outcomes.

Health economic analysis of ICU in the elderly based on QALYs may be informative when it comes to resource allocation policies but we have found few such analyses and no explicit polices based on them. They will have to grapple with the controversial notion that everyone is entitled to a ‘normal’ span of health or ‘a fair innings’ [46 47]. Given the presumption that a sizeable proportion of elderly survivors will enjoy a good QoL it is crucial that holistic, person-centred decision making is not crowded out by survival statistics or anticipatory triage. If triage were to become necessary on the front line we would advise against weighing age too heavily and rather taking more account of frailty after appropriate consultations.

On average, QoL scores gradually decline with age at approximately 0.5 points per year on the CASP-19 (range 0-57) with a modestly accelerated decrease with older age (>85 years) [4]. It is relevant to consider whether change in QoL in the elderly is primarily due to physical

For peer review only - http://bmjopen.bmj.com/site/about/guidelines.xhtml

Quality of Life in Older Adults following Intensive Care:
A Rapid Systematic Review of Cohort Studies.

health and mental health components. We were unable to incorporate physical and mental subscores into the meta-analysis due to differences in the levels of data between measures, so we performed a qualitative synthesis. This suggested that for elderly ICU survivors, mental health questionnaire items were relatively unaffected. The small literature on older adults also suggests relatively low rates of anxiety [48] and depressive disorders [49 50], although potentially high rates of post-traumatic stress. [51] Together with previous research, which found that elderly people typically value their psychosocial wellbeing above their physical needs [52] our results highlight the importance of caution with assumptions on age as a determinant of poor quality of life following ICU.

LIMITATIONS

The primary limitation is the small number of eligible studies for each analysis. To maximise the sample, we included some studies with a small amount of missing data and used validated methods to estimate the mean or the standard deviation from the reported statistics. We argue that these approaches are justified as, based on central limit theorem, we expect the larger sample sizes to produce a better estimate of population variance [53]. For balance, we have also provided a comprehensive overview of our sensitivity analyses to assess risk of bias (see Appendix). These demonstrate that although our decisions reduced bias, most did not change our interpretation of the effects.

Another potential limitation of the meta-analysis is the focus on long-term ICU survivors, as reported mortality rates were as high as 80% at follow up. We argue that a substantial 'healthy survivor' effect on QoL is unlikely because survival and QoL have different pathophysiological determinants. We also did not find any evidence of better QoL for elderly patients in studies with high mortality rates. Nevertheless, our results clearly extend only to ICU survivors, rather than prospective ICU patients.

Our results may also be prone to other selection biases. Compared to younger adults, unhealthy elderly adults might be less likely to be admitted to into ICU [32 43], to survive ICU treatment (possibly in part due to decisions around lifesaving treatment [54]) and to survive until follow-up. It was also unclear how many patients had pre-existing cognitive impairments where QoL measurement is more complex, although there was no indication that the proportion was large. As a result, we would caution wider generalisations to all elderly ICU patients. Nonetheless, these results imply that at least a sizeable subgroup of elderly ICU patients will report subjective outcomes that compare well to groups that might be expected to fare better.

We were unable to assess change in quality of life as rigorously as we would have liked. Ideally, we would have analysed differences in QoL change scores between younger and elderly ICU survivors, at multiple time points from before ICU to follow up. The scores for pre-ICU scores were also problematic, as these were determined by retrospective ratings from discharged patients or proxies. This is usual practice, but the reliability of proxies is contested [55 56].

Finally, we observed moderate-to-high levels of heterogeneity between studies, which limits the generalisability of the results. We found that much of this variation may have been due to mortality rates and length of time post-discharge, which supports the view that age alone is not a strong predictor of QoL outcome. We also tried to ensure consistency of measurement

For peer review only - http://bmjopen.bmj.com/site/about/guidelines.xhtml
For peer review only

by using a mapping function between SF-36 scores to EQ-5D scores, which is a common approach within NICE guidelines[13 57].

For peer review only

For peer review only - http://bmjopen.bmj.com/site/about/guidelines.xhtml

Quality of Life in Older Adults following Intensive Care:
A Rapid Systematic Review of Cohort Studies.

CONCLUSION

Our study reports the first known meta-analysis of quality of life in elderly patients following ICU. We report that on average, elderly survivors of ICU have similar QoL after ICU compared to before and that their QoL is comparable to their community peers. They have slightly worse QoL compared to younger ICU survivors based on physical rather than mental health, but it does not change for the worse following ICU. These findings add rigour to the current literature and should inform debates around population level resource allocation and person-centred intensive care decision making during the current COVID-19 pandemic and after.

CORRESPONDENCE

Mr. Kevin Ariyo MSc (PhD candidate), Mental Health and Justice Project, Department of Psychological Medicine, Institute of Psychiatry, Psychology and Neuroscience, King's College London. 16 De Crespigny Park, Camberwell, London, SE5 8AF. Email: kevin.ariyo@kcl.ac.uk.

AUTHOR STATEMENT

K.A. led at each stage of the project, including drafting the document. G.O. was primary supervisor on the project, jointly formulated the research questions, led on writing the introduction section and made substantial contributions to all aspects of the study. S.C. advised on the initial protocol and provided critical revisions from an intensivist perspective. A.D. and A.R.K. provided additional supervision and critical revisions.

The manuscript is a transparent account of the study being reported and adheres to PRISMA reporting guidelines. All listed authors have approved for the manuscript to be published in its current format and meet all the ICMJE criteria for authorship. The authors agree to be accountable for the contents of the paper and are jointly responsible for ensuring that all queries related to the accuracy or integrity of the project are investigated and resolved.

ACKNOWLEDGEMENTS

We are grateful to Margot Kuylen for her contributions to the reliability assessment and to John Brazier for advising on the SF-36 to EQ-5D mapping function.

ETHICAL APPROVAL

Not required.

DATA SHARING

The datasets generated and analysed during the current study are included in this published article and its supplementary information files. Any data queries may also be directed to the corresponding author on reasonable request.

FUNDING

Supported by the Mental Health and Justice Project, led by G.O., which is funded by a grant from the Wellcome Trust (203376/2/16/Z).

For peer review only

Quality of Life in Older Adults following Intensive Care:
A Rapid Systematic Review of Cohort Studies.

REFERENCES

Manuscript Only

1. Archard D, Caplan A. Is it wrong to prioritise younger patients with covid-19? *BMJ* 2020;369
2. Savulescu J, Cameron J, Wilkinson D. Equality or utility? Ethics and law of rationing ventilators. *British Journal of Anaesthesia* 2020
3. Hennessy D, Juzwishin K, Yergens D, et al. Critical care review. Outcomes of elderly survivors of intensive care: a review of the literature. *CHEST* 2005;127(5):1764-74.
4. Zaninotto P, Falaschetti E, Sacker A. Age trajectories of quality of life among older adults: results from the English Longitudinal Study of Ageing. *Quality of Life Research* 2009;18(10):1301-09.
5. Conti M, Merlani P, Ricou B. Prognosis and quality of life of elderly patients after intensive care. *Swiss Med Wkly* 2012;142:w13671. doi: 10.4414/smw.2012.13671 [published Online First: 2012/09/12]
6. Zhou F, Yu T, Du R, et al. Clinical course and risk factors for mortality of adult inpatients with COVID-19 in Wuhan, China: a retrospective cohort study. *The lancet* 2020
7. Willan J, King AJ, Jeffery K, et al. Challenges for NHS hospitals during covid-19 epidemic: British Medical Journal Publishing Group, 2020.
8. de Castro-Hamoy L, de Castro LD. Age Matters but it should not be Used to Discriminate Against the Elderly in Allocating Scarce Resources in the Context of COVID-19. *Asian Bioethics Review* 2020:1-10.
9. Haas LE, de Lange DW, van Dijk D, et al. Should we deny ICU admission to the elderly? Ethical considerations in times of COVID-19. *Critical Care* 2020;24(1):1-3.
10. Montero-Odasso M, Hogan DB, Lam R, et al. Age alone is not adequate to determine healthcare resource allocation during the COVID-19 pandemic. *Canadian Geriatrics Journal* 2020;23(1):152-54.
11. Rubenfeld GD. Does the hospital make you older faster?: American Thoracic Society, 2012.
12. Landis JR, Koch GG. The measurement of observer agreement for categorical data. *biometrics* 1977:159-74.
13. Ara R, Brazier J. Deriving an algorithm to convert the eight mean SF-36 dimension scores into a mean EQ-5D preference-based score from published studies (where patient level data are not available). *Value in Health* 2008;11(7):1131-43.
14. Cohen J. Statistical power analysis for the behavioral sciences New York. *NY: Academic* 1988
15. Luo D, Wan X, Liu J, et al. Optimally estimating the sample mean from the sample size, median, mid-range, and/or mid-quartile range. *Statistical methods in medical research* 2018;27(6):1785-805.
16. Wan X, Wang W, Liu J, et al. Estimating the sample mean and standard deviation from the sample size, median, range and/or interquartile range. *BMC medical research methodology* 2014;14(1):135.
17. Ma J, Liu W, Hunter A, et al. Performing meta-analysis with incomplete statistical information in clinical trials. *BMC medical research methodology* 2008;8(1):56.
18. Kmet LM, Cook LS, Lee RC. Standard quality assessment criteria for evaluating primary research papers from a variety of fields. 2004
41. Chelluri L, Grenvik A, Silverman M. Intensive care for critically ill elderly: mortality, costs, and quality of life: review of the literature. *Archives of Internal Medicine* 1995;155(10):1013-22.

For peer review only - http://bmjopen.bmj.com/site/about/guidelines.xhtml

Quality of Life in Older Adults following Intensive Care:
A Rapid Systematic Review of Cohort Studies.

42. Wehler M. [Long-term outcome of elderly patients after intensive care treatment]. *Med Klin Intensivmed Notfmed* 2011;106(1):29-33. doi: 10.1007/s00063-011-0021-x [published Online First: 2011/10/07]
43. Escher M, Cullati S, Hudelson P, et al. Admission to intensive care: A qualitative study of triage and its determinants. *Health Serv Res* 2019;54(2):474-83. doi: 10.1111/1475-6773.13076 [published Online First: 2018/10/27]
44. Heyland DK, Garland A, Bagshaw SM, et al. Recovery after critical illness in patients aged 80 years or older: a multi-center prospective observational cohort study. *Intensive care medicine* 2015;41(11):1911-20.
45. Hewitt J, Carter B, Vilches-Moraga A, et al. The effect of frailty on survival in patients with COVID-19 (COPE): a multicentre, European, observational cohort study. *The Lancet Public Health* 2020
46. Williams A. Intergenerational equity: an exploration of the 'fair innings' argument. *Health economics* 1997;6(2):117-32.
47. NICE CITIZENS COUNCIL REPORT ON AGE: National Institute for Care Excellence, 2020.
48. Jeitziner MM, Hamers JP, Bürgin R, et al. Long-term consequences of pain, anxiety and agitation for critically ill older patients after an intensive care unit stay. *Journal of clinical nursing* 2015;24(17-18):2419-28.
49. Broslawski G, Elkins M, Algus M. Functional abilities of elderly survivors of intensive care. *The Journal of the American Osteopathic Association* 1995;95(12):712.
50. Chelluri L, Pinsky MR, Donahoe MP, et al. Long-term Outcome of Critically III Elderly Patients Requiring Intensive Care. *Jama* 1993;269(24):3119-23.
51. Drews T, Franck M, Radtke FM, et al. Postoperative delirium is an independent risk factor for posttraumatic stress disorder in the elderly patient: a prospective observational study. *European Journal of Anaesthesiology (EJA)* 2015;32(3):147-51.
52. Netuveli G, Blane D. Quality of life in older ages. *British medical bulletin* 2008;85(1):113-26.
53. Weir CJ, Butcher I, Assi V, et al. Dealing with missing standard deviation and mean values in meta-analysis of continuous outcomes: a systematic review. *BMC medical research methodology* 2018;18(1):25.
54. Ferrand E, Robert R, Ingrand P, et al. Withholding and withdrawal of life support in intensive-care units in France: a prospective survey. *The Lancet* 2001;357(9249):9-14.
55. Capuzzo M, Grasselli C, Carrer S, et al. Quality of life before intensive care admission: agreement between patient and relative assessment. *Intensive care medicine* 2000;26(9):1288-95.
56. Dinglas VD, Gifford JM, Husain N, et al. Quality of life before intensive care using EQ-5D: patient versus proxy responses. *Critical care medicine* 2013;41(1):9.
57. Longworth L, Rowen D. Mapping to obtain EQ-5D utility values for use in NICE health technology assessments. *Value in health* 2013;16(1):202-10.

Search Results

19. Villa P, Pintado MC, Lujan J, et al. Functional Status and Quality of Life in Elderly Intensive Care Unit Survivors. *J Am Geriatr Soc* 2016;64(3):536-42. doi: 10.1111/jgs.14031 [published Online First: 2016/03/24]
20. Tabah A, Philippart F, Timsit JF, et al. Quality of life in patients aged 80 or over after ICU discharge. *Crit Care* 2010;14(1):R2. doi: 10.1186/cc8231 [published Online First: 2010/01/13]

For peer review only - http://bmjopen.bmj.com/site/about/guidelines.xhtml

Quality of Life in Older Adults following Intensive Care:
A Rapid Systematic Review of Cohort Studies.

21. Sznajder M, Aegerter P, Launois R, et al. A cost-effectiveness analysis of stays in intensive care units. *Intensive Care Med* 2001;27(1):146-53. doi: 10.1007/s001340000760 [published Online First: 2001/03/31]
22. Schroder MA, Poulsen JB, Perner A. Acceptable long-term outcome in elderly intensive care unit patients. *Dan Med Bull* 2011;58(7):A4297. [published Online First: 2011/07/05]
23. Sacanella E, Perez-Castejon JM, Nicolas JM, et al. Functional status and quality of life 12 months after discharge from a medical ICU in healthy elderly patients: a prospective observational study. *Crit Care* 2011;15(2):R105. doi: 10.1186/cc10121 [published Online First: 2011/03/30]
24. Oeyen S, Vermassen J, Piers R, et al. Critically ill octogenarians and nonagenarians: evaluation of long-term outcomes, posthospital trajectories and quality of life one year and seven years after ICU discharge. *Minerva Anestesiol* 2017;83(6):598-609. doi: 10.23736/S0375-9393.16.11434-8 [published Online First: 2016/12/03]
25. Merlani P, Chenaud C, Mariotti N, et al. Long-term outcome of elderly patients requiring intensive care admission for abdominal pathologies: survival and quality of life. *Acta Anaesthesiol Scand* 2007;51(5):530-7. doi: 10.1111/j.1399-6576.2007.01273.x [published Online First: 2007/04/14]
26. Levinson M, Mills A, Oldroyd J, et al. The impact of intensive care in a private hospital on patients aged 80 and over: health-related quality of life, functional status and burden versus benefit. *Intern Med J* 2016;46(6):694-702. doi: 10.1111/imj.13079 [published Online First: 2016/03/25]
27. Kleinpell RM, Ferrans CE. Quality of life of elderly patients after treatment in the ICU. *Res Nurs Health* 2002;25(3):212-21. doi: 10.1002/nur.10035 [published Online First: 2002/05/17]
28. Kaarlola A, Tallgren M, Pettila V. Long-term survival, quality of life, and quality-adjusted life-years among critically ill elderly patients. *Crit Care Med* 2006;34(8):2120-6. doi: 10.1097/01.CCM.0000227656.31911.2E [published Online First: 2006/06/10]
29. Jeitziner MM, Zwakhalen SM, Burgin R, et al. Changes in health-related quality of life in older patients one year after an intensive care unit stay. *J Clin Nurs* 2015;24(21-22):3107-17. doi: 10.1111/jocn.12904 [published Online First: 2015/08/08]
30. Hofhuis JGM, van Stel HF, Schrijvers AJP, et al. Changes of health-related quality of life in critically ill octogenarians: a follow-up study. *Chest* 2011;140(6):1473-83. doi: 10.1378/chest.10-0803 [published Online First: 2011/10/01]
31. Grace RF, Gosley M, Smith P. Mortality and outcomes of elderly patients admitted to the intensive care unit at Cairns Base Hospital, Australia. *Critical care and resuscitation : journal of the Australasian Academy of Critical Care Medicine* 2007;9(4):334-37.
32. Garrouste-Orgeas M, Timsit JF, Montuclard L, et al. Decision-making process, outcome, and 1-year quality of life of octogenarians referred for intensive care unit admission. *Intensive Care Med* 2006;32(7):1045-51. doi: 10.1007/s00134-006-0169-7 [published Online First: 2006/06/23]
33. Ferrao C, Quintaneiro C, Camila C, et al. Evaluation of long-term outcomes of very old patients admitted to intensive care: Survival, functional status, quality of life, and quality-adjusted life-years. *J Crit Care* 2015;30(5):1150 e7-11. doi: 10.1016/j.jcrc.2015.05.005 [published Online First: 2015/07/06]
34. Eddleston JM, White P, Guthrie E. Survival, morbidity, and quality of life after discharge from intensive care. *Crit Care Med* 2000;28(7):2293-9. doi: 10.1097/00003246-200007000-00018 [published Online First: 2000/08/02]

For peer review only - http://bmjopen.bmj.com/site/about/guidelines.xhtml

35. de Rooij SE, Govers AC, Korevaar JC, et al. Cognitive, functional, and quality-of-life outcomes of patients aged 80 and older who survived at least 1 year after planned or unplanned surgery or medical intensive care treatment. *J Am Geriatr Soc* 2008;56(5):816-22. doi: 10.1111/j.1532-5415.2008.01671.x [published Online First: 2008/04/04]
36. Cuthbertson BH, Scott J, Strachan M, et al. Quality of life before and after intensive care. *Anaesthesia* 2005;60(4):332-9. doi: 10.1111/j.1365-2044.2004.04109.x [published Online First: 2005/03/16]
37. Andersen FH, Flaatten H, Klepstad P, et al. Long-term survival and quality of life after intensive care for patients 80 years of age or older. *Ann Intensive Care* 2015;5(1):53. doi: 10.1186/s13613-015-0053-0 [published Online First: 2015/06/10]
38. Ali Abdelhamid Y, Weinel LM, Hatzinikolas S, et al. Autonomic function, postprandial hypotension and falls in older adults at one year after critical illness. *Critical care and resuscitation : journal of the Australasian Academy of Critical Care Medicine* 2020;22(1):53-62.
39. Abelha FJ, Santos CC, Maia PC, et al. Quality of life after stay in surgical intensive care unit. *BMC anesthesiology* 2007;7(1):8.
40. Pavoni V, Gianesello L, Paparella L, et al. Outcome and quality of life of elderly critically ill patients: an Italian prospective observational study. *Arch Gerontol Geriatr* 2012;54(2):e193-8. doi: 10.1016/j.archger.2011.11.013 [published Online First: 2011/12/20]

For peer review only

1. STUDY CHARACTERISTICS

1.1 Meta-Analysis

First Author	Year	Country	Study Design	Journal	Setting	Min Age	Avg. Age (SD)	% Male	Mortality	ICU LoS (SD)	HLoS (SD)	Severity	Raw Measure	Follow up	Comparison	Study Quality	Participant No.	Control No.	Effect Size	Variance
Abelha[39]	2007	Portugal	Cohort (unspecified)	BMC Anaesthesiology	Surgical ICU	65+		61.00%	28.00%				SF-36*	6 months	ICU survivors younger than 65 years old	M	114	112	-.07	.02
Ali[38]	2018	Australia	Prospective Cohort	Journal of Critical Care	Medical-Surgical ICU	65+	73 (5)	80.00%[a]		4.64 (2.32)	16.29 (9.28)	.24	EQ-5D	12 months	Age-matched South Australian controls	H	32	572	.03	.03
Andersen[37]	2015	Norway	Retrospective Cohort	Annals of Intensive Care	General Hospital ICU	80+	87.4 (4)	69.00%	81.52%	1.9 (NR)		.27	EQ-5D	40.8 months	Age and sex-matched Norwegian population	M	53	170	-.18	.02
Cuthbertson[36]	2005	UK	Prospective Cohort	Critical Care	General Hospital ICU	65+		59.00%[a]	33.00%				SF-36*	12 months	ICU survivors younger than 65 years old	M	62	116	.17	.02
De Rooij[35]	2008	Netherlands	Retrospective Cohort	Journal of the American Geriatric Society	Medical-Surgical ICU	80+	81.7 (2.4)	51.00%	61.52%	1.29 (1.13)		.21	EQ-5D	44.4 months	Age-matched British population	M	187	142	-.24	.01
Eddleston[34]	2000	UK	Prospective Cohort	Critical Care Medicine	General Hospital ICU	65+		52.45%[a]					SF-36*	3 months	ICU survivors younger than 65 years old	M	39	97	-.21	.04
Ferrao[33]	2015	Portugal	Retrospective Cohort	Critical Care	Medical-Surgical ICU	66+[b]		26.00%					EQ-5D	27.6 months	ICU survivors younger than 65 years old	M	290	652	-.37	.01
Grace[31]	2007	Australia/NZ	Retrospective Cohort	Critical Care and Resuscitation	Mixed ICUs	60+		NR	60.00%			.28	EQ-5D	28 months	Retrospective patient ratings for one week before ICU	L	99	99	-.36	.02
Hofhuis[30]	2011	Netherlands	Prospective Cohort	Chest	Medical-Surgical ICU	80+[b]	83 (3.06)	46.90%	40.83%	5.35 (2.29)	25.48 (16.04)	.25	SF-36*	6 months	Age-matched Dutch population	M	49	49[c]	.26	.04
															Retrospective proxy ratings for four weeks before ICU		49	49	.01	.04

For peer review only

Jeitziner [29]	2015	Switzerland	Retrospective Cohort	Journal of Clinical Nursing	Medical-Surgical ICU	65+	68.72 (5.39)	73.00%		4.57 (5.81)		.29	SF-36 [*]	12 months	Age matched Swiss controls;	M	124	145	-.59	.02
															Retrospective patient ratings for one week before ICU		124	135	-.08	.01
Kaarola [28]	2006	Finland	Cross-Sectional	Critical Care Medicine	Medical-Surgical ICU	65+		75.00%	57.00%				EQ-5D	47 months	ICU survivors younger than 65 years old	M	299	800	-.67	.00
Levinson [26]	2016	Australia	Prospective Cohort	Internal Medicine Journal	Private ICU	80+	84.59 (NR)	58.00%[a]	21.45%	1.28 (NR)	12.91 (NR)		SF-36 [*]	24 months	Age and sex-matched Australian population	H	322	907	.04	.00
Merlani [25]	2007	Switzerland	Retrospective Cohort	Acta Anaesthesiologica Scandinavica	Surgical ICU	70+	78 (5)	52.00%	63.00%	3.00 (13.72)	22.50 (93.88)	.26	EQ-5D	24 months	Age-matched Swiss population	M	36	87	-.44	.04
Oeyen [24]	2007	Netherlands	Prospective Cohort	Minerva Medica	Medical-Surgical ICU	80+	83 (3)	60.00%[a]	49.60%	3.35 (2.26)	26.93 (27.11)	.26	EQ-5D	12 months	Retrospective patient or proxy ratings for one week before ICU	M	63	63	-.30	.03
Sacanella [23]	2011	Spain	Prospective Cohort	Critical Care	Medical ICU	65+	73.4 (5.5)	57.00%	48.70%	9.4 (10.20)		.27	EQ-5D	12 months	Retrospective patient or proxy ratings before feeling ill and requiring ICU	M	112	112	-.49	.02
Schroder [22]	2011	Denmark	Cohort (unspecified)	Danish Medical Bulletin	Mixed ICUs	75+		56.00%	53.85%				SF-36 [*]	12 months	Age-matched Danish population	L	36	229	-.03	.03
Sznajer [21]	2001	France	Prospective Cohort	Intensive Care Medicine	Mixed ICUs	65+ [b]		55.90%[a]					EQ-5D	6 months	ICU survivors younger than 65 years old	M	65	53	-.16	.03
Villa [19]	2016	Spain	Prospective Cohort	Journal of the American Geriatric Society	Medical-Surgical ICU	75+	80.8 (3.3)	50.00%	43.18%			.23	SF-36 [*]	12 months	Spanish population aged 75+	M	54	1363 [d]	-.15	.02

Table A.1. Full study characteristics for all effect sizes included in the meta-analysis

Abbreviations: Avg. Age (average age); ICU LoS (average length of stay in intensive care; days); HLoS (average length of stay in hospital; days); SD (standard deviation; sometimes estimated- see methods)

[a] Reported for study level only

[b] Combined elderly groups

[c] Assumed N based on matched sample

For peer review only - http://bmjopen.bmj.com/site/about/guidelines.xhtml

[d] Retrieved from López-García, E., Banegas, J. R., Graciani, A. P. R., Gutiérrez-Fisac, J. L., Alonso, J., & Rodríguez-Artalejo, F. (2003). Population-based reference values for the Spanish version of the SF-36 Health Survey in the elderly. Medicina clinica, 120(15), 568-573; a follow-up to the previous study, which was unavailable

Unless specified, we do not report data where it is not representative of at least 66.67% of the included sample.

1.2 Qualitative Only Studies

First Author	Year	Country	Study Design	Journal	Setting	Min Age	Participant No.	Avg. Age (SD)	% Male	ICU LoS (SD)	HLoS (SD)	Severity	Ineligible Measure	Follow up	Comparison
Garrouste-Orgeas	2006	France	Prospective Cohort	Intensive Care Medicine	Medical ICU	80+	28	84 (3.92)		12.6 (15.5)		.28	Nottingham Health Profile (NHP)	12 months	Age and sex-matched French population controls
Kleinpell	2002	USA	Retrospective Cohort	Research in Nursing and Health	Mixed ICUs	66+	128		42.00%	4.2 (6.17)	10.28 (9.63)	.18	Quality of Life Index (QLI)	4-6 months	ICU survivors aged between 45 and 64 years old
Tabah	2010	France	Prospective Cohort	Critical Care	Medical-Surgical ICU	80+	23	84 (3)	73.90%	5.72 (4.74)	18.08 (15.01)	.23	WHO-QOL-BREF	16 months	Age and sex-matched French population controls

Table A.2. Full study characteristics of all records that were only included in the qualitative synthesis.
Abbreviations: Avg. Age (average age); ICU LoS (average length of stay in intensive care; days); HLoS (average length of stay in hospital; days), SD (standard deviation; sometimes estimated- see methods)
[a] Reported for study level only

Unless specified, we do not report data where it is not representative of at least 66.67% of the included sample.

For peer review only

2. SENSITIVITY ANALYSES FOR INFLUENTIAL CASES

2.1 Overview of Outliers: Meta-Analysis

Comparison	k	First Author	Cook's Distance (Critical d)	Leave out Effect Size	Leave out P value	I^2 Change	Effect Size Change
Community	10	Pavoni	.98 (.40)	-2.09	.28	-30%	-1.94
Community	9	Jeitziner	.63 (.44)	-.08	.19	-31%	+.07

Table A.3. A summary of cases that fit our criteria as potentially influential. Excluded cases are highlighted in red.

First Author	Year	Country	Study Design	Journal	Setting	Min Age	Participant No.	Avg. Age (SD)	% Male	ICU LoS (SD)	HLoS (SD)	Severity	Mortality	Follow up	Comparison
Pavoni	2012	Italy	Prospective Cohort	Archives of Gerontology and Geriatrics	Mixed ICUs	80+	143	86.51 [a] (1.81)	26.74% [a]	5.27 [a] (5.80)	14.20 [a] (8.96)	.20 [a]	50% [a]	12 months	Age-matched Italian retirement community population

Table A.4. Study characteristics of the lone study excluded as an outlier.
Abbreviations: Avg. Age (average age); ICU LoS (average length of stay in intensive care; days); HLoS (average length of stay in hospital; days), SD (standard deviation; sometimes estimated- see methods)
[a] Reported for study level only

For peer review only

3. QUALITATIVE SYNTHESIS

3.1 Qualitative analysis procedure

Scale	Mental Health Subscale(s)	Physcial Health Subscale(s)	Additional Notes
EQ-5D	Anxiety/Depression	Mobility, Self-Care, Usual Activities, Pain/Discomfort	Raw scores scaled between 1-3
SF-36	Social Functioning, Role Emotional, Mental Health, Vitality	Physical Functioning, Bodily Pain, General Health, Role Physical	
NHP	Sleep, Emotional Reaction, Social Isolation	Pain, Energy, Physical Mobility	Reverse scoring
WHO-QOL-BREF	Psychological Health, Social Relationships	Overall perception of Health, Physical Health, Environment	
QLI	Socio-economic, Family, Psychological/Spiritual	Health and Functioning	Raw scores scaled between 0-30

Table A.5. Subscales used to estimate mental and physical health QoL within the qualitative synthesis.

For peer review only

4. SENSITIVITY ANALYSES FOR OBSERVED EFFECTS

4.1 Forest Plots

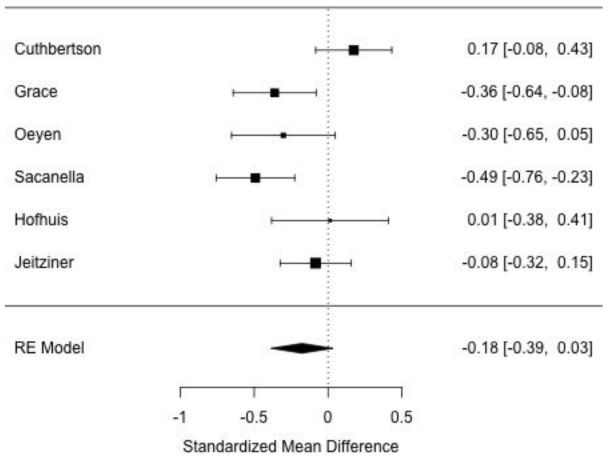

Figure A.1. Forest plot of differences in EQ-5D composite scores in elderly survivors, comparing pre-ICU and post-ICU scores.

peer review only

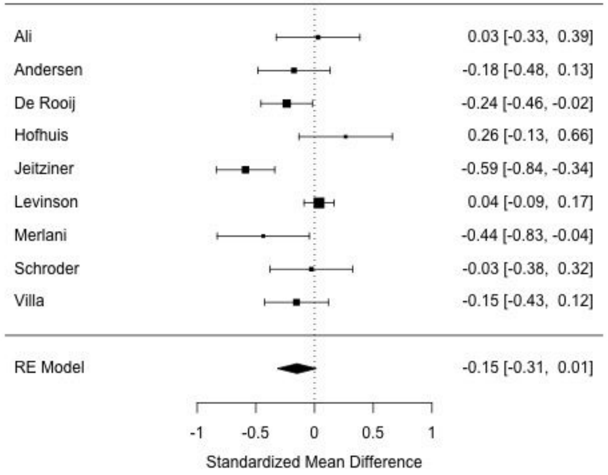

Figure A.2. Forest plot of differences in EQ-5D composite scores, comparing elderly ICU survivors at follow-up and age-matched community controls.

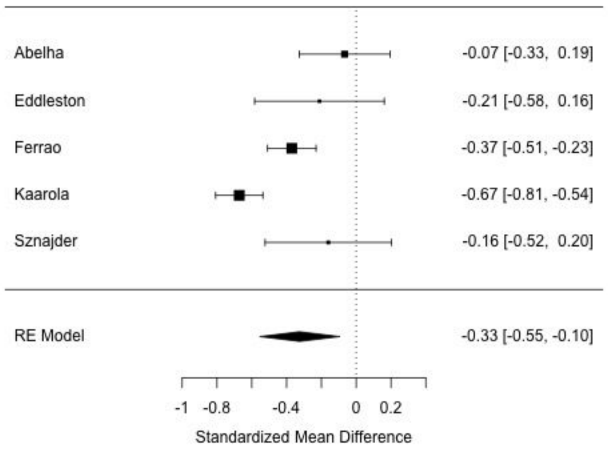

Figure A.3. Forest plot of differences in EQ-5D composite scores at follow-up, comparing elderly ICU survivors (aged 65+) and younger ICU survivors (aged under 65), both at follow-up.

peer review only

Appendix: Disparity or Discrimination?
A systematic review of socio-demographic associations of insight

4.2 Funnel Plots

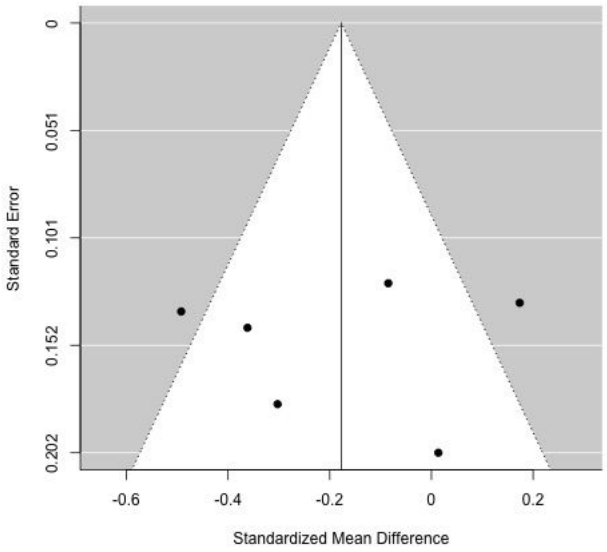

Figure A.4. Funnel plot of studies that investigated differences in EQ-5D composite scores in elderly survivors, comparing pre-ICU and post-ICU scores.

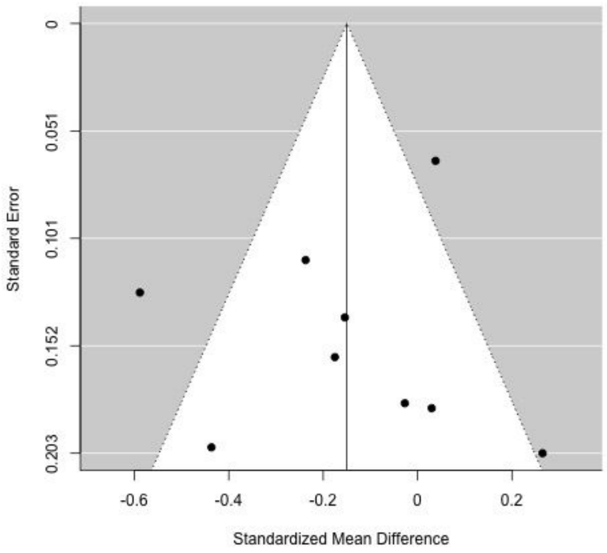

Figure A.5. Funnel plot of studies that compared EQ-5D scores in elderly ICU survivors at follow-up and age-matched community controls.

For peer review only - http://bmjopen.bmj.com/site/about/guidelines.xhtml

Appendix: Disparity or Discrimination?
A systematic review of socio-demographic associations of insight

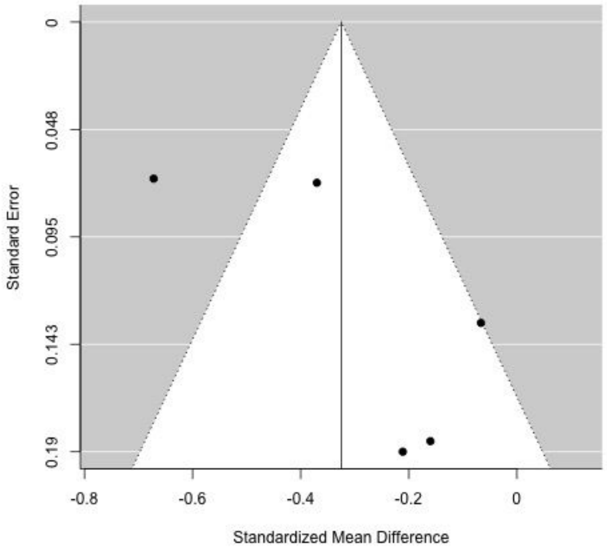

Figure A.6. Funnel plot of studies that compared EQ-5D scores in elderly ICU survivors (aged 65+) and younger ICU survivors (aged under 65), both at follow-up.

4.3 Cook's Distance Plots

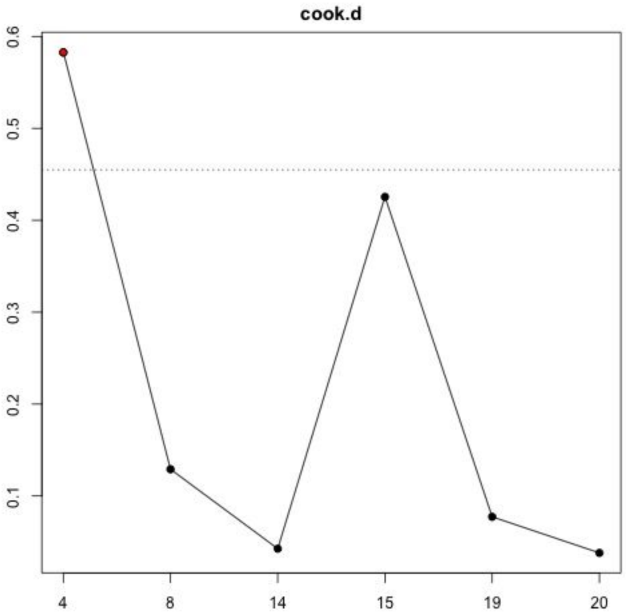

Figure A.7. Cook's distance plot of studies that investigated differences in EQ-5D composite scores in elderly survivors, comparing pre-ICU and post-ICU scores.

For peer review only - http://bmjopen.bmj.com/site/about/guidelines.xhtml

Appendix: Disparity or Discrimination?
A systematic review of socio-demographic associations of insight

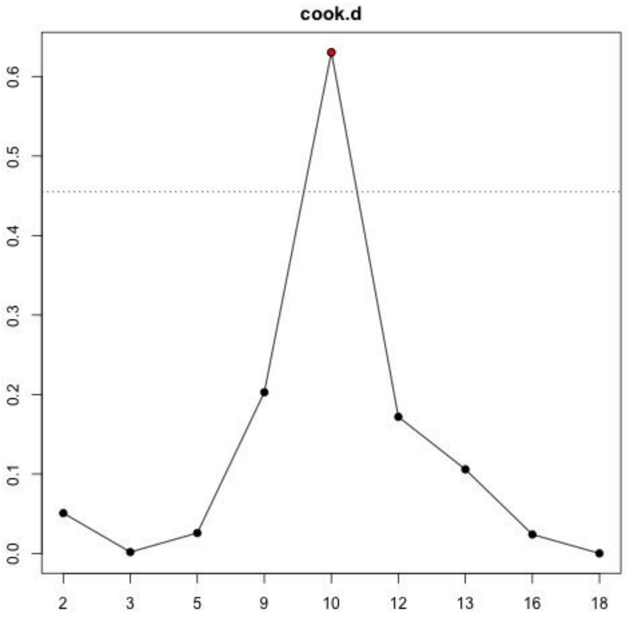

Figure A.8. Cook's distance plot of studies that compared EQ-5D scores in elderly ICU survivors at follow-up and age-matched community controls.

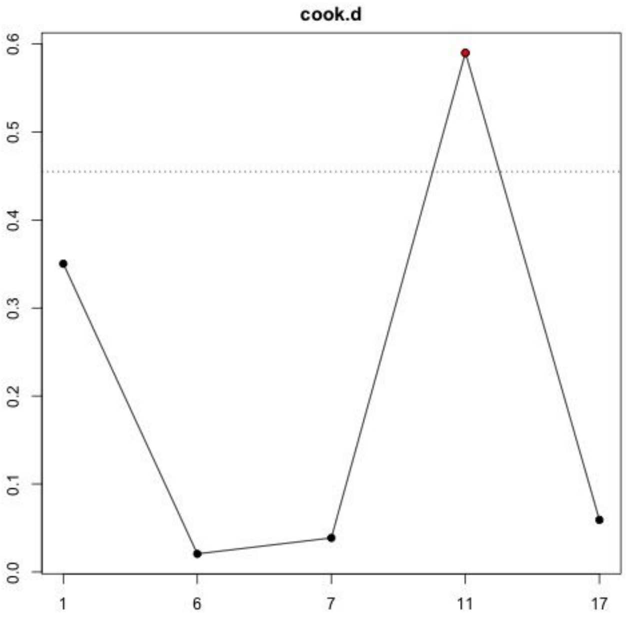

Figure A.9. Cook's distance plot of studies that compared EQ-5D scores in elderly ICU survivors (aged 65+) and younger ICU survivors (aged under 65), both at follow-up.

For peer review only - http://bmjopen.bmj.com/site/about/guidelines.xhtml

Appendix: Disparity or Discrimination?
A systematic review of socio-demographic associations of insight

5. REVIEW PROTOCOL

5.1 ICU Review Protocol

Included	Excluded
**Design**	
Case note analyses (longitudinal)<br>Case control<br>Retrospective cohort<br>Prospective cohort<br>Unpublished dissertations of the above	Qualitative only studies<br>Systematic review or meta-analysis (categorise in separate folder)<br>Narrative review<br>Non-English language (if translation can't be found)<br>Commentaries<br>Case studies<br>Small N samples (<20 eligible participants)<br>Conference abstracts<br>Brief reports<br>Books
**Population**	
Patients aged 60+ who have undergone ICU<br>Medical, Surgical or Mixed ICU settings	<20 eligible patients aged 60+<br>Veteran, trauma or emergency care setting<br>Non-OECD country<br>Non-human participants<br>Palliative care<br>Non-ICU patients
**Focus**	
Patients aged 60+ who have undergone ICU	Neurological ICU patients only<br>Cardiosurgical ICU patients only
Follow up of at least 3 months	No follow up/Follow up less than three months
At least one of the following comparison groups:<br>• Age-matched community controls<br>• Scores taken before ICU<br>• Younger ICU patients	No comparison group
QoL at follow up measured by patients (carers may help but cannot do assessment on their own)	QoL at follow up all measured by proxy (ie. doctors or carers)
**Data/Outcomes**	
Validated QoL measure (EQ-5D, SF-36, NHP, WHOQOLBREF, QLI or variants of these)	Non-validated QoL measure only (eg. a simple question of whether QoL improved)
QoL summary score reported in paper for both groups, or:<br>• Subscores can be used to calculate summary scores<br>• Study references data for age-matched control that is fully reported elsewhere	No eligible data on QoL (or insufficient data to calculate summary scores)<br>QoL not reported for both groups (regression analyses do not count)

For peer review only

Appendix: Disparity or Discrimination?
A systematic review of socio-demographic associations of insight

6. REVIEW SEARCH TERMS

6.1 MEDLINE

(("intensive care"[title/abstract] OR "critical care"[title/abstract] OR "critical illness"[title/abstract] OR "Respiratory Distress Syndrome"[title/abstract] OR "Sepsis"[title/abstract] OR intensive care[MeSH Terms] OR critical care[MeSH Terms] OR "critical illness"[MeSH Terms] OR "Sepsis"[MeSH Terms]))

AND (("elderly"[title/abstract] OR "older adult*"[title/abstract] OR "geriatr*"[title/abstract] OR "dement*"[title/abstract] OR "Alzheimer*"[title/abstract] OR "parkinson's disease"[title/abstract] OR elderly [MeSH Terms] OR older adult*[MeSH Terms] OR geriatr*[MeSH Terms] OR dement*[MeSH Terms] OR septugenaria*[All Fields] OR octogenaria*[All Fields] OR nonagenaria*[All Fields] OR "over 5*"[title/abstract] OR "over 6*"[title/abstract] OR "over 7*"[title/abstract] OR "over 8*"[title/abstract] OR "over 9*"[title/abstract] OR "over 5*"[title/abstract] OR "over 6*"[title/abstract] OR "over 7*"[title/abstract] OR "over 8*"[title/abstract] OR "over 9*"[title/abstract]))

AND (("quality of life"[title/abstract] OR "EuroQol*"[All Fields] OR "Nottingham Health Profile"[All Fields] OR "NHP*"[All Fields] OR "SF-36"[All Fields] OR "RAND-36*"[All Fields]))

Filters: English Language, Humans, 01/01/2000 to 23/04/2020

6.2 Cochrane Database for Systematic Reviews & Cochrane Controlled Register of Trials (CENTRAL)

#1 ("intensive care" OR "critical care" OR "critical illness" OR "Respiratory Distress Syndrome" OR "Sepsis"):ti,ab,kw
#2 ("elderly" OR "older adult*" OR "geriatr*" OR "dement*" OR "Alzheimer*" OR "parkinson's disease"):ti,ab,kw
#3 (critical care OR critical illness OR Sepsis)
#4 (Aged OR geriatrics OR dementia)
#5 ("quality of life")
#6 ("EuroQol" OR "Nottingham Health Profile" OR "NHP" OR "SF-36" OR "RAND-36")
#7 MeSH descriptor: [Aged]
#8 MeSH descriptor: [Geriatrics]
#9 MeSH descriptor: [Dementia]
#10 MeSH descriptor: [Critical Care]
#11 MeSH descriptor: [Critical Illness]
#12 MeSH descriptor: [Sepsis]
#13 #1 OR #3 OR #10 OR #11 OR #12
#14 #2 OR #4 OR #7 OR #8 OR #9
#15 #5 AND #6
#16 #13 AND #14 AND #15= 124 (78 reviews, 36 trials).

6.3 Web of Science

Indexes = SCI-EXPANDED, SSCI, CPCI-S, CPCI-SHH, ESCI. **LANGUAGE** = English, **DOCUMENT TYPES** = (Article OR Abstract of Published Item), Timespan = All years (2000-2020)

#1 ALL=("intensive care" OR "critical care" OR "critical illness" OR "Respiratory Distress Syndrome" OR "Sepsis" OR "ICU")
#2 ALL=("elderly" OR "older adult*" OR "geriatr*" OR "dement*" OR "Alzheimer*" OR "parkinson's disease")
#3 ALL= ("quality of life" OR "EuroQol" OR "Nottingham Health Profile" OR "NHP" OR "SF-36" OR "RAND-36")
#4 #1 AND #2 AND #3
#5 #4 AND **LANGUAGE**: (English) AND **DOCUMENT TYPES**: (Article OR Abstract of Published Item) AND **Timespan**= 2000-2020

For peer review only - http://bmjopen.bmj.com/site/about/guidelines.xhtml

Appendix: Disparity or Discrimination?
A systematic review of socio-demographic associations of insight

6.4 EMBASE (& EMBASE Classic)

Dates: 2000-2020, Limits: Human participants only, English language, Articles only

#1 All Field: "intensive care" or "critical care" or "critical illness" or "Respiratory Distress Syndrome" or Sepsis or "ICU"
#2 Text Word: elderly or "older adult*" or "geriatr*" or "dement*" or "Alzheimer*" or "parkinson*"
#3 All Field: "quality of life" or EuroQol or Nottingham Health Profile or NHP or SF-36 OR RAND-36

6.5 CINAHL

Limits: English language only, Human participants, All adult, Peer-reviewed, Jan 2000 – April 2020

#1 TX: "intensive care" or "critical care" or "critical illness" or "Respiratory Distress Syndrome" or Sepsis or "ICU"
#2: SU: "Intensive Care Units" or "Intensive Care Units or Neonatal" or "Critical Care Nursing" or "Respiratory Distress Syndrome" or Acute or "Neonatal Intensive Care Nursing" or "Critical Care or Critical Path" or "Canadian Association of Critical Care Nurses" or "British Association of Critical Care Nurses" or "ventilator patients"
#3: TX: elderly or "older adult*" or "geriatr*" or "dement*" or "Alzheimer*" or "parkinson*"
#4: SU: "Older Adult Care (Saba CCC)" or "Frail Elderly" or "elderly patients" or "ventilator patients"
#5: TX: "quality of life" or EuroQol or "Nottingham Health Profile" or NHP or SF-36 OR RAND-36
#6: (S1 OR S2) AND (S3 OR S4) AND S5

6.6 PsycINFO

Limits: Date filter (2000-2020), English language, Human participants, Peer Reviewed Journal

#1 All Fields: "intensive care" or "critical care" or "critical illness" or "Respiratory Distress Syndrome" or Sepsis or "ICU"
#2 Text Word: elderly or "older adult*" or "geriatr*" or "dement*" or "Alzheimer*" or "parkinson*"
#3 All Fields: "quality of life" or EuroQol or Nottingham Health Profile or NHP or SF-36 OR RAND-36

For peer review only

PRISMA 2009 Checklist

Section/topic	#	Checklist item	Reported on page #
**TITLE**			
Title	1	Identify the report as a systematic review, meta-analysis, or both.	1
**ABSTRACT**			
Structured summary	2	Provide a structured summary including, as applicable: background; objectives; data sources; study eligibility criteria, participants, and interventions; study appraisal and synthesis methods; results; limitations; conclusions and implications of key findings; systematic review registration number.	1
**INTRODUCTION**			
Rationale	3	Describe the rationale for the review in the context of what is already known.	3
Objectives	4	Provide an explicit statement of questions being addressed with reference to participants, interventions, comparisons, outcomes, and study design (PICOS).	3
**METHODS**			
Protocol and registration	5	Indicate if a review protocol exists, if and where it can be accessed (e.g., Web address), and, if available, provide registration information including registration number.	1 & 4
Eligibility criteria	6	Specify study characteristics (e.g., PICOS, length of follow-up) and report characteristics (e.g., years considered, language, publication status) used as criteria for eligibility, giving rationale.	4, 8 & Appendix
Information sources	7	Describe all information sources (e.g., databases with dates of coverage, contact with study authors to identify additional studies) in the search and date last searched.	4 & Appendix
Search	8	Present full electronic search strategy for at least one database, including any limits used, such that it could be repeated.	4 & Appendix
Study selection	9	State the process for selecting studies (i.e., screening, eligibility, included in systematic review, and, if applicable, included in the meta-analysis).	4-6 & Appendix
Data collection process	10	Describe method of data extraction from reports (e.g., piloted forms, independently, in duplicate) and any processes for obtaining and confirming data from investigators.	4-5 & Appendix
Data items	11	List and define all variables for which data were sought (e.g., PICOS, funding sources) and any assumptions and simplifications made.	4 & Appendix
Risk of bias in individual studies	12	Describe methods used for assessing risk of bias of individual studies (including specification of whether this was done at the study or outcome level), and how this information is to be used in any data synthesis.	5, 9, 10 and Appendix
Summary measures	13	State the principal summary measures (e.g., risk ratio, difference in means).	5

For peer review only - http://bmjopen.bmj.com/site/about/guidelines.xhtml

PRISMA 2009 Checklist

Synthesis of results	14	Describe the methods of handling data and combining results of studies, if done, including measures of consistency (e.g., I^2) for each meta-analysis.	5

Section/topic	#	Checklist item	Reported on page #
Risk of bias across studies	15	Specify any assessment of risk of bias that may affect the cumulative evidence (e.g., publication bias, selective reporting within studies).	5, 9, 10 and Appendix
Additional analyses	16	Describe methods of additional analyses (e.g., sensitivity or subgroup analyses, meta-regression), if done, indicating which were pre-specified.	5
**RESULTS**			
Study selection	17	Give numbers of studies screened, assessed for eligibility, and included in the review, with reasons for exclusions at each stage, ideally with a flow diagram.	6
Study characteristics	18	For each study, present characteristics for which data were extracted (e.g., study size, PICOS, follow-up period) and provide the citations.	8 & Appendix
Risk of bias within studies	19	Present data on risk of bias of each study and, if available, any outcome level assessment (see item 12).	8-10 & Appendix
Results of individual studies	20	For all outcomes considered (benefits or harms), present, for each study: (a) simple summary data for each intervention group (b) effect estimates and confidence intervals, ideally with a forest plot.	9 & Appendix
Synthesis of results	21	Present results of each meta-analysis done, including confidence intervals and measures of consistency.	9 & Appendix
Risk of bias across studies	22	Present results of any assessment of risk of bias across studies (see Item 15).	9-10 & Appendix
Additional analysis	23	Give results of additional analyses, if done (e.g., sensitivity or subgroup analyses, meta-regression [see Item 16]).	9-10 & Appendix
**DISCUSSION**			
Summary of evidence	24	Summarize the main findings including the strength of evidence for each main outcome; consider their relevance to key groups (e.g., healthcare providers, users, and policy makers).	12
Limitations	25	Discuss limitations at study and outcome level (e.g., risk of bias), and at review-level (e.g., incomplete retrieval of identified research, reporting bias).	13-14
Conclusions	26	Provide a general interpretation of the results in the context of other evidence, and implications for future research.	13, 15

For peer review only - http://bmjopen.bmj.com/site/about/guidelines.xhtml

PRISMA 2009 Checklist

FUNDING			
Funding	27	Describe sources of funding for the systematic review and other support (e.g., supply of data); role of funders for the systematic review.	15

From: Moher D, Liberati A, Tetzlaff J, Altman DG, The PRISMA Group (2009). Preferred Reporting Items for Systematic Reviews and Meta-Analyses: The PRISMA Statement. PLoS Med 6(7): e1000097. doi:10.1371/journal.pmed1000097

For more information, visit: **www.prisma-statement.org**.

For peer review only

For peer review only - http://bmjopen.bmj.com/site/about/guidelines.xhtml

Quality of Life in elderly ICU survivors before the COVID-19 pandemic: A Systematic Review and Meta-Analysis of Cohort Studies.

Journal:	*BMJ Open*
Manuscript ID	bmjopen-2020-045086.R1
Article Type:	Original research
Date Submitted by the Author:	31-Mar-2021
Complete List of Authors:	Ariyo, Kevin; King's College London Department of Psychological Medicine<br>Canestrini, Sergio; King's College Hospital NHS Foundation Trust, Critical Care<br>David, Anthony; UCL, Institute of Mental Health<br>Ruck Keene, Alex; King's College London Department of Psychological Medicine; King's College London Dickson Poon School of Law<br>Wolfrum, Sebastian; University Hospital Schleswig Holstein, Medical Clinic II, Cardiology/Angiology/Intensive Care Medicine; University Hospital Schleswig Holstein, Department of Emergency Medicine<br>Owen, Gareth; King's College London Department of Psychological Medicine
**Primary Subject Heading**:	Intensive care
Secondary Subject Heading:	Epidemiology, Ethics, Health policy, Mental health, Public health
Keywords:	COVID-19, INTENSIVE & CRITICAL CARE, STATISTICS & RESEARCH METHODS, ETHICS (see Medical Ethics), Rationing < HEALTH SERVICES ADMINISTRATION & MANAGEMENT

SCHOLARONE™
Manuscripts

I, the Submitting Author has the right to grant and does grant on behalf of all authors of the Work (as defined in the below author licence), an exclusive licence and/or a non-exclusive licence for contributions from authors who are: i) UK Crown employees; ii) where BMJ has agreed a CC-BY licence shall apply, and/or iii) in accordance with the terms applicable for US Federal Government officers or employees acting as part of their official duties; on a worldwide, perpetual, irrevocable, royalty-free basis to BMJ Publishing Group Ltd ("BMJ") its licensees and where the relevant Journal is co-owned by BMJ to the co-owners of the Journal, to publish the Work in this journal and any other BMJ products and to exploit all rights, as set out in our licence.

The Submitting Author accepts and understands that any supply made under these terms is made by BMJ to the Submitting Author unless you are acting as an employee on behalf of your employer or a postgraduate student of an affiliated institution which is paying any applicable article publishing charge ("APC") for Open Access articles. Where the Submitting Author wishes to make the Work available on an Open Access basis (and intends to pay the relevant APC), the terms of reuse of such Open Access shall be governed by a Creative Commons licence – details of these licences and which Creative Commons licence will apply to this Work are set out in our licence referred to above.

Other than as permitted in any relevant BMJ Author's Self Archiving Policies, I confirm this Work has not been accepted for publication elsewhere, is not being considered for publication elsewhere and does not duplicate material already published. I confirm all authors consent to publication of this Work and authorise the granting of this licence.

For peer review only

Quality of Life in elderly ICU survivors before the COVID-19 pandemic: A Systematic Review and Meta-Analysis of Cohort Studies.

ABSTRACT

Kevin Ariyo [1], Sergio Canestrini [2,3], Anthony S. David [4], Alex Ruck Keene [1,5], Sebastian Wolfrum [6,7], Gareth S. Owen [1].

1. Department of Psychological Medicine, Institute of Psychiatry, Psychology and Neuroscience, King's College London, UK
2. Department of Critical Care, King's College Hospital, Denmark Hill, London, UK
3. Centre for Social Ethics and Policy, Department of Law, School of Social Science, University of Manchester, UK
4. UCL Institute of Mental Health, Division of Psychiatry, University College London, UK
5. Dickson Poon School of Law, King's College London, UK
6. Medical Clinic II, Cardiology/Angiology/Intensive Care Medicine, University Hospital Schleswig-Holstein, Germany
7. Department of Emergency Medicine, University Hospital Schleswig-Holstein, Germany

OBJECTIVES

The influence of age upon intensive care unit (ICU) decision-making is complex and it is unclear if it is based on expected subjective or objective patient outcomes. To address recent concerns over age-based ICU decision-making we explored patient-assessed quality of life (QoL) in ICU survivors before the COVID-19 pandemic.

DESIGN

A systematic review of cohort studies published between January 2000 to April 2020, of elderly patients admitted to ICUs.

PRIMARY AND SECONDARY OUTCOME MEASURES

We extracted data on self-reported QoL (EQ-5D composite score), demographic and clinical variables. Using a random-effects meta-analysis, we then compared QoL scores at follow-up to scores either before admission, age-matched population controls or younger ICU survivors. We conducted sensitivity analyses to study heterogeneity and bias, and a qualitative synthesis of subscores.

RESULTS

We identified 2536 studies and included 22 for qualitative synthesis and 19 for meta-analysis (N= 2442 elderly survivors). Elderly survivors' QoL was not significantly different between one month before ICU and follow-up. Elderly survivors' QoL was significantly worse than younger ICU survivors, with a small-to-medium effect size (d= .35 [-.53, -.16]). Their QoL was also marginally significantly worse than age-matched community controls, with a small effect size (d= .21 [-.43, .00]). Mortality rates and length of follow up partly explained heterogeneity. Reductions in QoL seemed primarily due to physical health, rather than mental health items.

CONCLUSIONS

The results suggest that the proportionality of age as a determinant of ICU resource allocation should be kept under close review and that subjective QoL outcomes should inform person-centred decision making in elderly ICU patients.

For peer review only - http://bmjopen.bmj.com/site/about/guidelines.xhtml

Quality of Life in elderly ICU survivors before the COVID-19 pandemic:
A Systematic Review and Meta-Analysis of Cohort Studies.

1

Strengths and limitations of this study

- To our knowledge, this is the first systematic review and meta-analysis to explore quality of life outcomes in elderly ICU survivors, and to explore sources of variation between these studies.
- To ensure consistency and policy relevance, we only included one type of measure within the meta-analysis (EQ-5D).
- With our large sample, we could estimate the population QoL with reasonable precision, as evidenced by narrow confidence intervals.
- Wide prediction intervals suggest that our results should not be used to make individual-level predictions.
- Our sample had a mixture of conditions, and because data was reported inconsistently and often at study-level, it is difficult to generalise to specific clinical groups, including COVID-19 patients.

For peer review only

For peer review only - http://bmjopen.bmj.com/site/about/guidelines.xhtml

Quality of Life in elderly ICU survivors before the COVID-19 pandemic:
A Systematic Review and Meta-Analysis of Cohort Studies.

INTRODUCTION

The influence that age should have upon intensive care decision making has been debated across policy and clinical practice [1 2]. Age associates (inversely) with the probability of intensive care unit (ICU) survival and length of life after ICU [3 4], outcomes generally considered to be relevant to resource allocation [2]. However, age is also a protected characteristic in several jurisdictions, and in England and Wales, resource allocation based on age must be a “proportionate means of achieving a legitimate aim” , if it is not to be contrary to the Equality Act (2010) .

For elderly patients for whom admission to ICU is clinically appropriate, an important part of person-centred decision-making is for them, or their families, to be given information about the likely outcome of admission. Patients may seek to integrate survival and biomedical outcomes with subjective outcomes, including quality of life (QoL). Subjective QoL in elderly ICU survivors has been studied less frequently than these objective measures [3 5]. This is notable given that subjective QoL (via Quality-Adjusted Life Years, or QALYs) is very influential in clinical resource allocation (e.g. NICE). Person-centred decision making requires consideration of patient experience since physician-rated quality of life is not always well correlated with patient-rated quality of life .

We considered a rapid review to be urgent because age is a strong risk factor for severe COVID-19 infection [6] and severe COVID-19 has placed considerable pressure on ICU resource allocation. [7] and is likely to do so in the future. Additionally, some have expressed concerns that elderly adults may be disproportionately less likely to receive ICU [1 2 8-10]. It is therefore important older persons’ subjective outcomes are better understood.

We conducted a meta-analysis on patient reported QoL in elderly adults undergoing ICU. Following a systematic review, we addressed three questions:

1) At follow up, do elderly ICU survivors have better/worse QoL compared to their scores before ICU?
2) At follow up, do elderly ICU survivors have better/worse QoL than age-matched community controls?
3) At follow up, do elderly ICU survivors have better/worse QoL than ICU survivors aged under 65?

Determining the effect of illness and ICU on QoL is complicated because QoL is itself influenced by many variables [11] and some are non-clinical. These influences are too complex to resolve completely, but where possible, we sought to model relevant variables (illness severity, ICU length of stay and mortality rate) as predictors of QoL in elderly ICU survivors at follow up, compared to controls.

For peer review only - http://bmjopen.bmj.com/site/about/guidelines.xhtml

Quality of Life in elderly ICU survivors before the COVID-19 pandemic: A Systematic Review and Meta-Analysis of Cohort Studies.

METHODS

SEARCH STRATEGY

We searched for English-language journal articles, published between January 2000 and April 2020. Six online bibliographic databases were used: CENTRAL, CINAHL, Cochrane Library, EMBASE, MEDLINE and PsycINFO. Our search followed pre-published PROSPERO protocol (ID: CRD42020181181).

The search terms focused on intensive care, elderly adults and QoL. We supplemented this with a forward citations and reference list search based on the eligible articles as well as consultation with experts.

PATIENT AND PUBLIC INVOLVEMENT

No patient or public advisers were involved in this project.

SELECTION CRITERIA

We undertook study selection using EndNote X9 using a standardised CRIB sheet. See Figure 1 for a full overview.

At the title and abstract level, we identified potentially eligible studies that took place in an ICU and referred to either QoL life or elderly adults. Full texts were eligible if a) all participants underwent ICU; b) there were at least 20 elderly patients and controls; c) scores from a validated QoL scale were reported, for a group aged at least 60+, with at least 3 months follow up review; d) the follow up QoL scores were derived from the patient, rather than a professional ; and e) the study reported QoL scores from the same scale for either the same patients before the ICU admission, age-matched community controls or ICU survivors aged under 65.

Where we could not include potentially eligible studies, due to poor reporting, we contacted study authors for unpublished data. We also considered whether to include studies that focused only on patients admitted to cardiac or neuro-surgical ICUs, given the effects of the diagnostic heterogeneity that characterises the reference population of the studies included in our review (general ICU patients with various conditions). However, none of these studies met the other inclusion criteria.

K.A led the study selection at all stages and a post-doctoral research assistant conducted reliability checks for 50% of full text articles. We found nearly perfect inter-rater agreement, as measured by Cohen's kappa (k= .86) [12]. Queries were resolved by G.O.

DATA EXTRACTION

One reviewer (K.A) extracted relevant data from all eligible studies, recording this on a standardised spreadsheet. M.K. independently extracted data from 10% of eligible studies, to evaluate consistency. The primary outcome was the QoL composite scores. Secondary variables included demographics, QoL subscale scores, mortality (from ICU to follow up), illness severity (APACHE-II or SAPS-II), length of ICU stay, length of hospital stay, and average follow up time. When one dataset was used for multiple studies, we included the study with the clearest data reporting.

For peer review only - http://bmjopen.bmj.com/site/about/guidelines.xhtml

Quality of Life in elderly ICU survivors before the COVID-19 pandemic: A Systematic Review and Meta-Analysis of Cohort Studies.

To ensure consistency, we included only composite scores from the EuroQoL health related quality of life instrument (EQ-5D) within the meta-analysis. Where possible, we also converted the eight SF-36 subscales to an EQ-5D index score, using an established mapping algorithm. [13] The remaining studies were included within the qualitative synthesis only.

DATA ANALYSIS

We explored the effect of age on EQ-5D composite scores using random effects meta-analyses. KA conducted the analysis using R Statistics. We used the Restricted Maximum Likelihood (REML) method to calculate the effect sizes (Cohen's d), which were weighted by the inverse of the sampling variance: meaning that studies with higher variance contributed less to the summary effect size. We interpreted these effect sizes using conventional criteria as a guide (0.2 = small; 0.5 = medium; 0.8 = large) [14]. We then conducted sensitivity analyses for each meta-analysis to assess risk of bias at the study level, including heterogeneity (e.g. I^2 statistic), influential studies (e.g. Cook's distance), and publication bias (funnel plots and Egger's test).

To investigate the remaining heterogeneity, we then conducted two secondary analyses: a moderator analysis to explore variation within a specific predictor, and a random-effects meta-regression to explore relationships between multiple predictors.

We used several strategies to handle missing data. When the study only reported median values and interquartile ranges, we estimated the mean and standard deviation using conventional formulae [15 16]. When neither the standard deviation nor interquartile range was reported, we estimated the standard deviation using prognostic imputation [17]. This calculates the average of observed variances to estimate the missing standard deviation values. We excluded studies with missing data if these methods were inapplicable.

One reviewer (K.A) assessed the methodological rigour of the included studies using an 11-item quality checklist (three irrelevant items were excluded) [18]. The criteria were scored as either 2 (complete fulfilment), 1 (partial fulfilment) or 0 (not fulfilled). We then calculated a total score for each study and rated them as either high quality (17/22 or higher), moderate quality (between 10/22 and 16/22) or low quality (9/22 or lower). Queries were resolved through discussion with G.O and S.C.

For the qualitative synthesis, we defined a set of criteria for each measure to allocate subscores to either 'mental health' or 'physical health' categories. We then calculated a crude average for subscales within these two categories and weighted them on a scale of 1-100 (0= minimum QoL; 100 = maximum QoL). As this approach is subjective, we present these findings only as a qualitative supplement.

This study follows methodological guidance from PRISMA (see appendix).

<Figure 1>

Figure 1. A PRISMA flow diagram that outlines the study selection process.

For peer review only - http://bmjopen.bmj.com/site/about/guidelines.xhtml

Quality of Life in elderly ICU survivors before the COVID-19 pandemic:
A Systematic Review and Meta-Analysis of Cohort Studies.

RESULTS

DESCRIPTIVE STATISTICS

After screening duplicates, the database search revealed 2536 records for title and abstract screening. From these, we reviewed 421 potentially relevant full text articles for eligibility. 18 of these studies met the full criteria and were included in the initial meta-analysis. A further two studies were deemed eligible following a forward citation search and contact with study authors. This led to a total of 20 studies included in the initial meta-analysis (n= 2585 elderly adults). Eleven of these studies reported age characteristics for the elderly patients (M= 79.04), while the others reported the minimum age only.

Most of the studies included both medical and surgical ICU patients (17 studies). The remaining studies focused on surgical (two studies) or medical (one study) patients only. A full breakdown of reasons for admissions is available in the appendix.

Three types of outcome were included in the meta-analysis. These results compared QoL at follow up to either pre-ICU scores (six studies), age-matched community controls (ten studies), or younger survivors of ICU (six studies). We provide a full summary in Table 1.

For the qualitative analysis, we identified three further studies. four different measurement scales were reported: the EuroQoL EQ-5D health related quality of life instrument (EQ-5D utility index or visual analogue scale; eleven studies), the short form medical outcome questionnaire (SF-36; eight studies), the Nottingham health profile (NHP; one study), the quality of life index (QLI; one study) and the World Health Organisation quality of life instruments (WHO-QOL-BREF; one study). SF-36 scores were converted to EQ-5D index scores for the meta-analysis, while the other measures were excluded (see methods).

For peer review only - http://bmjopen.bmj.com/site/about/guidelines.xhtml

Quality of Life in elderly ICU survivors before the COVID-19 pandemic: A Systematic Review and Meta-Analysis of Cohort Studies.

First Author	Year	Country	N	Min Age	% Male	Follow-up (avg. months)	ICU LoS (days)	Mortality	Severity (scaled avg.)	Raw Measure	Comparison	Quality
Abelha	2007	Portugal	114	65+	61.00%	6		28.00%		SF-36 *	ICU survivors younger than 65 years old	M
Ali	2018	Australia	32	65+	80.00% [a]	12	5		.24	EQ-5D	Age-matched South Australian controls	H
Andersen	2015	Norway	53	80+	69.00%	40.8	1.9	81.52%	.27	EQ-5D	Age and sex-matched Norwegian population	M
Cuthbertson	2005	UK	62	65+	59.00% [a]	12		33.00%		SF-36 *	ICU survivors younger than 65 years old	M
De Rooij	2008	Netherlands	187	80+	51.00%	44.4	1.29	61.52%	.21	EQ-5D	Age-matched British population	M
Eddleston	2000	UK	39	65+	52.45% [a]	3				SF-36 *	ICU survivors younger than 65 years old	M
Ferrao	2015	Portugal	290	66+ [b]	26.00%	27.6				EQ-5D	ICU survivors younger than 65 years old	M
Grace	2007	Australia/NZ	99	60+	NR	28		60.00%	.28	EQ-5D	Retrospective patient ratings for one week before ICU	L
Hofhuis	2011	Netherlands	49	80+ [b]	46.90%	6	5.35	40.83%	.25	SF-36 *	Age-matched Dutch population	M
Hofhuis	2011	Netherlands	49	80+ [b]	46.90%	6	5.35	40.83%	.25	SF-36 *	Retrospective proxy ratings for four weeks before ICU	M
Honselmann [c]	2015	Germany	352	65+	53.40%	12	2.58	43.36%		EQ-5D	ICU survivors younger than 65 years old	
Honselmann [c]	2015	Germany	291	65+	53.61%	12	2.34	43.36%		EQ-5D	Age-matched German controls	
Jeitziner	2015	Switzerland	124	65+	73.00%	12	4.57		.29	SF-36 *	Age matched Swiss controls;	M
Jeitziner	2015	Switzerland	124	65+	73.00%	12	4.57		.29	SF-36 *	Retrospective patient ratings for one week before ICU	
Kaarola	2006	Finland	299	65+	75.00%	47		57.00%		EQ-5D	ICU survivors younger than 65 years old	M
Levinson	2016	Australia	322	80+	58.00% [a]	24	1.28	21.45%		SF-36 *	Age and sex-matched Australian population	H
Merlani	2007	Switzerland	36	70+	52.00%	24	3.00	63.00%	.26	EQ-5D	Age-matched Swiss population	M
Oeyen	2007	Netherlands	63	80+	60.00% [a]	12		49.60%	.26	EQ-5D	Retrospective patient or proxy ratings for one week before ICU	M
Sacanella	2011	Spain	112	65+	57.00%	12	3.35	48.70%	.27	EQ-5D	Retrospective patient or proxy ratings before feeling ill and requiring ICU	M
Schroder	2011	Denmark	36	75+	56.00%	12	9.4	53.85%		SF-36 *	Age-matched Danish population	L
Sznajer	2001	France	65	65+ [b]	55.90% [a]	6				EQ-5D	ICU survivors younger than 65 years old	M
Villa	2016	Spain	54	75+	50.00%	12		43.18%	.23	SF-36 *	Spanish population aged 75+	M
Weighted avg.			*128.53*	*69.50*	*55.74%*	*22.98*	*3.02*	*44.92%*	*.26*			
Range			*23-352*	*60-80*	*26-80%*	*3-100.8*	*1.28-9.4*	*21.45-81.52%*	*.12-.34*			

Table 1. The main characteristics of the studies and the relevant data included in the meta-analyses.

[a] Reported for study level only, so not included in meta-analysis

[b] Combined elderly groups

[c] We analysed some unpublished data from Honselmann et al, therefore we have presented descriptives for the full dataset only.

* Converted to EQ-5D composite score

Abbreviations: ICU (intensive care unit); LoS (length of stay); H = High quality; M= Moderate quality; L= Low quality. See above for measures.

Unless specified, we do not report data where it is not representative of at least 66.67% of the included sample.

For peer review only - http://bmjopen.bmj.com/site/about/guidelines.xhtml

For peer review only

Quality of Life in elderly ICU survivors before the COVID-19 pandemic:
A Systematic Review and Meta-Analysis of Cohort Studies.

META ANALYSES

Comparison	k	Cohen d	95% CI	95% PI	P	I^2
Pre-ICU scores	6	-.18	-.39, .03	-.65, .29	.097	67.91%
Community	10	-.22	-.43, .00	-.88, .45	.053	87.88%
Under 65s	6	-.35	-.53, -.16	-.83, .18	.000	81.93%

Table 2. A summary of effect sizes, confidence intervals, prediction intervals, significance and heterogeneity for each meta-analysis (k= number of independent samples, I^2= between study heterogeneity)

Table 2 outlines the results of the three meta-analyses.

There was no significant difference in EQ-5D composite scores between elderly patients before and after ICU (d= -.18, n.s).

There was a marginally significant difference in EQ-5D composite scores between elderly ICU survivors and age-matched community controls, with a small effect size (d= -.22, p= .05). These results suggest that QoL may be slightly lower in elderly ICU survivors, relative to community controls.

Elderly ICU survivors (aged over 65) had significantly lower composite scores on the EQ-5D, compared to younger ICU survivors (aged under 65), with a small-to-medium effect size (d= -.33, p <.01). This suggests that on average, QoL in elderly ICU survivors is slightly worse than younger ICU survivors.

SENSITIVITY ANALYSES

We reviewed the impact of influential cases within each analysis. One study was excluded from the community meta-analysis as a substantial outlier and influential result . If the result had not been excluded, the effect size would have been stronger (d= -1.97 – ie a larger difference in QoL favouring younger controls) but non-significant (p= .27), mainly due to large heterogeneity (I^2 = 100%). It is unclear why this study reported substantially outlying results, although the reported standard deviations were considerably lower than other studies.

After excluding this, one other study was somewhat influential within the community analysis (see Appendix) . This study was retained as we acquired the full dataset and we can therefore be confident of its reporting accuracy. If this study was excluded, the effect size would have been weaker (d= -.13) and non-significant (.01) in the same direction.

We identified no further outliers according to our criteria.

SECONDARY ANALYSES

There was moderate-to-large heterogeneity between studies. For significant results, we explored the role of other variables using post-hoc subgroup analyses and meta-regressions. These results should be interpreted with caution, due to low sample sizes.

Length of follow up significantly predicted greater differences in QoL between elderly ICU survivors and patients aged under 65 (k= 6, p< .001). This suggests that elderly survivors may have worse QoL in the long term and comparable QoL in the medium term.

For peer review only - http://bmjopen.bmj.com/site/about/guidelines.xhtml

Quality of Life in elderly ICU survivors before the COVID-19 pandemic:
A Systematic Review and Meta-Analysis of Cohort Studies.

The minimum age of the sample significantly predicted greater differences in QoL between elderly ICU survivors and age-matched community controls (k= 10, p= .02). Subgroup analyses revealed that in studies with only very old patients (aged 75-80+), elderly ICU survivors' QoL was no worse than their age-matched community controls (k= 6, d= -.06, p> .05). In contrast, when elderly was defined as 65-70+, elderly ICU survivors had much worse QoL than age-matched community controls (k= 4, d= .45, p< .03). This suggests that 'very-old' ICU survivors may have comparable QoL to their age-matched peers, whereas 'young-old' ICU survivors may have worse QoL in comparison.

Controlling for these variables reduced heterogeneity between studies by 10% and 47%, in both cases. No model significantly accounted for variance when the outlier was included in the community analysis.

Neither severity of illness, year of publication or sex significantly accounted for heterogeneity between the studies, either individually or within a meta-regression (p> .05).

RISK OF BIAS

We found no evidence of publication bias for the community or pre-ICU meta-analyses, from either funnel plots or Egger's test (all p> .05). There was some evidence of funnel plot asymmetry for the young vs. old comparison (p= .04). Most studies had a moderate degree of methodological quality (14/18). We had insufficient power to explore the effect of study quality on quantitative outcomes.

QUALITATIVE SYNTHESIS

To compare different aspects of QoL, we categorised the subscales into either mental or physical health QoL and calculated a scaled average to enable comparisons (see Table 3). 16/22 studies reported the subscales for both conditions. Our estimates suggest that elderly ICU survivors reported higher average scores on mental health items (M= 57.90/100) than physical health items (M= 50.99/100). Trends in physical health scores compared less favourably to age-matched community controls than did mental health scores (mean differences = -5.23 and -1.71, respectively). Trends in physical health scores were also lower in comparison to younger ICU controls (mean difference = -2.63) whereas mental health scores were higher (mean difference = 2.65).

For peer review only - http://bmjopen.bmj.com/site/about/guidelines.xhtml

Quality of Life in elderly ICU survivors before the COVID-19 pandemic:
A Systematic Review and Meta-Analysis of Cohort Studies.

First Author	Comparison	Measure	Mean MH (Elder ICU survivor)	Mean MH (Comparison)	Mean Difference	Mean PH Score (Elder ICU survivor)	Mean PH (Comparison)	Mean Difference
Anderson	Community	EQ5D	58.62	55.87	2.75	47.27	48.46	-1.19
De Rooij	Community	EQ5D	56.86	58.22	-1.35	48.89	50.49	-1.60
Merlani	Community	SF36	43.00	47.00	-4.00	36.00	42.00	-6.00
Jeitziner	Community	SF36	69.72	80.37	-10.65	62.71	77.91	-15.20
Villa	Community	SF36	62.40	61.50	0.90	66.60	67.90	-1.30
Garrouste-Orgeas	Community	NHP	67.13	83.00	-15.87	53.63	70.23	-16.60
Schroder	Community	SF36	56.93	54.30	2.64	38.36	43.71	-5.35
Tabah	Community	WHOQOL	73.30	61.40	11.90	62.10	56.70	5.40
Average	*Community*		*61.00*	*62.71*	*-1.71*	*51.94*	*57.18*	*-5.23*
Grace	PreICU	EQ5D	61.67	54.00	7.67	58.50	53.22	5.28
Cuthbertson	PreICU	SF36	51.40	50.80	0.60	37.30	31.40	5.90
Hofhuis	PreICU	SF36	51.20	50.10	1.10	38.60	38.80	-0.20
Jeitziner	PreICU	SF36	69.72	69.02	0.70	62.71	63.63	-0.92
Average	*PreICU*		*58.50*	*55.98*	*2.52*	*49.28*	*46.76*	*2.51*
Abelha	Young	SF36	48.50	47.50	1.00	46.50	48.50	-2.00
Cuthbertson	Young	SF36	51.40	51.30	0.10	37.30	37.50	-0.20
Hofhuis	Young	SF36	51.20	50.40	0.80	38.60	38.70	-0.10
Honselmann	Young	EQ-5D	51.67	51.00	0.67	44.00	54.00	-10.00
Schroder	Young	SF36	56.93	54.30	2.64	38.36	43.71	-5.35
Eddleston	Young	SF36	63.59	58.58	5.01	58.76	63.25	-4.49
Kleinpell	Young	QLI	76.26	67.93	8.32	66.33	62.60	3.73
Average	*Young*		*57.08*	*54.43*	*2.65*	*47.12*	*49.75*	*-2.63*

Table 3. An overview of Quality of Life subscores, by mental health and physical health categories, for elderly ICU survivors and comparison groups. All scores were recalculated on a 0-100 (0 = minimum QoL; 100 = maximum QoL).
Abbreviations: MH= Mental Health; PH=Physical Health

For peer review only

Quality of Life in elderly ICU survivors before the COVID-19 pandemic:
A Systematic Review and Meta-Analysis of Cohort Studies.

DISCUSSION

This review has systematically evaluated the literature on QoL for elderly ICU survivors in the medium to long term, using EQ-5D composite scores. To our knowledge this is the first meta-analysis to address this issue. We found no evidence of worse QoL after ICU, compared to a period before ICU. However, elderly patients who survive ICU can be expected to have slightly worse QoL, compared to younger survivors. To a lesser extent, they may also have worse QoL compared to age-matched community controls. The wide prediction intervals also suggest that age differences can vary considerably in either direction.

STRENGTHS IN RELATION TO THE LITERATURE

For the meta-analysis, we identified 2442 elderly ICU survivors within an international sample of 19 cohort studies. We only included recent studies that used validated QoL measures and we rated most studies as having moderate or higher methodological quality. By pooling these samples using rigorous methods, we have been able to overcome several methodological limitations associated with generalising from individual studies, including small samples, choice of analysis and site selection bias. Our sensitivity analyses showed that the remaining heterogeneity was partly due to conceptually relevant variables. Given the relatively small literature, these methods ensure that valid, transparent results inform policy and clinical practice decisions.

Although contested, previous reviews have generally concluded that age alone is not a suitable determinant of potential benefit from ICU, especially for survivors [3 5 19 20] . The present study supports these conclusions, although the differences compared to younger ICU survivors (and to a lesser extent, community samples) are still noteworthy . Decisions on whether to admit patients can be extremely difficult for all involved, with seriously ill elderly people overrepresented among the most contentious cases [21]. These challenges are amplified further when healthcare resources are under pressure, such as during the COVID-19 pandemic.

The age-QoL associations we have found may be explained by intermediary variables. Some research suggests that frailty may best explain age differences in QoL following ICU [5 22], and clinical outcome in COVID-19 patients [23]. Frailty is a more integrative approach to conceptualising ageing, but it was not reported within the eligible studies. We would also recommend a meta-analysis of individual patient data for COVID-19 patients, to further stratify clinical variables of interest, including frailty, and to better predict QoL outcomes.

Health economic analysis of ICU in the elderly based on QALYs may be informative when it comes to resource allocation policies, but we have found few such analyses and no explicit polices based on them. They will have to grapple with the controversial notion that everyone is entitled to a 'normal' span of health or 'a fair innings' [24 25]. Given the presumption that a sizeable proportion of elderly survivors will enjoy a good QoL it is crucial that holistic, person-centred decision making is not crowded out by survival statistics or anticipatory triage. If triage were to become necessary on the front line, we would advise against weighing age too heavily against considerations pertaining QoL and rather taking more account of frailty after appropriate consultations.

On average, QoL scores gradually decline with age at approximately 0.5 points per year on the CASP-19 (range 0-57) with a modestly accelerated decrease with older age (>85 years) [4].

For peer review only - http://bmjopen.bmj.com/site/about/guidelines.xhtml

Quality of Life in elderly ICU survivors before the COVID-19 pandemic:
A Systematic Review and Meta-Analysis of Cohort Studies.

It is relevant to consider whether change in QoL in the elderly is primarily due to physical health and mental health components. We were unable to incorporate physical and mental subscores into the meta-analysis due to differences in the levels of data between measures, so we performed a qualitative synthesis. This suggested that for elderly ICU survivors, mental health questionnaire items were relatively unaffected. The small literature on older adults also suggests relatively low rates of anxiety [26] and depressive disorders [27 28], although potentially high rates of post-traumatic stress. [29] Further mental health data QoL is needed, as some preliminary reports suggest high rates of posttraumatic stress in COVID-19 ICU patients [30 31]. The determinants of posttraumatic stress are complex and our results may serve as a baseline for comparison of QoL outcome data following the COVID-19 pandemic.

LIMITATIONS

The primary limitation is the small number of eligible studies for each analysis. To maximise the sample, we included some studies with a small amount of missing data and used validated methods to estimate the mean or the standard deviation from the reported statistics. We argue that these approaches are justified as, based on central limit theorem, we expect the larger sample sizes to produce a better estimate of population variance [32]. For balance, we have also provided a comprehensive overview of our sensitivity analyses to assess risk of bias (see Appendix). These demonstrate that although our decisions reduced bias, most did not change our interpretation of the effects.

Another potential limitation of the meta-analysis is the focus on long-term ICU survivors, as reported mortality rates were as high as 80% at follow up. We argue that a substantial 'healthy survivor' effect on QoL is unlikely because survival and QoL have different pathophysiological determinants. We also did not find any evidence of better QoL for elderly patients in studies with high mortality rates. Nevertheless, our results clearly extend only to ICU survivors, rather than prospective ICU patients.

Our results may also be prone to other selection biases. Compared to younger adults, unhealthy elderly adults might be less likely to be admitted to into ICU [21], to survive ICU treatment (possibly in part due to decisions around lifesaving treatment [33]) and to survive until follow-up. It was also unclear how many patients had pre-existing cognitive impairments where QoL measurement is more complex, although there was no indication that the proportion was large. Without further data on contextual variables, we would caution generalising our findings on QoL to all elderly ICU patients. Nonetheless, these results imply that at least some elderly ICU patients will have a relatively good QoL in the medium-to-long term.

Data describing QoL at follow-up of elderly survivors admitted to ICU with a diagnosis of COVID-19 were not available at the time of data extraction, we were therefore unable to include in the sample this sub-group of patients. Future studies will need to consider elderly COVID-19 survivors, who often require a relatively lengthy period of ICU treatment and post-ICU rehabilitation.

We were unable to assess quality of life as rigorously as we would have liked. This was partly because studies varied in their definitions of 'old age'. Most of the eligible studies defined this as 65+, following the World Health Organisation definition [34]. However, patients aged 65+ account for roughly half of all ICU admissions [35]. It is therefore likely that a higher

For peer review only - http://bmjopen.bmj.com/site/about/guidelines.xhtml

Quality of Life in elderly ICU survivors before the COVID-19 pandemic:
A Systematic Review and Meta-Analysis of Cohort Studies.

threshold would be more relevant to investigate age-related syndromes. A consensus on what should count as 'very old' would help data collection, analysis and interpretation within this field.

The scores for pre-ICU scores were determined by retrospective ratings from discharged patients or proxies. This is usual practice, but the reliability of proxies is contested [36 37]. Ideally, we would have analysed differences in QoL change scores between younger and elderly ICU survivors, at multiple time points from before ICU to follow up.

Finally, we observed moderate-to-high levels of heterogeneity between studies, which limits the generalisability of the results. We found that much of this variation may have been due to mortality rates and length of time post-discharge, which supports the view that age alone is not a strong predictor of QoL outcome. We also tried to ensure consistency of measurement by using a mapping function between SF-36 scores to EQ-5D scores, which is a common approach within NICE guidelines[13 38].

For peer review only - http://bmjopen.bmj.com/site/about/guidelines.xhtml

Quality of Life in elderly ICU survivors before the COVID-19 pandemic:
A Systematic Review and Meta-Analysis of Cohort Studies.

CONCLUSION

Our study reports the first known meta-analysis of quality of life in elderly patients following ICU. We report that on average, elderly ICU survivors have similar QoL compared to before their admission. They have slightly worse QoL compared to younger ICU survivors and possibly compared to age-matched community controls. These differences were based on physical rather than mental health. Our findings add rigour to the current literature and should inform debates around population level resource allocation and person-centred intensive care decision making during the current COVID-19 pandemic and after.

CORRESPONDENCE

Mr. Kevin Ariyo MSc (PhD candidate), Mental Health and Justice Project, Department of Psychological Medicine, Institute of Psychiatry, Psychology and Neuroscience, King's College London. 16 De Crespigny Park, Camberwell, London, SE5 8AF. Email: kevin.ariyo@kcl.ac.uk.

AUTHOR STATEMENT

K.A. led at each stage of the project, including drafting the document. G.O. was primary supervisor on the project, jointly formulated the research questions, led on writing the introduction section and made substantial contributions to all aspects of the study. S.C. advised on the initial protocol and provided critical revisions from an intensivist perspective. A.D. and A.R.K. provided additional supervision and critical revisions. S.W. also contributed to data collection and analysis, by providing previously unpublished data, and critical revisions.

The manuscript is a transparent account of the study being reported and adheres to PRISMA reporting guidelines. All listed authors have approved for the manuscript to be published in its current format and meet all the ICMJE criteria for authorship. The authors agree to be accountable for the contents of the paper and are jointly responsible for ensuring that all queries related to the accuracy or integrity of the project are investigated and resolved.

ACKNOWLEDGEMENTS

We are grateful to Margot Kuylen for her contributions to the reliability assessment and to John Brazier for advising on the SF-36 to EQ-5D mapping function.

ETHICAL APPROVAL

Not required.

DATA SHARING

The datasets generated and analysed during the current study are included in this published article and its supplementary information files. Any data queries may also be directed to the corresponding author on reasonable request.

FUNDING

Supported by the Mental Health and Justice Project, led by G.O., which is funded by a grant from the Wellcome Trust (203376/2/16/Z).

For peer review only

Quality of Life in elderly ICU survivors before the COVID-19 pandemic:
A Systematic Review and Meta-Analysis of Cohort Studies.

REFERENCES

1. Archard D, Caplan A. Is it wrong to prioritise younger patients with covid-19? *BMJ* 2020;369
2. Savulescu J, Cameron J, Wilkinson D. Equality or utility? Ethics and law of rationing ventilators. *British Journal of Anaesthesia* 2020
3. Hennessy D, Juzwishin K, Yergens D, et al. Critical care review. Outcomes of elderly survivors of intensive care: a review of the literature. *CHEST* 2005;127(5):1764-74.
4. Zaninotto P, Falaschetti E, Sacker A. Age trajectories of quality of life among older adults: results from the English Longitudinal Study of Ageing. *Quality of Life Research* 2009;18(10):1301-09.
5. Conti M, Merlani P, Ricou B. Prognosis and quality of life of elderly patients after intensive care. *Swiss Med Wkly* 2012;142:w13671. doi: 10.4414/smw.2012.13671 [published Online First: 2012/09/12]
6. Zhou F, Yu T, Du R, et al. Clinical course and risk factors for mortality of adult inpatients with COVID-19 in Wuhan, China: a retrospective cohort study. *The lancet* 2020
7. Willan J, King AJ, Jeffery K, et al. Challenges for NHS hospitals during covid-19 epidemic: British Medical Journal Publishing Group, 2020.
8. de Castro-Hamoy L, de Castro LD. Age Matters but it should not be Used to Discriminate Against the Elderly in Allocating Scarce Resources in the Context of COVID-19. *Asian Bioethics Review* 2020:1-10.
9. Haas LE, de Lange DW, van Dijk D, et al. Should we deny ICU admission to the elderly? Ethical considerations in times of COVID-19. *Critical Care* 2020;24(1):1-3.
10. Montero-Odasso M, Hogan DB, Lam R, et al. Age alone is not adequate to determine healthcare resource allocation during the COVID-19 pandemic. *Canadian Geriatrics Journal* 2020;23(1):152-54.
11. Rubenfeld GD. Does the hospital make you older faster?: American Thoracic Society, 2012.
12. Landis JR, Koch GG. The measurement of observer agreement for categorical data. *biometrics* 1977:159-74.
13. Ara R, Brazier J. Deriving an algorithm to convert the eight mean SF-36 dimension scores into a mean EQ-5D preference-based score from published studies (where patient level data are not available). *Value in Health* 2008;11(7):1131-43.
14. Cohen J. Statistical power analysis for the behavioral sciences New York. *NY: Academic* 1988
15. Luo D, Wan X, Liu J, et al. Optimally estimating the sample mean from the sample size, median, mid-range, and/or mid-quartile range. *Statistical methods in medical research* 2018;27(6):1785-805.
16. Wan X, Wang W, Liu J, et al. Estimating the sample mean and standard deviation from the sample size, median, range and/or interquartile range. *BMC medical research methodology* 2014;14(1):135.
17. Ma J, Liu W, Hunter A, et al. Performing meta-analysis with incomplete statistical information in clinical trials. *BMC medical research methodology* 2008;8(1):56.
18. Kmet LM, Cook LS, Lee RC. Standard quality assessment criteria for evaluating primary research papers from a variety of fields. 2004
19. Chelluri L, Grenvik A, Silverman M. Intensive care for critically ill elderly: mortality, costs, and quality of life: review of the literature. *Archives of Internal Medicine* 1995;155(10):1013-22.

For peer review only - http://bmjopen.bmj.com/site/about/guidelines.xhtml

Quality of Life in elderly ICU survivors before the COVID-19 pandemic:
A Systematic Review and Meta-Analysis of Cohort Studies.

20. Wehler M. [Long-term outcome of elderly patients after intensive care treatment]. *Med Klin Intensivmed Notfmed* 2011;106(1):29-33. doi: 10.1007/s00063-011-0021-x [published Online First: 2011/10/07]
21. Escher M, Cullati S, Hudelson P, et al. Admission to intensive care: A qualitative study of triage and its determinants. *Health Serv Res* 2019;54(2):474-83. doi: 10.1111/1475-6773.13076 [published Online First: 2018/10/27]
22. Heyland DK, Garland A, Bagshaw SM, et al. Recovery after critical illness in patients aged 80 years or older: a multi-center prospective observational cohort study. *Intensive care medicine* 2015;41(11):1911-20.
23. Hewitt J, Carter B, Vilches-Moraga A, et al. The effect of frailty on survival in patients with COVID-19 (COPE): a multicentre, European, observational cohort study. *The Lancet Public Health* 2020
24. Williams A. Intergenerational equity: an exploration of the 'fair innings' argument. *Health economics* 1997;6(2):117-32.
25. NICE CITIZENS COUNCIL REPORT ON AGE: National Institute for Care Excellence, 2020.
26. Jeitziner MM, Hamers JP, Bürgin R, et al. Long-term consequences of pain, anxiety and agitation for critically ill older patients after an intensive care unit stay. *Journal of clinical nursing* 2015;24(17-18):2419-28.
27. Broslawski G, Elkins M, Algus M. Functional abilities of elderly survivors of intensive care. *The Journal of the American Osteopathic Association* 1995;95(12):712.
28. Chelluri L, Pinsky MR, Donahoe MP, et al. Long-term Outcome of Critically III Elderly Patients Requiring Intensive Care. *Jama* 1993;269(24):3119-23.
29. Drews T, Franck M, Radtke FM, et al. Postoperative delirium is an independent risk factor for posttraumatic stress disorder in the elderly patient: a prospective observational study. *European Journal of Anaesthesiology (EJA)* 2015;32(3):147-51.
30. Antonis T. Post-Traumatic Stress Disorder (Ptsd) In Patients with Covid 19. *Ann Clin Med Case Rep* 2021:V5.
31. de Graaf M, Antoni M, Ter Kuile M, et al. Short-term outpatient follow-up of COVID-19 patients: A multidisciplinary approach. *EClinicalMedicine* 2021;32:100731.
32. Weir CJ, Butcher I, Assi V, et al. Dealing with missing standard deviation and mean values in meta-analysis of continuous outcomes: a systematic review. *BMC medical research methodology* 2018;18(1):25.
33. Ferrand E, Robert R, Ingrand P, et al. Withholding and withdrawal of life support in intensive-care units in France: a prospective survey. *The Lancet* 2001;357(9249):9-14.
34. BRUNDTLAND G. Achieving health across the life span. *Men Aging and Health* 1999
35. Boumendil A, Somme D, Garrouste-Orgeas M, et al. Should elderly patients be admitted to the intensive care unit? *Intensive Care Med* 2007;33(7):1252. doi: 10.1007/s00134-007-0621-3 [published Online First: 2007/04/04]
36. Capuzzo M, Grasselli C, Carrer S, et al. Quality of life before intensive care admission: agreement between patient and relative assessment. *Intensive care medicine* 2000;26(9):1288-95.
37. Dinglas VD, Gifford JM, Husain N, et al. Quality of life before intensive care using EQ-5D: patient versus proxy responses. *Critical care medicine* 2013;41(1):9.
38. Longworth L, Rowen D. Mapping to obtain EQ-5D utility values for use in NICE health technology assessments. *Value in health* 2013;16(1):202-10.

For peer review only - http://bmjopen.bmj.com/site/about/guidelines.xhtml

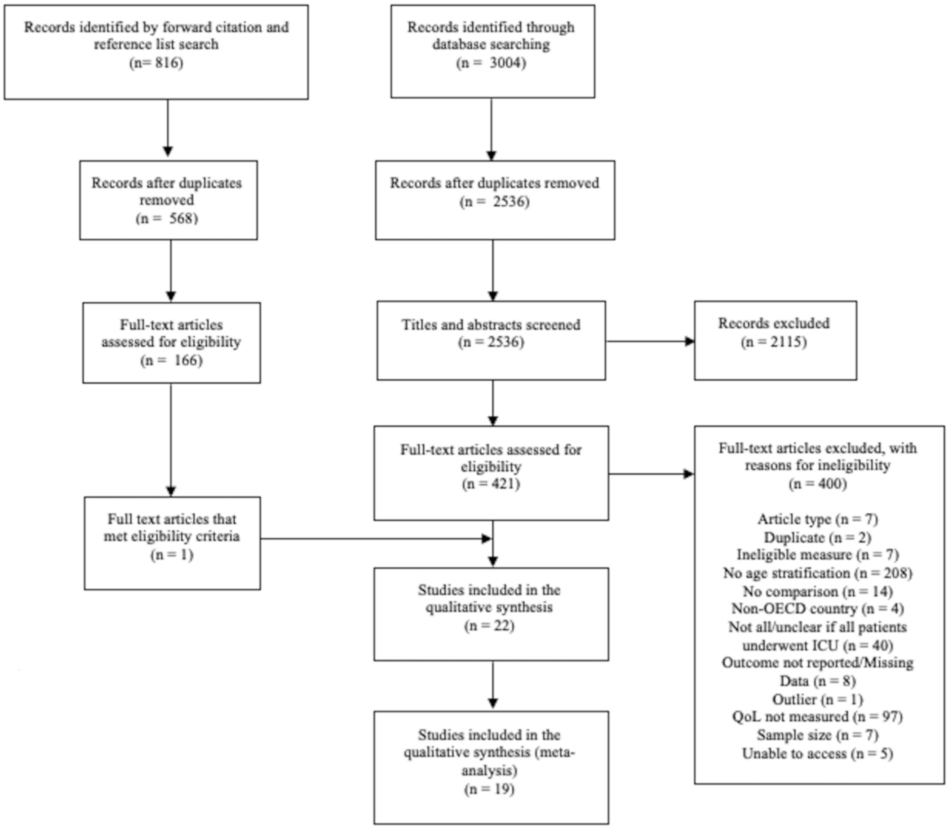

Figure 1. A PRISMA flow diagram that outlines the study selection process.

1. STUDY CHARACTERISTICS

1.1 Meta-Analysis

First Author	Year	Country	Study Design	Journal	Setting	Min Age	Avg. Age (SD)	% Male	Mortality	ICU LoS (SD)	HLoS (SD)	Severity	Raw Measure	Follow up	Comparison	Study Quality	Participant No.	Control No.	Effect Size	Variance
Abelha[39]	2007	Portugal	Cohort (unspecified)	BMC Anaesthesiology	Surgical ICU	65+		61.00%	28.00%				SF-36 *	6 months	ICU survivors younger than 65 years old	M	114	112	-.07	.02
Ali [38]	2018	Australia	Prospective Cohort	Journal of Critical Care	Medical-Surgical ICU	65+	73 (5)	80.00% [a]		4.64 (2.32)	16.29 (9.28)	.24	EQ-5D	12 months	Age-matched South Australian controls	H	32	572	.03	.03
Andersen [37]	2015	Norway	Retrospective Cohort	Annals of Intensive Care	General Hospital ICU	80+	87.4 (4)	69.00%	81.52%	1.9 (NR)		.27	EQ-5D	40.8 months	Age and sex-matched Norwegian population	M	53	170	-.18	.02
Cuthbertson [36]	2005	UK	Prospective Cohort	Critical Care	General Hospital ICU	65+		59.00%[a]	33.00%				SF-36 *	12 months	ICU survivors younger than 65 years old	M	62	116	.17	.02
De Rooij [35]	2008	Netherlands	Retrospective Cohort	Journal of the American Geriatric Society	Medical-Surgical ICU	80+	81.7 (2.4)	51.00%	61.52%	1.29 (1.13)		.21	EQ-5D	44.4 months	Age-matched British population	M	187	142	-.24	.01
Eddleston [34]	2000	UK	Prospective Cohort	Critical Care Medicine	General Hospital ICU	65+		52.45%[a]					SF-36 *	3 months	ICU survivors younger than 65 years old	M	39	97	-.21	.04
Ferrao [33]	2015	Portugal	Retrospective Cohort	Critical Care	Medical-Surgical ICU	66+ [b]		26.00%					EQ-5D	27.6 months	ICU survivors younger than 65 years old	M	290	652	-.37	.01
Grace [31]	2007	Australia/NZ	Retrospective Cohort	Critical Care and Resuscitation	Mixed ICUs	60+		NR	60.00%			.28	EQ-5D	28 months	Retrospective patient ratings for one week before ICU	L	99	99	-.36	.02
Hofhuis [30]	2011	Netherlands	Prospective Cohort	Chest	Medical-Surgical ICU	80+ [b]	83 (3.06)	46.90%	40.83%	5.35 (2.29)	25.48 (16.04)	.25	SF-36 *	6 months	Age-matched Dutch population	M	49	49 [c]	.26	.04
															Retrospective proxy ratings for four weeks before ICU		49	49	.01	.04
Honselmann	2015	Germany	Retrospective Cohort	Journal of Critical Care (part unpublished)	Mixed ICU (unpublished)	65+	75.84	53.00%	43.00%	2.58 (NR)			EQ-5D	12 months	ICU survivors	N/A (unpublished)	352	249	.90	.00

															younger than 65 years old					
							75.16	54.00%	43.00%	2.34			EQ-5D	12 months	Age-matched German controls	N/A (unpublished)	291	828	.41	.00
Jeitziner [29]	2015	Switzerland	Retrospective Cohort	Journal of Clinical Nursing	Medical-Surgical ICU	65+	68.72 (5.39)	73.00%		4.57 (5.81)		.29	SF-36 [*]	12 months	Age matched Swiss controls;	M	124	145	-.59	.02
															Retrospective patient ratings for one week before ICU		124	135	-.08	.01
Kaarola [28]	2006	Finland	Cross-Sectional	Critical Care Medicine	Medical-Surgical ICU	65+		75.00%	57.00%				EQ-5D	47 months	ICU survivors younger than 65 years old	M	299	800	-.67	.00
Levinson [26]	2016	Australia	Prospective Cohort	Internal Medicine Journal	Private ICU	80+	84.59 (NR)	58.00%[a]	21.45%	1.28 (NR)	12.91 (NR)		SF-36 [*]	24 months	Age and sex-matched Australian population	H	322	907	.04	.00
Merlani [25]	2007	Switzerland	Retrospective Cohort	Acta Anaesthesiologica Scandinavica	Surgical ICU	70+	78 (5)	52.00%	63.00%	3.00 (13.72)	22.50 (93.88)	.26	SF-36 [*]	24 months	Age-matched Swiss population	M	36	87	-.23	.04
Oeyen [24]	2007	Netherlands	Prospective Cohort	Minerva Medica	Medical-Surgical ICU	80+	83 (3)	60.00%[a]	49.60%	3.35 (2.26)	26.93 (27.11)	.26	EQ-5D	12 months	Retrospective patient or proxy ratings for one week before ICU	M	63	63	-.30	.03
Sacanella [23]	2011	Spain	Prospective Cohort	Critical Care	Medical ICU	65+	73.4 (5.5)	57.00%	48.70%	9.4 (10.20)		.27	EQ-5D	12 months	Retrospective patient or proxy ratings before feeling ill and requiring ICU	M	112	112	-.49	.02
Schroder [22]	2011	Denmark	Cohort (unspecified)	Danish Medical Bulletin	Mixed ICUs	75+		56.00%	53.85%				SF-36 [*]	12 months	Age-matched Danish population	L	36	229	-.03	.03
Sznajer [21]	2001	France	Prospective Cohort	Intensive Care Medicine	Mixed ICUs	65+[b]		55.90%[a]					EQ-5D	6 months	ICU survivors younger than 65 years old	M	65	53	-.16	.03
Villa [19]	2016	Spain	Prospective Cohort	Journal of the American Geriatric Society	Medical-Surgical ICU	75+	80.8 (3.3)	50.00%	43.18%			.23	SF-36 [*]	12 months	Spanish population aged 75+	M	54	1363 [d]	-.15	.02

Table A1 Full study characteristics for all effect sizes included in the meta-analysis

[a] Reported for study level only
[b] Combined elderly groups
[c] Assumed N based on matched sample

[d] Retrieved from López-García, E., Banegas, J. R., Graciani, A. P. R., Gutiérrez-Fisac, J. L., Alonso, J., & Rodríguez-Artalejo, F. (2003). Population-based reference values for the Spanish version of the SF-36 Health Survey in the elderly. Medicina clinica, 120(15), 568-573; a follow-up to the previous study, which was unavailable
[e] Unless specified, we do not report data where it is not representative of at least 66.67% of the included sample
[f] Abbreviations: Avg. Age (average age); ICU LoS (average length of stay in intensive care; days); HLoS (average length of stay in hospital; days); SD (standard deviation; sometimes estimated- see methods)
NOTE: If studies are reported in duplicate, for the second row, assume blank cells are the same value as the row above, unless otherwise specified.

1.2 Qualitative Only Studies

First Author	Year	Country	Study Design	Journal	Setting	Min Age	Participant No.	Avg. Age (SD)	% Male	ICU LoS (SD)	HLoS (SD)	Severity	Ineligible Measure	Follow up	Comparison
Garrouste-Orgeas	2006	France	Prospective Cohort	Intensive Care Medicine	Medical ICU	80+	28	84 (3.92)		12.6 (15.5)		.28	Nottingham Health Profile (NHP)	12 months	Age and sex-matched French population controls
Kleinpell	2002	USA	Retrospective Cohort	Research in Nursing and Health	Mixed ICUs	66+	128		42.00%	4.2 (6.17)	10.28 (9.63)	.18	Quality of Life Index (QLI)	4-6 months	ICU survivors aged between 45 and 64 years old
Tabah	2010	France	Prospective Cohort	Critical Care	Medical-Surgical ICU	80+	23	84 (3)	73.90%	5.72 (4.74)	18.08 (15.01)	.23	WHO-QOL-BREF	16 months	Age and sex-matched French population controls

Table A2 Full study characteristics of all records that were only included in the qualitative synthesis
[a] Reported for study level only
[b] Abbreviations: Avg. Age (average age); ICU LoS (average length of stay in intensive care; days); HLoS (average length of stay in hospital; days), SD (standard deviation; sometimes estimated- see methods)
[c] Unless specified, we do not report data where it is not representative of at least 66.67% of the included sample.

For peer review only

2. SENSITIVITY ANALYSES FOR INFLUENTIAL CASES

2.1 Overview of Outliers: Meta-Analysis

Comparison	k	First Author	Cook's Distance (Critical d)	Leave out Effect Size	Leave out P value	I^2 Change	Effect Size Change
Community	11	Pavoni	.97 (.36)	-1.97	.27	-12%	+1.74
Community	10	Honselmann	.56 (.40)	-.13	.10	-21%	+.08

Table A3 A summary of cases that fit our criteria as potentially influential
[a] Excluded cases are highlighted in red

First Author	Year	Country	Study Design	Journal	Setting	Min Age	Participant No.	Avg. Age (SD)	% Male	ICU LoS (SD)	HLoS (SD)	Severity	Mortality	Follow up	Comparison
Pavoni	2012	Italy	Prospective Cohort	Archives of Gerontology and Geriatrics	Mixed ICUs	80+	143	86.51 [a] (1.81)	26.74% [a]	5.27 [a] (5.80)	14.20 [a] (8.96)	.20 [a]	50% [a]	12 months	Age-matched Italian retirement community population

Table A4 Study characteristics of the lone study excluded as an outlier
[a] Reported for study level only
[b] Abbreviations: Avg. Age (average age); ICU LoS (average length of stay in intensive care; days); HLoS (average length of stay in hospital; days), SD (standard deviation; sometimes estimated- see methods)

For peer review only

3. QUALITATIVE SYNTHESIS

3.1 Qualitative analysis procedure

Scale	Mental Health Subscale(s)	Physcial Health Subscale(s)	Additional Notes
EQ-5D	Anxiety/Depression	Mobility, Self-Care, Usual Activities, Pain/Discomfort	Raw scores scaled between 1-3
SF-36	Social Functioning, Role Emotional, Mental Health, Vitality	Physical Functioning, Bodily Pain, General Health, Role Physical	
NHP	Sleep, Emotional Reaction, Social Isolation	Pain, Energy, Physical Mobility	Reverse scoring
WHO-QOL-BREF	Psychological Health, Social Relationships	Overall perception of Health, Physical Health, Environment	
QLI	Socio-economic, Family, Psychological/Spiritual	Health and Functioning	Raw scores scaled between 0-30

Table A5 Subscales used to estimate mental and physical health QoL within the qualitative synthesis

For peer review only

4. SENSITIVITY ANALYSES FOR OBSERVED EFFECTS

4.1 Forest Plots

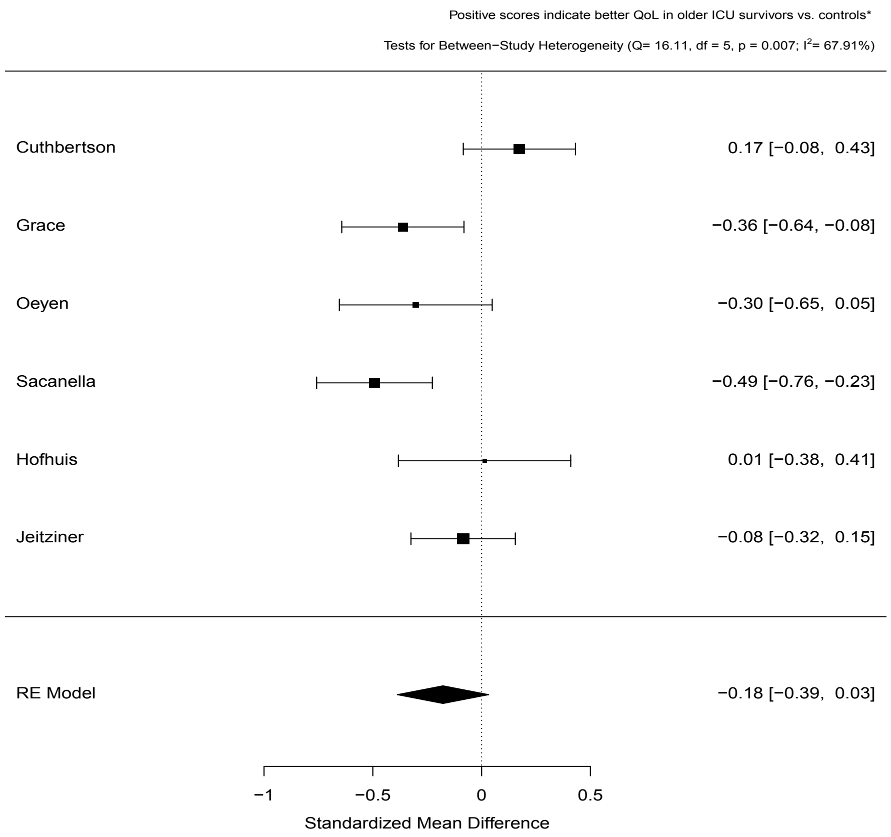

Fig. A1 Forest plot of differences in EQ-5D composite scores in elderly survivors, comparing pre-ICU and post-ICU scores

For peer review only - http://bmjopen.bmj.com/site/about/guidelines.xhtml

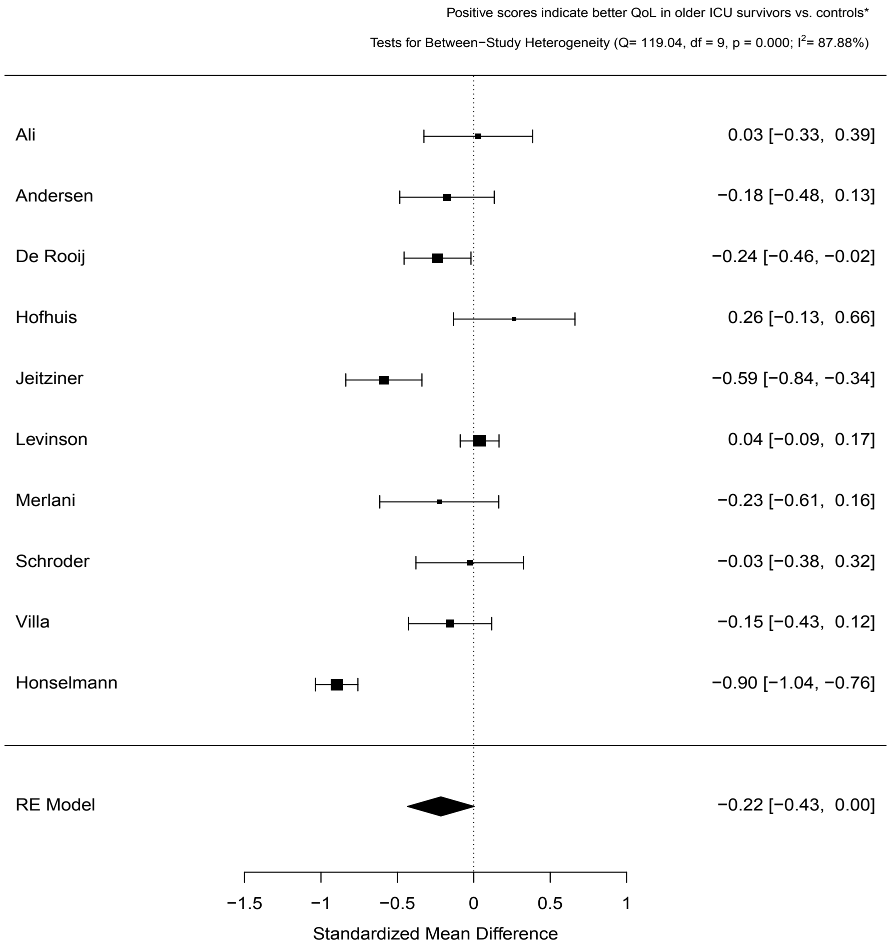

Fig. A2 Forest plot of differences in EQ-5D composite scores, comparing elderly ICU survivors at follow-up and age-matched community controls

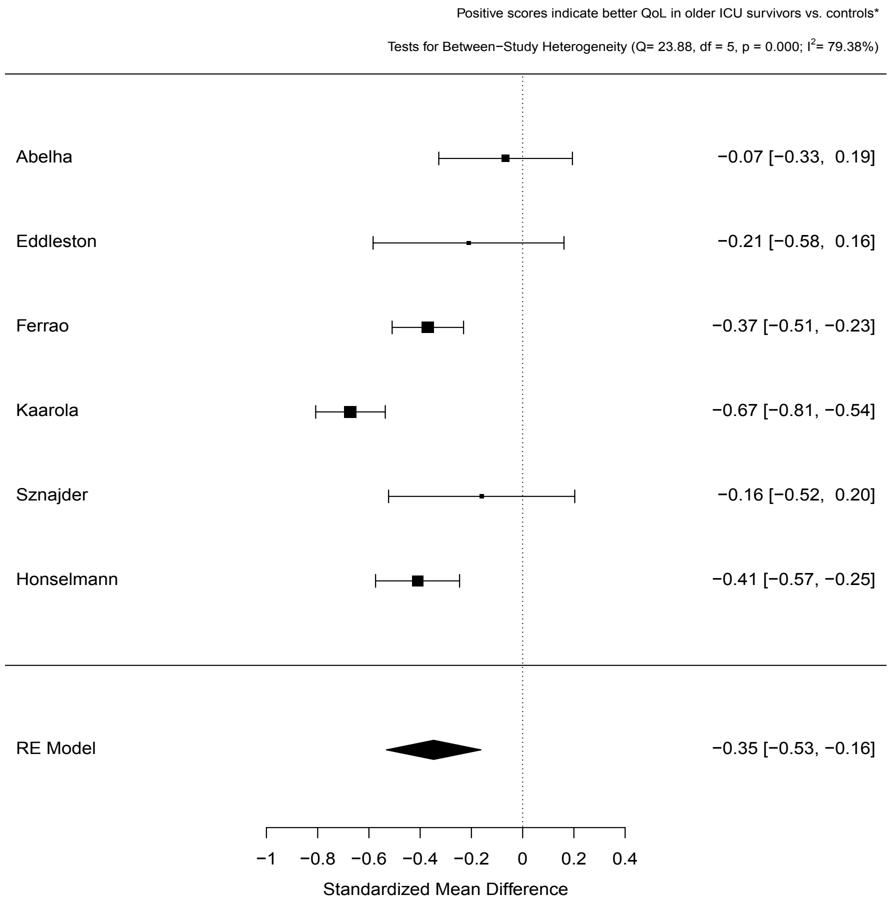

Fig. A3 Forest plot of differences in EQ-5D composite scores at follow-up, comparing elderly ICU survivors (aged 65+) and younger ICU survivors (aged under 65), both at follow-up

For peer review only - http://bmjopen.bmj.com/site/about/guidelines.xhtml

Appendix: Disparity or Discrimination?
A systematic review of socio-demographic associations of insight

4.2 Funnel Plots

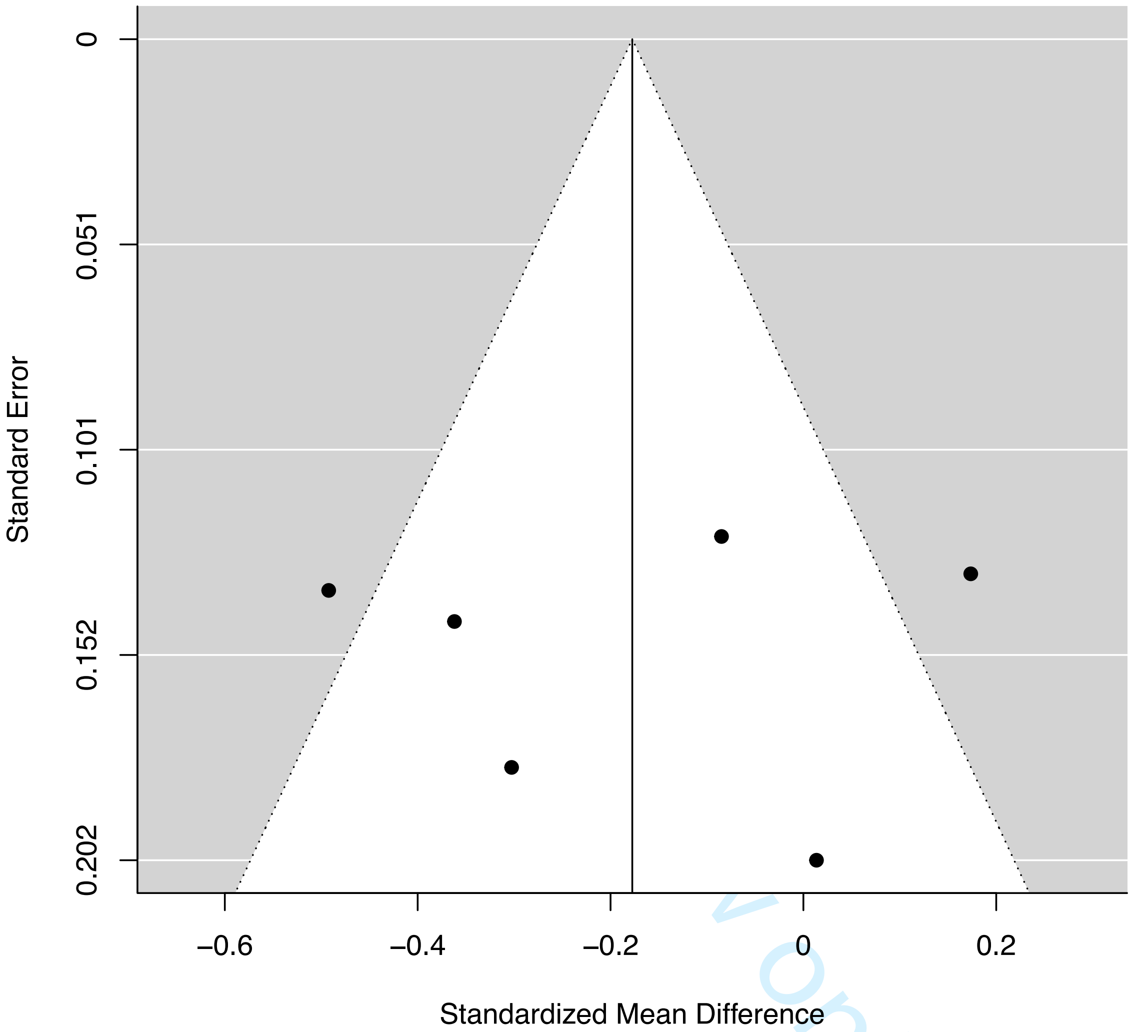

Fig. A4 Funnel plot of studies that investigated differences in EQ-5D composite scores in elderly survivors, comparing pre-ICU and post-ICU scores

For peer review only - http://bmjopen.bmj.com/site/about/guidelines.xhtml

Appendix: Disparity or Discrimination?
A systematic review of socio-demographic associations of insight

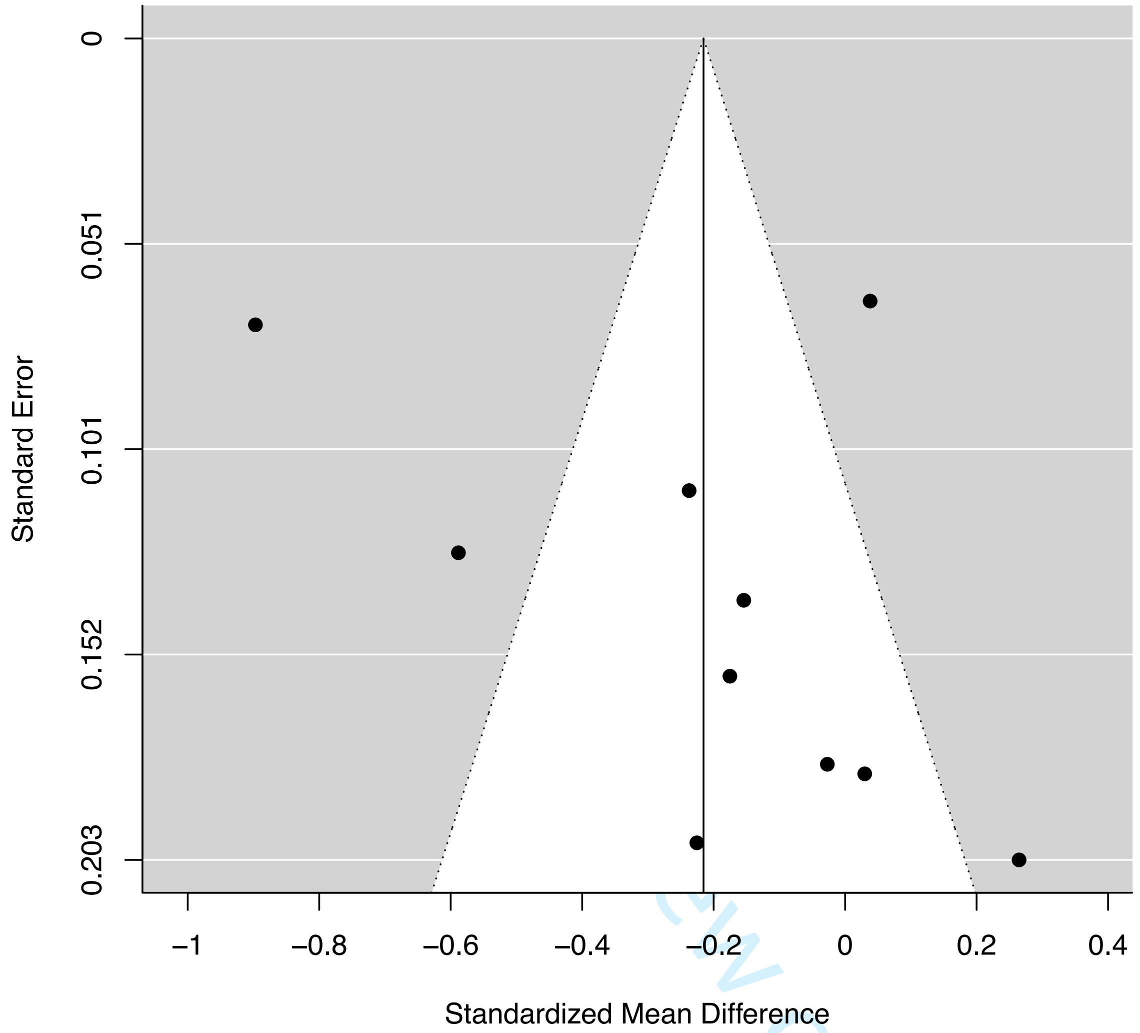

Fig. A5 Funnel plot of studies that compared EQ-5D scores in elderly ICU survivors at follow-up and age-matched community controls

For peer review only - http://bmjopen.bmj.com/site/about/guidelines.xhtml

Appendix: Disparity or Discrimination?
A systematic review of socio-demographic associations of insight

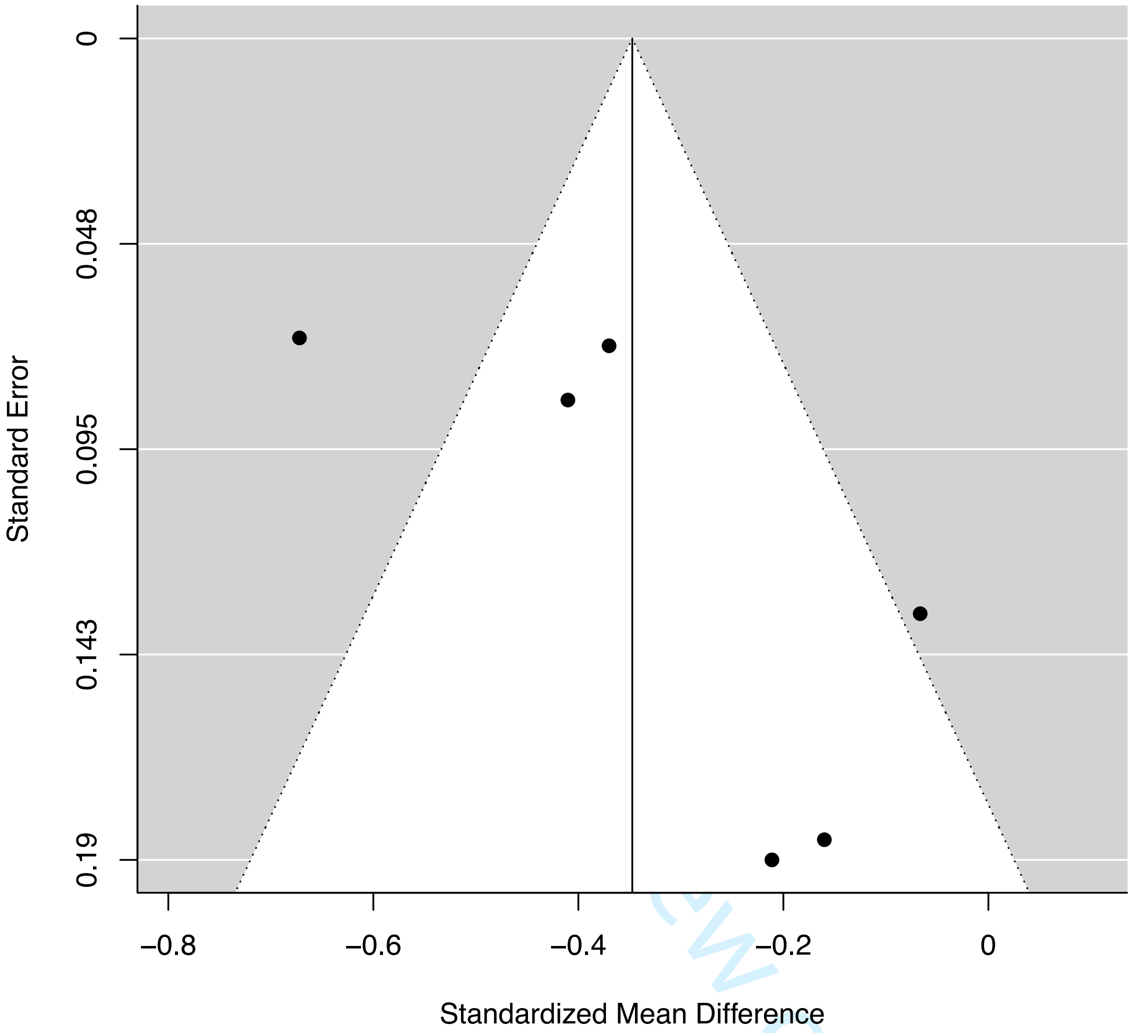

Fig. A6 Funnel plot of studies that compared EQ-5D scores in elderly ICU survivors (aged 65+) and younger ICU survivors (aged under 65), both at follow-up

For peer review only - http://bmjopen.bmj.com/site/about/guidelines.xhtml

Appendix: Disparity or Discrimination?
A systematic review of socio-demographic associations of insight

4.3 Cook's Distance Plots

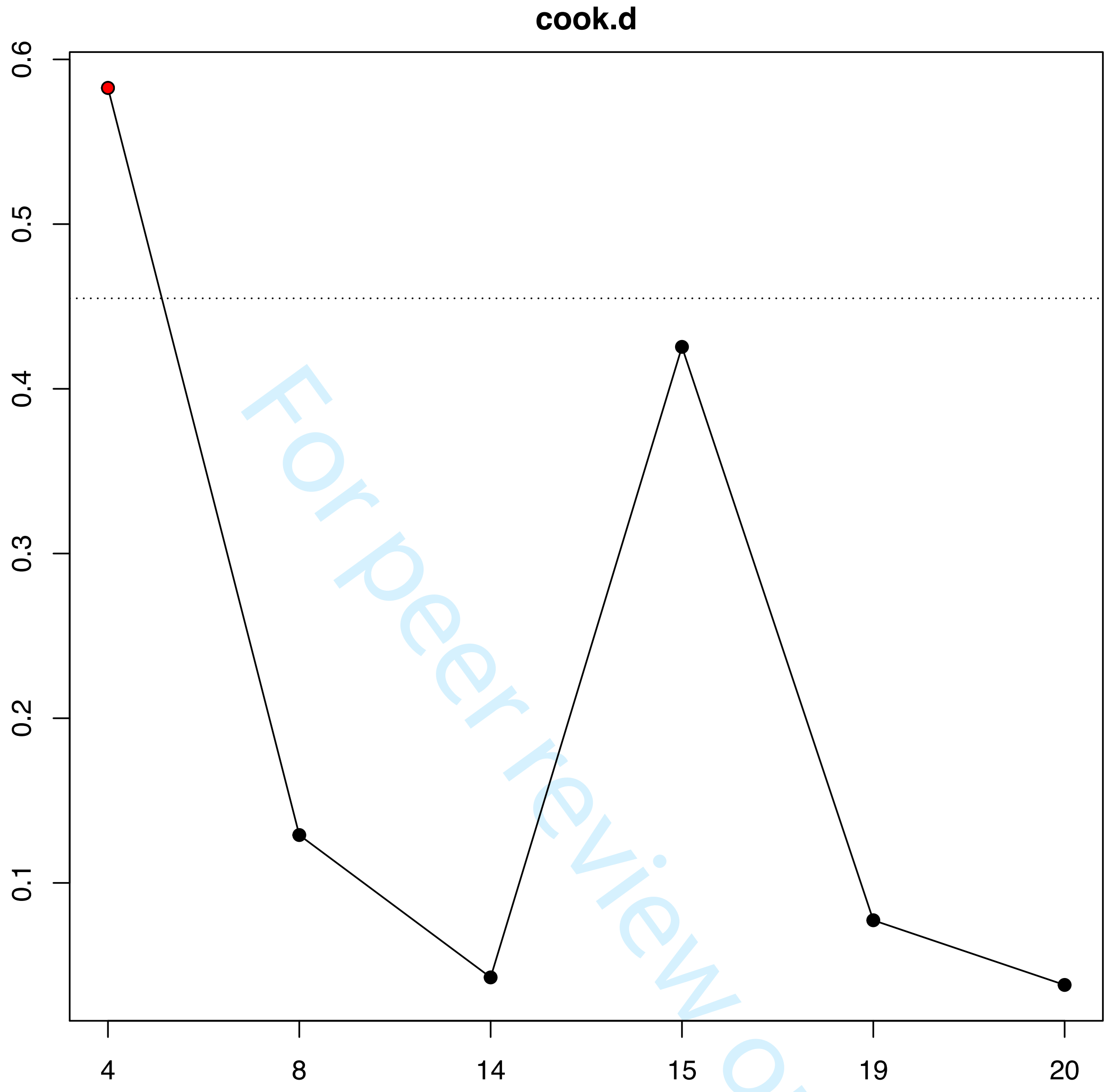

Fig. A7 Cook's distance plot of studies that investigated differences in EQ-5D composite scores in elderly survivors, comparing pre-ICU and post-ICU scores

For peer review only

For peer review only - http://bmjopen.bmj.com/site/about/guidelines.xhtml

Appendix: Disparity or Discrimination?
A systematic review of socio-demographic associations of insight

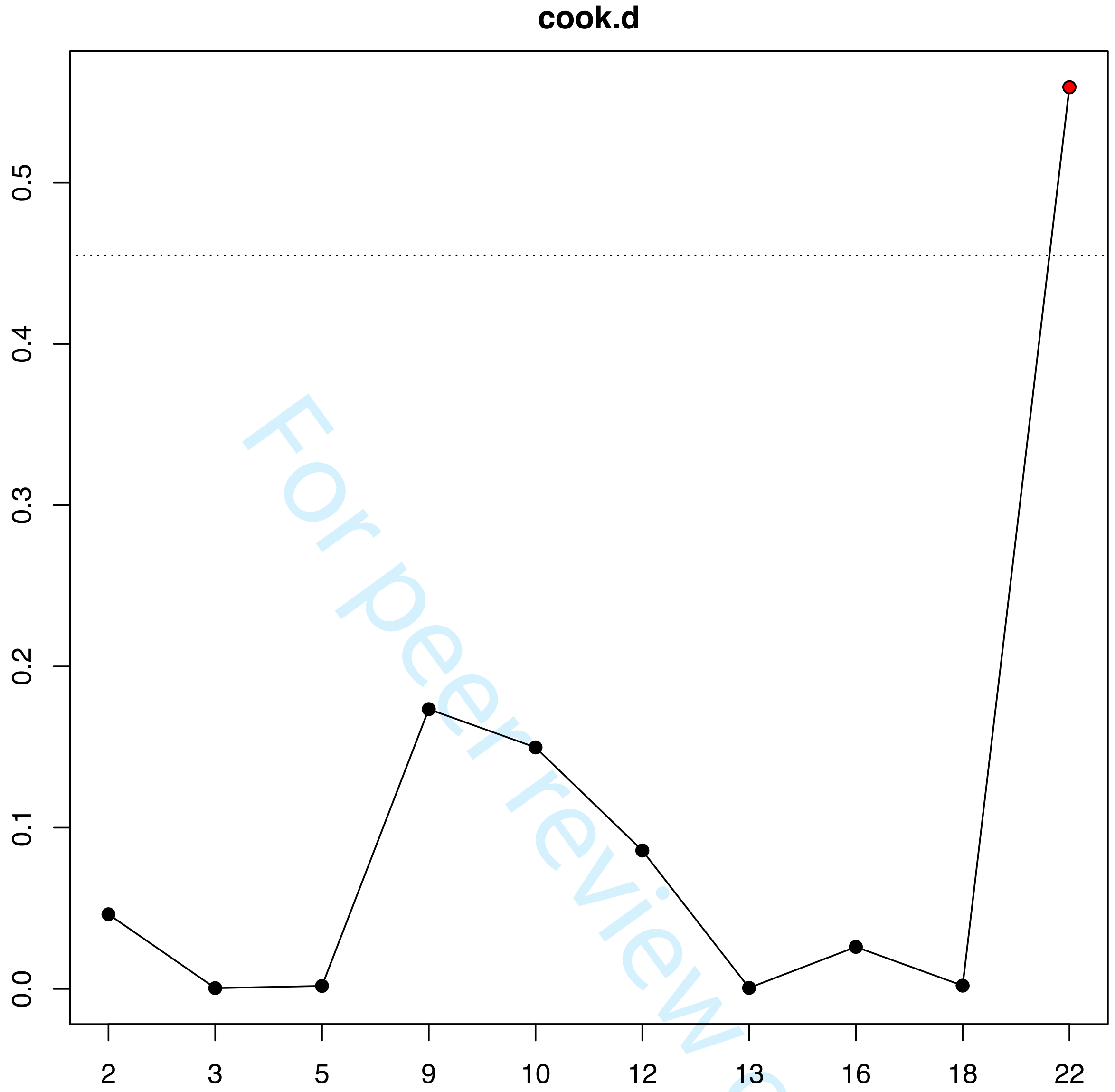

Fig. A8 Cook's distance plot of studies that compared EQ-5D scores in elderly ICU survivors at follow-up and age-matched community controls

For peer review only - http://bmjopen.bmj.com/site/about/guidelines.xhtml

Appendix: Disparity or Discrimination?
A systematic review of socio-demographic associations of insight

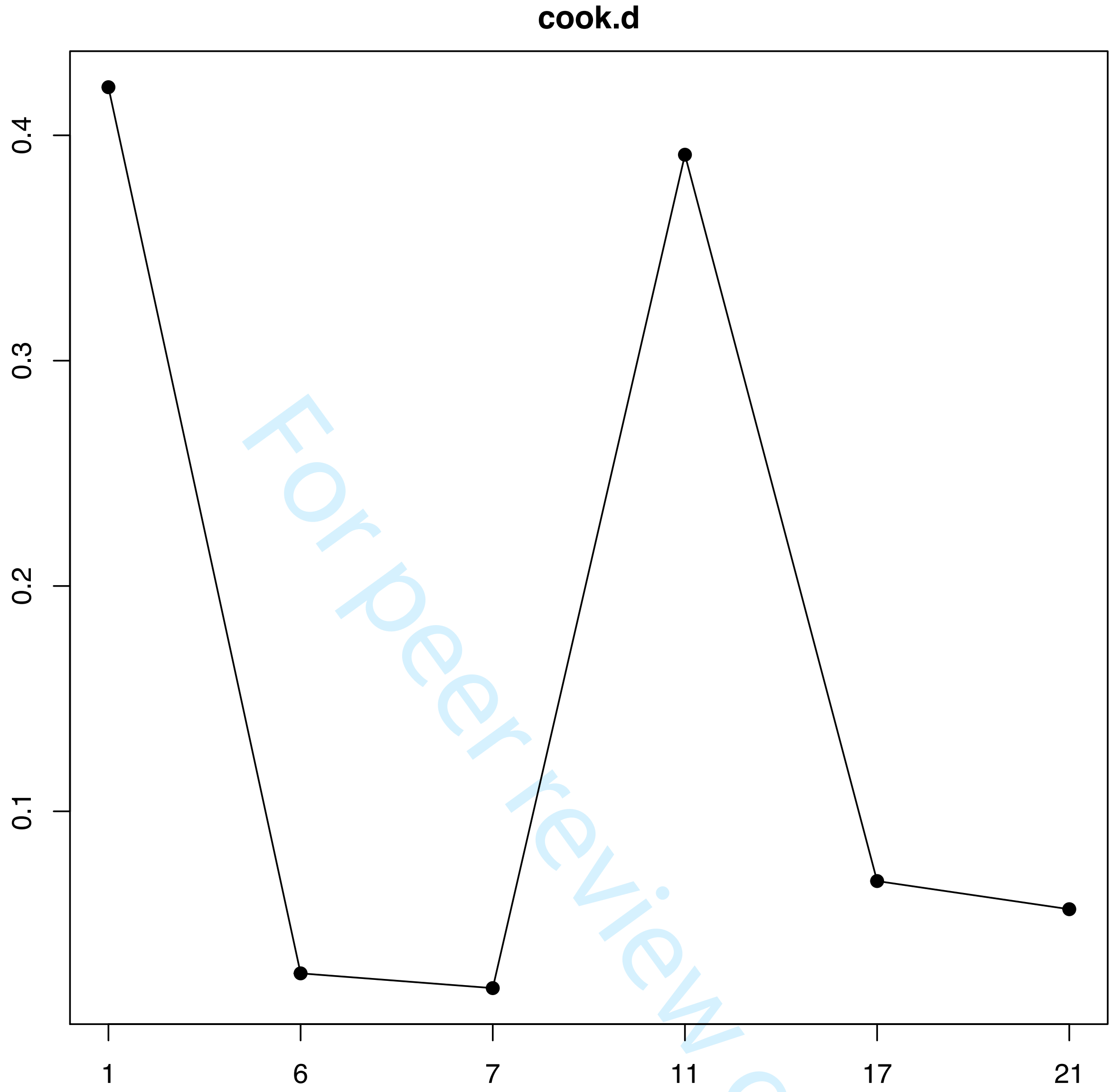

Fig. A9 Cook's distance plot of studies that compared EQ-5D scores in elderly ICU survivors (aged 65+) and younger ICU survivors (aged under 65), both at follow-up

For peer review only

For peer review only - http://bmjopen.bmj.com/site/about/guidelines.xhtml

Appendix: Disparity or Discrimination?
A systematic review of socio-demographic associations of insight

5. REVIEW PROTOCOL

5.1 ICU Review Protocol

Included	Excluded
**Design**	
Case note analyses (longitudinal)<br>Case control<br>Retrospective cohort<br>Prospective cohort<br>Unpublished dissertations of the above	Qualitative only studies<br>Systematic review or meta-analysis (categorise in separate folder)<br>Narrative review<br>Non-English language (if translation can't be found)<br>Commentaries<br>Case studies<br>Small N samples (<20 eligible participants)<br>Conference abstracts<br>Brief reports<br>Books
**Population**	
Patients aged 60+ who have undergone ICU<br>Medical, Surgical or Mixed ICU settings	<20 eligible patients aged 60+<br>Veteran, trauma or emergency care setting<br>Non-OECD country<br>Non-human participants<br>Palliative care<br>Non-ICU patients
**Focus**	
Patients aged 60+ who have undergone ICU	Neurological ICU patients only<br>Cardiosurgical ICU patients only
Follow up of at least 3 months	No follow up/Follow up less than three months
At least one of the following comparison groups:<br>• Age-matched community controls<br>• Scores taken before ICU<br>• Younger ICU patients	No comparison group
QoL at follow up measured by patients (carers may help but cannot do assessment on their own)	QoL at follow up all measured by proxy (ie. doctors or carers)
**Data/Outcomes**	
Validated QoL measure (EQ-5D, SF-36, NHP, WHOQOLBREF, QLI or variants of these)	Non-validated QoL measure only (eg. a simple question of whether QoL improved)
QoL summary score reported in paper for both groups, or:<br>• Subscores can be used to calculate summary scores<br>• Study references data for age-matched control that is fully reported elsewhere	No eligible data on QoL (or insufficient data to calculate summary scores)<br>QoL not reported for both groups (regression analyses do not count)

For peer review only

For peer review only - http://bmjopen.bmj.com/site/about/guidelines.xhtml

Appendix: Disparity or Discrimination?
A systematic review of socio-demographic associations of insight

6. REVIEW SEARCH TERMS

6.1 MEDLINE

(("intensive care"[title/abstract] OR "critical care"[title/abstract] OR "critical illness"[title/abstract] OR "Respiratory Distress Syndrome"[title/abstract] OR "Sepsis"[title/abstract] OR intensive care[MeSH Terms] OR critical care[MeSH Terms] OR "critical illness"[MeSH Terms] OR "Sepsis"[MeSH Terms]))

AND (("elderly"[title/abstract] OR "older adult*"[title/abstract] OR "geriatr*"[title/abstract] OR "dement*"[title/abstract] OR "Alzheimer*"[title/abstract] OR "parkinson's disease"[title/abstract] OR elderly [MeSH Terms] OR older adult*[MeSH Terms] OR geriatr*[MeSH Terms] OR dement*[MeSH Terms] OR septugenaria*[All Fields] OR octogenaria*[All Fields] OR nonagenaria*[All Fields] OR "over 5*"[title/abstract] OR "over 6*"[title/abstract] OR "over 7*"[title/abstract] OR "over 8*"[title/abstract] OR "over 9*"[title/abstract] OR "over 5*"[title/abstract] OR "over 6*"[title/abstract] OR "over 7*"[title/abstract] OR "over 8*"[title/abstract] OR "over 9*"[title/abstract]))

AND (("quality of life"[title/abstract] OR "EuroQol*"[All Fields] OR "Nottingham Health Profile"[All Fields] OR "NHP*"[All Fields] OR "SF-36"[All Fields] OR "RAND-36*"[All Fields]))

Filters: English Language, Humans, 01/01/2000 to 23/04/2020

6.2 Cochrane Database for Systematic Reviews & Cochrane Controlled Register of Trials (CENTRAL)

#1 ("intensive care" OR "critical care" OR "critical illness" OR "Respiratory Distress Syndrome" OR "Sepsis"):ti,ab,kw
#2 ("elderly" OR "older adult*" OR "geriatr*" OR "dement*" OR "Alzheimer*" OR "parkinson's disease"):ti,ab,kw
#3 (critical care OR critical illness OR Sepsis)
#4 (Aged OR geriatrics OR dementia)
#5 ("quality of life")
#6 ("EuroQol" OR "Nottingham Health Profile" OR "NHP" OR "SF-36" OR "RAND-36")
#7 MeSH descriptor: [Aged]
#8 MeSH descriptor: [Geriatrics]
#9 MeSH descriptor: [Dementia]
#10 MeSH descriptor: [Critical Care]
#11 MeSH descriptor: [Critical Illness]
#12 MeSH descriptor: [Sepsis]
#13 #1 OR #3 OR #10 OR #11 OR #12
#14 #2 OR #4 OR #7 OR #8 OR #9
#15 #5 AND #6
#16 #13 AND #14 AND #15= 124 (78 reviews, 36 trials).

6.3 Web of Science

Indexes = SCI-EXPANDED, SSCI, CPCI-S, CPCI-SHH, ESCI. **LANGUAGE** = English, **DOCUMENT TYPES** = (Article OR Abstract of Published Item), Timespan = All years (2000-2020)

#1 ALL=("intensive care" OR "critical care" OR "critical illness" OR "Respiratory Distress Syndrome" OR "Sepsis" OR "ICU")
#2 ALL=("elderly" OR "older adult*" OR "geriatr*" OR "dement*" OR "Alzheimer*" OR "parkinson's disease")
#3 ALL= ("quality of life" OR "EuroQol" OR "Nottingham Health Profile" OR "NHP" OR "SF-36" OR "RAND-36")
#4 #1 AND #2 AND #3
#5 #4 AND **LANGUAGE**: (English) AND **DOCUMENT TYPES**: (Article OR Abstract of Published Item) AND **Timespan**= 2000-2020

For peer review only - http://bmjopen.bmj.com/site/about/guidelines.xhtml

Appendix: Disparity or Discrimination?
A systematic review of socio-demographic associations of insight

6.4 EMBASE (& EMBASE Classic)

Dates: 2000-2020, Limits: Human participants only, English language, Articles only

#1 All Field: "intensive care" or "critical care" or "critical illness" or "Respiratory Distress Syndrome" or Sepsis or "ICU"
#2 Text Word: elderly or "older adult*" or "geriatr*" or "dement*" or "Alzheimer*" or "parkinson*"
#3 All Field: "quality of life" or EuroQol or Nottingham Health Profile or NHP or SF-36 OR RAND-36

6.5 CINAHL

Limits: English language only, Human participants, All adult, Peer-reviewed, Jan 2000 – April 2020

#1 TX: "intensive care" or "critical care" or "critical illness" or "Respiratory Distress Syndrome" or Sepsis or "ICU"
#2: SU: "Intensive Care Units" or "Intensive Care Units or Neonatal" or "Critical Care Nursing" or "Respiratory Distress Syndrome" or Acute or "Neonatal Intensive Care Nursing" or "Critical Care or Critical Path" or "Canadian Association of Critical Care Nurses" or "British Association of Critical Care Nurses" or "ventilator patients"
#3: TX: elderly or "older adult*" or "geriatr*" or "dement*" or "Alzheimer*" or "parkinson*"
#4: SU: "Older Adult Care (Saba CCC)" or "Frail Elderly" or "elderly patients" or "ventilator patients"
#5: TX: "quality of life" or EuroQol or "Nottingham Health Profile" or NHP or SF-36 OR RAND-36
#6: (S1 OR S2) AND (S3 OR S4) AND S5

6.6 PsycINFO

Limits: Date filter (2000-2020), English language, Human participants, Peer Reviewed Journal

#1 All Fields: "intensive care" or "critical care" or "critical illness" or "Respiratory Distress Syndrome" or Sepsis or "ICU"
#2 Text Word: elderly or "older adult*" or "geriatr*" or "dement*" or "Alzheimer*" or "parkinson*"
#3 All Fields: "quality of life" or EuroQol or Nottingham Health Profile or NHP or SF-36 OR RAND-36

For peer review only

PRISMA 2009 Checklist

Section/topic	#	Checklist item	Reported on page #
**TITLE**			
Title	1	Identify the report as a systematic review, meta-analysis, or both.	1
**ABSTRACT**			
Structured summary	2	Provide a structured summary including, as applicable: background; objectives; data sources; study eligibility criteria, participants, and interventions; study appraisal and synthesis methods; results; limitations; conclusions and implications of key findings; systematic review registration number.	1
**INTRODUCTION**			
Rationale	3	Describe the rationale for the review in the context of what is already known.	3
Objectives	4	Provide an explicit statement of questions being addressed with reference to participants, interventions, comparisons, outcomes, and study design (PICOS).	3
**METHODS**			
Protocol and registration	5	Indicate if a review protocol exists, if and where it can be accessed (e.g., Web address), and, if available, provide registration information including registration number.	1 & 4
Eligibility criteria	6	Specify study characteristics (e.g., PICOS, length of follow-up) and report characteristics (e.g., years considered, language, publication status) used as criteria for eligibility, giving rationale.	4, 8 & Appendix
Information sources	7	Describe all information sources (e.g., databases with dates of coverage, contact with study authors to identify additional studies) in the search and date last searched.	4 & Appendix
Search	8	Present full electronic search strategy for at least one database, including any limits used, such that it could be repeated.	4 & Appendix
Study selection	9	State the process for selecting studies (i.e., screening, eligibility, included in systematic review, and, if applicable, included in the meta-analysis).	4-6 & Appendix
Data collection process	10	Describe method of data extraction from reports (e.g., piloted forms, independently, in duplicate) and any processes for obtaining and confirming data from investigators.	4-5 & Appendix
Data items	11	List and define all variables for which data were sought (e.g., PICOS, funding sources) and any assumptions and simplifications made.	4 & Appendix
Risk of bias in individual studies	12	Describe methods used for assessing risk of bias of individual studies (including specification of whether this was done at the study or outcome level), and how this information is to be used in any data synthesis.	5, 9, 10 and Appendix
Summary measures	13	State the principal summary measures (e.g., risk ratio, difference in means).	5

For peer review only - http://bmjopen.bmj.com/site/about/guidelines.xhtml

PRISMA 2009 Checklist

Synthesis of results	14	Describe the methods of handling data and combining results of studies, if done, including measures of consistency (e.g., I^2) for each meta-analysis.	5

Section/topic	#	Checklist item	Reported on page #
Risk of bias across studies	15	Specify any assessment of risk of bias that may affect the cumulative evidence (e.g., publication bias, selective reporting within studies).	5, 9, 10 and Appendix
Additional analyses	16	Describe methods of additional analyses (e.g., sensitivity or subgroup analyses, meta-regression), if done, indicating which were pre-specified.	5
**RESULTS**			
Study selection	17	Give numbers of studies screened, assessed for eligibility, and included in the review, with reasons for exclusions at each stage, ideally with a flow diagram.	6
Study characteristics	18	For each study, present characteristics for which data were extracted (e.g., study size, PICOS, follow-up period) and provide the citations.	8 & Appendix
Risk of bias within studies	19	Present data on risk of bias of each study and, if available, any outcome level assessment (see item 12).	8-10 & Appendix
Results of individual studies	20	For all outcomes considered (benefits or harms), present, for each study: (a) simple summary data for each intervention group (b) effect estimates and confidence intervals, ideally with a forest plot.	9 & Appendix
Synthesis of results	21	Present results of each meta-analysis done, including confidence intervals and measures of consistency.	9 & Appendix
Risk of bias across studies	22	Present results of any assessment of risk of bias across studies (see Item 15).	9-10 & Appendix
Additional analysis	23	Give results of additional analyses, if done (e.g., sensitivity or subgroup analyses, meta-regression [see Item 16]).	9-10 & Appendix
**DISCUSSION**			
Summary of evidence	24	Summarize the main findings including the strength of evidence for each main outcome; consider their relevance to key groups (e.g., healthcare providers, users, and policy makers).	12
Limitations	25	Discuss limitations at study and outcome level (e.g., risk of bias), and at review-level (e.g., incomplete retrieval of identified research, reporting bias).	13-14
Conclusions	26	Provide a general interpretation of the results in the context of other evidence, and implications for future research.	13, 15

For peer review only

For peer review only - http://bmjopen.bmj.com/site/about/guidelines.xhtml

PRISMA 2009 Checklist

FUNDING			
Funding	27	Describe sources of funding for the systematic review and other support (e.g., supply of data); role of funders for the systematic review.	15

From: Moher D, Liberati A, Tetzlaff J, Altman DG, The PRISMA Group (2009). Preferred Reporting Items for Systematic Reviews and Meta-Analyses: The PRISMA Statement. PLoS Med 6(7): e1000097. doi:10.1371/journal.pmed1000097

For more information, visit: **www.prisma-statement.org**.

For peer review only

For peer review only - http://bmjopen.bmj.com/site/about/guidelines.xhtml

Quality of Life in elderly ICU survivors before the COVID-19 pandemic: A Systematic Review and Meta-Analysis of Cohort Studies.

Journal:	*BMJ Open*
Manuscript ID	bmjopen-2020-045086.R2
Article Type:	Original research
Date Submitted by the Author:	03-Jul-2021
Complete List of Authors:	Ariyo, Kevin; King's College London Department of Psychological Medicine<br>Canestrini, Sergio; King's College Hospital NHS Foundation Trust, Critical Care<br>David, Anthony; UCL, Institute of Mental Health<br>Ruck Keene, Alex; King's College London Department of Psychological Medicine; King's College London Dickson Poon School of Law<br>Wolfrum, Sebastian; University Hospital Schleswig Holstein, Medical Clinic II, Cardiology/Angiology/Intensive Care Medicine; University Hospital Schleswig Holstein, Department of Emergency Medicine<br>Owen, Gareth; King's College London Department of Psychological Medicine
**Primary Subject Heading**:	Intensive care
Secondary Subject Heading:	Epidemiology, Ethics, Health policy, Mental health, Public health
Keywords:	COVID-19, INTENSIVE & CRITICAL CARE, STATISTICS & RESEARCH METHODS, ETHICS (see Medical Ethics), Rationing < HEALTH SERVICES ADMINISTRATION & MANAGEMENT

SCHOLARONE™ Manuscripts

BMJ

I, the Submitting Author has the right to grant and does grant on behalf of all authors of the Work (as defined in the below author licence), an exclusive licence and/or a non-exclusive licence for contributions from authors who are: i) UK Crown employees; ii) where BMJ has agreed a CC-BY licence shall apply, and/or iii) in accordance with the terms applicable for US Federal Government officers or employees acting as part of their official duties; on a worldwide, perpetual, irrevocable, royalty-free basis to BMJ Publishing Group Ltd ("BMJ") its licensees and where the relevant Journal is co-owned by BMJ to the co-owners of the Journal, to publish the Work in this journal and any other BMJ products and to exploit all rights, as set out in our licence.

The Submitting Author accepts and understands that any supply made under these terms is made by BMJ to the Submitting Author unless you are acting as an employee on behalf of your employer or a postgraduate student of an affiliated institution which is paying any applicable article publishing charge ("APC") for Open Access articles. Where the Submitting Author wishes to make the Work available on an Open Access basis (and intends to pay the relevant APC), the terms of reuse of such Open Access shall be governed by a Creative Commons licence – details of these licences and which Creative Commons licence will apply to this Work are set out in our licence referred to above.

Other than as permitted in any relevant BMJ Author's Self Archiving Policies, I confirm this Work has not been accepted for publication elsewhere, is not being considered for publication elsewhere and does not duplicate material already published. I confirm all authors consent to publication of this Work and authorise the granting of this licence.

For peer review only

Quality of Life in elderly ICU survivors before the COVID-19 pandemic: A Systematic Review and Meta-Analysis of Cohort Studies.

ABSTRACT

Kevin Ariyo [1], Sergio Canestrini [2,3] , Anthony S. David [4], Alex Ruck Keene [1,5], Sebastian Wolfrum [6,7], Gareth S. Owen [1].

1. Department of Psychological Medicine, Institute of Psychiatry, Psychology and Neuroscience, King's College London, UK
2. Department of Critical Care, King's College Hospital, Denmark Hill, London, UK
3. Centre for Social Ethics and Policy, Department of Law, School of Social Science, University of Manchester, UK
4. UCL Institute of Mental Health, Division of Psychiatry, University College London, UK
5. Dickson Poon School of Law, King's College London, UK
6. Medical Clinic II, Cardiology/Angiology/Intensive Care Medicine, University Hospital Schleswig-Holstein, Germany
7. Department of Emergency Medicine, University Hospital Schleswig-Holstein, Germany

OBJECTIVES

The influence of age upon intensive care unit (ICU) decision-making is complex and it is unclear if it is based on expected subjective or objective patient outcomes. To address recent concerns over age-based ICU decision-making we explored patient-assessed quality of life (QoL) in ICU survivors before the COVID-19 pandemic.

DESIGN

A systematic review and meta-analysis of cohort studies published between January 2000 to April 2020, of elderly patients admitted to ICUs.

PRIMARY AND SECONDARY OUTCOME MEASURES

We extracted data on self-reported QoL (EQ-5D composite score), demographic and clinical variables. Using a random-effects meta-analysis, we then compared QoL scores at follow-up to scores either before admission, age-matched population controls or younger ICU survivors. We conducted sensitivity analyses to study heterogeneity and bias, and a qualitative synthesis of subscores.

RESULTS

We identified 2536 studies and included 22 for qualitative synthesis and 18 for meta-analysis (N= 2326 elderly survivors). Elderly survivors' QoL was significantly worse than younger ICU survivors, with a small-to-medium effect size (d= .35 [-.53, -.16]). Elderly survivors' QoL was also significantly greater when measured slightly before ICU, compared to follow-up, with a small effect size (d= .26 [-.44, -.08]). Finally, their QoL was also marginally significantly worse than age-matched community controls, also with a small effect size (d= .21 [-.43, .00]). Mortality rates and length of follow up partly explained heterogeneity. Reductions in QoL seemed primarily due to physical health, rather than mental health items.

CONCLUSIONS

The results suggest that the proportionality of age as a determinant of ICU resource allocation should be kept under close review and that subjective QoL outcomes should inform person-centred decision making in elderly ICU patients.

For peer review only - http://bmjopen.bmj.com/site/about/guidelines.xhtml

Quality of Life in elderly ICU survivors before the COVID-19 pandemic:
A Systematic Review and Meta-Analysis of Cohort Studies.

1

Strengths and limitations of this study

- To our knowledge, this is the first systematic review and meta-analysis to explore quality of life outcomes in elderly ICU survivors, and to explore sources of variation between these studies.
- To ensure consistency and policy relevance, we only included one type of measure within the meta-analysis (EQ-5D).
- With our large sample, we could estimate the population QoL with reasonable precision, as evidenced by narrow confidence intervals.
- Wide prediction intervals suggest that our results should not be used to make individual-level predictions.
- Our sample had a mixture of conditions, and because data was reported inconsistently and often at study-level, it is difficult to generalise to specific clinical groups, including COVID-19 patients.

For peer review only

For peer review only - http://bmjopen.bmj.com/site/about/guidelines.xhtml

Quality of Life in elderly ICU survivors before the COVID-19 pandemic:
A Systematic Review and Meta-Analysis of Cohort Studies.

INTRODUCTION

The influence that age should have upon intensive care decision making has been debated across policy and clinical practice [1 2]. Age associates (inversely) with the probability of intensive care unit (ICU) survival and length of life after ICU [3 4], outcomes generally considered to be relevant to resource allocation [2]. However, age is also a protected characteristic in several jurisdictions, and in England and Wales, resource allocation based on age must be a "proportionate means of achieving a legitimate aim", if it is not to be contrary to the Equality Act (2010).

For elderly patients for whom admission to ICU is clinically appropriate, an important part of person-centred decision-making is for them, or their families, to be given information about the likely outcome of admission. Patients may seek to integrate survival and biomedical outcomes with subjective outcomes, including quality of life (QoL). Subjective QoL in elderly ICU survivors has been studied less frequently than these objective measures [3 5]. This is notable given that subjective QoL (via Quality-Adjusted Life Years, or QALYs) is very influential in clinical resource allocation (e.g. NICE). Person-centred decision making requires consideration of patient experience since physician-rated quality of life is not always well correlated with patient-rated quality of life.

We considered a rapid review to be urgent because age is a strong risk factor for severe COVID-19 infection [6] and severe COVID-19 has placed considerable pressure on ICU resource allocation, [7] and is likely to do so in the future. Additionally, some have expressed concerns that elderly adults may have been disproportionately less likely to receive ICU before the pandemic [1 2 8-10]. As health system collapse remains a possibility, this raises the prospect of difficult triage decisions. In particular, services will need to weigh up various ethical positions to decide how important age is to these admission policies [11]. It is therefore important older persons' subjective outcomes are better understood.

We conducted a meta-analysis on patient reported QoL in elderly adults undergoing ICU. Following a systematic review, we addressed three questions:

1) At follow up, do elderly ICU survivors have better/worse QoL compared to their scores before ICU?
2) At follow up, do elderly ICU survivors have better/worse QoL than age-matched community controls?
3) At follow up, do elderly ICU survivors have better/worse QoL than ICU survivors aged under 65?

Determining the effect of illness and ICU on QoL is complicated because QoL is itself influenced by many variables [12] and some are non-clinical. These influences are too complex to resolve completely, but where possible, we sought to model relevant variables (illness severity, ICU length of stay and mortality rate) as predictors of QoL in elderly ICU survivors at follow up, compared to controls.

For peer review only - http://bmjopen.bmj.com/site/about/guidelines.xhtml

Quality of Life in elderly ICU survivors before the COVID-19 pandemic:
A Systematic Review and Meta-Analysis of Cohort Studies.

METHODS

SEARCH STRATEGY

We searched for English-language journal articles, published between January 2000 and April 2020. Six online bibliographic databases were used: CENTRAL, CINAHL, Cochrane Library, EMBASE, MEDLINE and PsycINFO. Our search followed a pre-published PROSPERO protocol (ID: CRD42020181181).

The search terms focused on intensive care, elderly adults and QoL (see item 6 of the Appendix). We supplemented this with a forward citations and reference list search based on the eligible articles as well as consultation with experts.

PATIENT AND PUBLIC INVOLVEMENT

No patient or public advisers were involved in this project.

SELECTION CRITERIA

We undertook study selection using EndNote X9 using a standardised CRIB sheet. See Figure 1 for an overview. The inclusion and exclusion criteria are detailed further in item 6 of the Appendix.

At the title and abstract level, we identified potentially eligible studies that took place in an ICU and referred to either QoL life or elderly adults. Full texts were eligible if a) all participants underwent ICU; b) there were at least 20 elderly patients and controls; c) scores from a validated QoL scale were reported, for a group aged at least 60+, with at least 3 months follow up review; d) the follow up QoL scores were derived from the patient, rather than a professional; and e) the study reported QoL scores from the same scale for either the same patients before the ICU admission, age-matched community controls or ICU survivors aged under 65.

Where we could not include potentially eligible studies, due to poor reporting, we contacted study authors for unpublished data. We also considered whether to include studies that focused only on cardio or neuro-surgical patients, given the effects of the diagnostic heterogeneity that characterises the reference population of the studies included in our review (general ICU patients with various conditions). However, none of these studies met the other inclusion criteria.

K.A led the study selection at all stages and a post-doctoral research assistant conducted reliability checks for 50% of full text articles. We found nearly perfect inter-rater agreement, as measured by Cohen's kappa (k= .86) [13]. Queries were resolved by G.O.

DATA EXTRACTION

One reviewer (K.A) extracted relevant data from all eligible studies, recording this on a standardised spreadsheet. M.K. independently extracted data from 10% of eligible studies, to evaluate consistency. The primary outcome was the QoL composite scores. Secondary variables included demographics, QoL subscale scores, mortality (from ICU to follow up), illness severity (APACHE-II or SAPS-II), length of ICU stay, length of hospital stay, and

For peer review only - http://bmjopen.bmj.com/site/about/guidelines.xhtml

Quality of Life in elderly ICU survivors before the COVID-19 pandemic:
A Systematic Review and Meta-Analysis of Cohort Studies.

average follow up time. When one dataset was used for multiple studies, we included the study with the clearest data reporting.

To ensure consistency, we included only composite scores from the EuroQoL health related quality of life instrument (EQ-5D) within the meta-analysis. Where possible, we also converted the eight SF-36 subscales to an EQ-5D index score, using an established mapping algorithm. [14] The remaining studies were included within the qualitative synthesis only.

DATA ANALYSIS

We explored the effect of age on EQ-5D composite scores using random effects meta-analyses. KA conducted the analysis using R Statistics. We used the Restricted Maximum Likelihood (REML) method to calculate the effect sizes (Cohen's d), which were weighted by the inverse of the sampling variance: meaning that studies with higher variance contributed less to the summary effect size. We interpreted these effect sizes using conventional criteria as a guide (0.2 = small; 0.5 = medium; 0.8 = large) [15]. We then conducted sensitivity analyses for each meta-analysis to assess risk of bias at the study level, including heterogeneity (e.g. I^2 statistic), influential studies (e.g. Cook's distance), and publication bias (funnel plots and Egger's test).

To investigate the remaining heterogeneity, we then conducted two secondary analyses: a moderator analysis to explore variation within a specific predictor, and a random-effects meta-regression to explore relationships between multiple predictors.

We used several strategies to handle missing data. When the study only reported median values and interquartile ranges, we estimated the mean and standard deviation using conventional formulae [16 17]. When neither the standard deviation nor interquartile range was reported, we estimated the standard deviation using prognostic imputation [18]. This calculates the average of observed variances to estimate the missing standard deviation values. We excluded studies with missing data if these methods were inapplicable.

One reviewer (K.A) assessed the methodological rigour of the included studies using an 11-item quality checklist (three irrelevant items were excluded) [19]. The criteria were scored as either 2 (complete fulfilment), 1 (partial fulfilment) or 0 (not fulfilled). We then calculated a total score for each study and rated them as either high quality (17/22 or higher), moderate quality (between 10/22 and 16/22) or low quality (9/22 or lower). Queries were resolved through discussion with G.O and S.C.

For the qualitative synthesis, we defined a set of criteria for each measure to allocate subscores to either 'mental health' or 'physical health' categories. We then calculated a crude average for subscales within these two categories and weighted them on a scale of 1-100 (0= minimum QoL; 100 = maximum QoL). As this approach is subjective, we present these findings only as a qualitative supplement.

This study follows methodological guidance from PRISMA (see appendix).

<Figure 1>

For peer review only - http://bmjopen.bmj.com/site/about/guidelines.xhtml

Quality of Life in elderly ICU survivors before the COVID-19 pandemic:
A Systematic Review and Meta-Analysis of Cohort Studies.

Figure 1. A PRISMA flow diagram that outlines the study selection process.

For peer review only

For peer review only - http://bmjopen.bmj.com/site/about/guidelines.xhtml

Quality of Life in elderly ICU survivors before the COVID-19 pandemic:
A Systematic Review and Meta-Analysis of Cohort Studies.

RESULTS

DESCRIPTIVE STATISTICS

After screening duplicates, the database search revealed 2536 records for title and abstract screening. From these, we reviewed 421 potentially relevant full text articles for eligibility. 16 of these studies met the full criteria and were included in the initial meta-analysis . A further two studies were deemed eligible following a forward citation search and contact with study authors . This led to a total of 18 studies included in the initial meta-analysis (n= 2326 elderly adults). Eleven of these studies reported age characteristics for the elderly patients (Mean= 79.04), while the others reported the minimum age only.

Most of the studies included both medical and surgical ICU patients (15 studies). The remaining studies focused on surgical (two studies) or medical (one study) patients only. A full breakdown of reasons for admissions is available in the appendix.

Three types of outcome were included in the meta-analysis. These results compared QoL at follow up to either pre-ICU scores (five studies), age-matched community controls (ten studies), or younger survivors of ICU (six studies). We provide a full summary in Table 1.

For the qualitative analysis, we identified four further studies . Five different measurement scales were reported: the EuroQoL EQ-5D health related quality of life instrument (EQ-5D utility index or visual analogue scale; eleven studies), the short form medical outcome questionnaire (SF-36; eight studies), the Nottingham health profile (NHP; one study), the quality of life index (QLI; one study) and the World Health Organisation quality of life instruments (WHO-QOL-BREF; one study). SF-36 scores were converted to EQ-5D index scores for the meta-analysis, while the other measures were excluded (see methods).

For peer review only - http://bmjopen.bmj.com/site/about/guidelines.xhtml

Quality of Life in elderly ICU survivors before the COVID-19 pandemic:
A Systematic Review and Meta-Analysis of Cohort Studies.

First Author	Year	Country	N	Min Age	% Male	Follow-up (avg. months)	ICU LoS (days)	Mortality	Severity (scaled avg.)	Raw Measure	Comparison	Quality
Abelha	2007	Portugal	112	65+	61.00%	6		28.00%		SF-36 *	ICU survivors younger than 65 years old	M
Ali	2018	Australia	32	65+	80.00% [a]	12	5		.24	EQ-5D	Age-matched South Australian controls	H
Andersen	2015	Norway	53	80+	69.00%	40.8	1.9	81.52%	.27	EQ-5D	Age and sex-matched Norwegian population	M
De Rooij	2008	Netherlands	187	80+	51.00%	44.4	1.29	61.52%	.21	EQ-5D	Age-matched British population	M
Eddleston	2000	UK	39	65+	52.45% [a]	3				SF-36 *	ICU survivors younger than 65 years old	M
Ferrao	2015	Portugal	290	66+ [b]	26.00%	27.6				EQ-5D	ICU survivors younger than 65 years old	M
Grace	2007	Australia/NZ	99	60+	NR	28		60.00%	.28	EQ-5D	Retrospective patient ratings for one week before ICU	L
Hofhuis	2011	Netherlands	49	80+ [b]	46.90%	6	5.35	40.83%	.25	SF-36 *	Age-matched Dutch population and Retrospective proxy ratings for four weeks before ICU	M
Honselmann [c]	2015	Germany	352	65+	53.40%	12	2.58	43.36%		EQ-5D	ICU survivors younger than 65 years old	
Honselmann [c-d]	2015	Germany	291	65+	53.61%	12	2.34	43.36%		EQ-5D	Age-matched German controls	
Jeitziner	2015	Switzerland	124	65+	73.00%	12	4.57		.29	SF-36 *	Age matched Swiss controls and Retrospective patient ratings for one week before ICU	M
Kaarola	2006	Finland	299	65+	75.00%	47		57.00%		EQ-5D	ICU survivors younger than 65 years old	M
Levinson	2016	Australia	322	80+	58.00% [a]	24	1.28	21.45%		SF-36 *	Age and sex-matched Australian population	H
Merlani	2007	Switzerland	36	70+	52.00%	24	3.00	63.00%	.26	EQ-5D	Age-matched Swiss population	M
Oeyen	2007	Netherlands	63	80+	60.00% [a]	12		49.60%	.26	EQ-5D	Retrospective patient or proxy ratings for one week before ICU	M
Sacanella	2011	Spain	112	65+	57.00%	12	3.35	48.70%	.27	EQ-5D	Retrospective patient or proxy ratings before feeling ill and requiring ICU	M
Schroder	2011	Denmark	36	75+	56.00%	12	9.4	53.85%		SF-36 *	Age-matched Danish population	L
Sznajer	2001	France	65	65+ [b]	55.90% [a]	6				EQ-5D	ICU survivors younger than 65 years old	M
Villa	2016	Spain	54	75+	50.00%	12		43.18%	.23	SF-36 *	Spanish population aged 75+	M
Weighted avg.			*128.53*	*69.50*	*55.74%*	*22.98*	*3.02*	*44.92%*	*.26*			
Range			*23-352*	*60-80*	*26-80%*	*3-100.8*	*1.28-9.4*	*21.45-81.52%*	*.12-.34*			

Table 1. The main characteristics of the studies and the relevant data included in the meta-analyses.
[a] Reported for study level only, so not included in meta-analysis
[b] Combined elderly groups.
[c] We analysed some unpublished data from Honselmann et al, therefore we have presented descriptives for the full dataset only, without quality assessment.
[d] In the Honselmann study, the sample for the community study was slightly smaller than for the young/old comparison.
[*] Converted to EQ-5D composite score.
Abbreviations: ICU (intensive care unit); LoS (length of stay); H = High quality; M= Moderate quality; L= Low quality. See above for measures.
Unless specified, we do not report data where it is not representative of at least 66.67% of the included sample.

For peer review only

For peer review only - http://bmjopen.bmj.com/site/about/guidelines.xhtml

Quality of Life in Older Adults following Intensive Care:
A Rapid Systematic Review of Cohort Studies.

META ANALYSES

Comparison	k	Cohen d	95% CI	95% PI	P	I2
Pre-ICU scores	5	-.26	-.44, -.08	-.58, .07	.005	45.50%
Community	10	-.22	-.43, .00	-.88, .45	.053	87.88%
Under 65s	6	-.35	-.53, -.16	-.83, .18	.000	81.93%

Table 2. A summary of effect sizes, confidence intervals, prediction intervals, significance and heterogeneity for each meta-analysis (k= number of independent samples, I^2= between study heterogeneity)

Table 2 outlines the results of the three meta-analyses.

There was a significant difference in EQ-5D composite scores between elderly patients before and after ICU, with a small effect size (d= -26, p= .005). This suggests that elderly patients may expect a slightly worse QoL at follow up, compared to their own scores one month before ICU.

There was a marginally significant difference in EQ-5D composite scores between elderly ICU survivors and age-matched community controls, with a small effect size (d= -.22, p= .05). These results suggest that QoL may be slightly lower in elderly ICU survivors, relative to community controls.

Elderly ICU survivors (aged over 65) had significantly lower composite scores on the EQ-5D, compared to younger ICU survivors (aged under 65), with a small-to-medium effect size (d= -.33, p <.01). This suggests that on average, QoL in elderly ICU survivors is slightly worse than younger ICU survivors.

SENSITIVITY ANALYSES

We reviewed the impact of influential cases within each analysis. One study was excluded from the community meta-analysis as a substantial outlier and influential result . If the result had not been excluded, the effect size would have been stronger (d= -1.97 – ie a larger difference in QoL favouring younger controls) but non-significant (p= .27), mainly due to large heterogeneity (I^2 = 100%). It is unclear why this study reported substantially outlying results, although the reported standard deviations were considerably lower than other studies.

After excluding this, one other study was somewhat influential within the community analysis (see Appendix) . This study was retained as we acquired the full dataset and we can therefore be confident of its reporting accuracy. If this study was excluded, the effect size would have been weaker (d= -.13) and non-significant (.010) in the same direction.

We identified no further outliers according to our criteria.

SECONDARY ANALYSES

There was moderate-to-large heterogeneity between studies. For significant results, we explored the role of other variables using post-hoc subgroup analyses and meta-regressions. These results should be interpreted with caution, due to low sample sizes.

For peer review only - http://bmjopen.bmj.com/site/about/guidelines.xhtml

Quality of Life in Older Adults following Intensive Care:
A Rapid Systematic Review of Cohort Studies.

Length of follow up significantly predicted greater differences in QoL between elderly ICU survivors and patients aged under 65 (k= 6, p< .001). This suggests that elderly survivors may have worse QoL in the long term and comparable QoL in the medium term.

The minimum age of the sample significantly predicted greater differences in QoL between elderly ICU survivors and age-matched community controls (k= 10, p= .02). Subgroup analyses revealed that in studies with only very old patients (aged 75-80+), elderly ICU survivors' QoL was no worse than their age-matched community controls (k= 6, d= -.06, p> .05). In contrast, when elderly was defined as 65-70+, elderly ICU survivors had much worse QoL than age-matched community controls (k= 4, d= .45, p< .03). This suggests that some of the variation was due to age differences in QoL in community controls.

Controlling for these variables reduced heterogeneity between studies by 10% and 47%, in both cases. No model significantly accounted for variance when the outlier was included in the community analysis.

Neither severity of illness, year of publication nor sex significantly accounted for heterogeneity between the studies, either individually or within a meta-regression (p> .05).

RISK OF BIAS

We found no evidence for publication bias for the community or pre-ICU meta-analyses, from either funnel plots or Egger's test (all p> .05). Most studies had a moderate degree of methodological quality (13/17). We had insufficient power to explore the effect of study quality on quantitative outcomes.

QUALITATIVE SYNTHESIS

To compare different aspects of QoL, we categorised the subscales into either mental or physical health QoL and calculated a scaled average to enable comparisons (see Table 3). 16/22 studies reported the subscales for both conditions. Our estimates suggest that elderly ICU survivors reported higher average scores on mental health items (Mean= 57.08/100) than physical health items (Mean= 47.12/100). Trends in physical health scores compared less favourably to age-matched community controls than did mental health scores (mean differences = -5.23 and -1.71, respectively). Trends in physical health scores were also lower in comparison to younger ICU controls (mean difference = -2.63) whereas mental health scores were higher (mean difference = 2.65).

For peer review only - http://bmjopen.bmj.com/site/about/guidelines.xhtml

Quality of Life in Older Adults following Intensive Care:
A Rapid Systematic Review of Cohort Studies.

First Author	Comparison	Measure	Mean MH (Elder ICU survivor)	Mean MH (Comparison)	Mean Difference	Mean PH Score (Elder ICU survivor)	Mean PH (Comparison)	Mean Difference
Anderson	Community	EQ5D	58.62	55.87	2.75	47.27	48.46	-1.19
De Rooij	Community	EQ5D	56.86	58.22	-1.35	48.89	50.49	-1.60
Merlani	Community	SF36	43.00	47.00	-4.00	36.00	42.00	-6.00
Jeitziner	Community	SF36	69.72	80.37	-10.65	62.71	77.91	-15.20
Villa	Community	SF36	62.40	61.50	0.90	66.60	67.90	-1.30
Garrouste-Orgeas	Community	NHP	67.13	83.00	-15.87	53.63	70.23	-16.60
Schroder	Community	SF36	56.93	54.30	2.64	38.36	43.71	-5.35
Tabah	Community	WHOQOL	73.30	61.40	11.90	62.10	56.70	5.40
Average	*Community*		*61.00*	*62.71*	*-1.71*	*51.94*	*57.18*	*-5.23*
Grace	PreICU	EQ5D	50.80	51.40	-0.60	36.30	36.90	-0.60
Cuthbertson	PreICU	SF36	54.00	61.67	-7.67	53.22	58.50	-5.28
Hofhuis	PreICU	SF36	51.20	50.10	1.10	38.60	38.80	-0.20
Jeitziner	PreICU	SF36	69.72	69.02	0.70	62.71	63.63	-0.92
Average	*PreICU*		*56.43*	*58.05*	*-1.62*	*47.71*	*49.46*	*-1.75*
Abelha	Young	SF36	48.50	47.50	1.00	46.50	48.50	-2.00
Cuthbertson	Young	SF36	51.40	51.30	0.10	37.30	37.50	-0.20
Hofhuis	Young	SF36	51.20	50.40	0.80	38.60	38.70	-0.10
Honselmann	Young	EQ-5D	51.67	51.00	0.67	44.00	54.00	-10.00
Schroder	Young	SF36	56.93	54.30	2.64	38.36	43.71	-5.35
Eddleston	Young	SF36	63.59	58.58	5.01	58.76	63.25	-4.49
Kleinpell	Young	QLI	76.26	67.93	8.32	66.33	62.60	3.73
Average	*Young*		*57.08*	*54.43*	*2.65*	*47.12*	*49.75*	*-2.63*

Table 3. An overview of Quality of Life subscores, by mental health and physical health categories, for elderly ICU survivors and comparison groups. All scores were recalculated on a 0-100 (0 = minimum QoL; 100 = maximum QoL).
Abbreviations: MH= Mental Health; PH=Physical Health

For peer review only

Quality of Life in Older Adults following Intensive Care:
A Rapid Systematic Review of Cohort Studies.

DISCUSSION

This review has systematically evaluated the literature on QoL for elderly ICU survivors in the medium to long term, using EQ-5D composite scores. To our knowledge this is the first meta-analysis to address this issue. We found evidence that elderly patients who survive ICU can be expected to have slightly worse QoL, compared to younger survivors. To a lesser extent, they may also have worse QoL compared to age-matched community controls and compared to their own QoL up to one month before ICU. The wide prediction intervals also suggest that age differences can vary considerably in either direction.

STRENGTHS IN RELATION TO THE LITERATURE

For the meta-analysis, we identified 2326 elderly ICU survivors within an international sample of 18 cohort studies. We only included recent studies that used validated QoL measures and we rated most studies as having moderate or higher methodological quality. By pooling these samples using rigorous methods, we have been able to overcome several methodological limitations associated with generalising from individual studies, including small samples, choice of analysis and site selection bias. Our sensitivity analyses showed that the remaining heterogeneity was partly due to conceptually relevant variables. Given the relatively small literature, these methods ensure that valid, transparent results inform policy and clinical practice decisions.

Although contested, previous reviews have generally concluded that age alone is not a suitable determinant of potential benefit from ICU, especially for survivors [3 5 20 21] . The present study supports these conclusions, although the differences compared to younger ICU survivors (and to a lesser extent, community samples) are still noteworthy. Decisions on whether to admit patients can be extremely difficult for all involved, with seriously ill elderly people overrepresented among the most contentious cases [22]. These challenges are amplified further when healthcare resources are under pressure, such as during the COVID-19 pandemic.

The age-QoL associations we have found may be explained by intermediary variables. Some research suggests that frailty may best explain age differences in QoL following ICU [5 23], and clinical outcome in COVID-19 patients [24]. Frailty is a more integrative approach to conceptualising ageing, but it was not reported within the eligible studies. We would also recommend a meta-analysis of individual patient data for COVID-19 patients, to further stratify clinical variables of interest, including frailty, and to better predict QoL outcomes.

Health economic analysis of ICU in the elderly based on QALYs may be informative when it comes to resource allocation policies, but we have found few such analyses and no explicit polices based on them. They will have to grapple with the controversial notion that everyone is entitled to a 'normal' span of health or 'a fair innings' [25 26]. Given the presumption that a sizeable proportion of elderly survivors will enjoy a good QoL it is crucial that holistic, person-centred decision making is not crowded out by survival statistics or anticipatory triage. If triage were to become necessary on the front line, we would advise against weighing age too heavily and rather taking more account of frailty after appropriate consultations.

On average, QoL scores gradually decline with age at approximately 0.5 points per year on the CASP-19 (range 0-57) with a modestly accelerated decrease with older age (>85 years) [4].

For peer review only - http://bmjopen.bmj.com/site/about/guidelines.xhtml

Quality of Life in Older Adults following Intensive Care:
A Rapid Systematic Review of Cohort Studies.

It is relevant to consider whether change in QoL in the elderly is primarily due to physical health and mental health components. We were unable to incorporate physical and mental subscores into the meta-analysis due to differences in the levels of data between measures, so we performed a qualitative synthesis. This suggested that for elderly ICU survivors, mental health questionnaire items were relatively unaffected. The small literature on older adults also suggests relatively low rates of anxiety [27] and depressive disorders [28 29], although potentially high rates of post-traumatic stress. [30] Further mental health data is needed, as some preliminary reports suggest high rates of posttraumatic stress in COVID-19 ICU patients [31 32]. Our results may serve as a baseline to compare mental and physical health outcomes between COVID and non-COVID survivors.

LIMITATIONS

The primary limitation is the small number of eligible studies for each analysis. To maximise the sample, we included some studies with a small amount of missing data and used validated methods to estimate the mean or the standard deviation from the reported statistics. We argue that these approaches are justified as, based on central limit theorem, we expect the larger sample sizes to produce a better estimate of population variance [33]. For balance, we have also provided a comprehensive overview of our sensitivity analyses to assess risk of bias (see Appendix). These demonstrate that although our decisions reduced bias, most did not change our interpretation of the effects.

Another potential limitation of the meta-analysis is the focus on long-term ICU survivors, as reported mortality rates were as high as 80% at follow up. We argue that a substantial 'healthy survivor' effect on QoL is unlikely because survival and QoL have different pathophysiological determinants. We also did not find any evidence of better QoL for elderly patients in studies with high mortality rates. Nevertheless, our results clearly extend only to ICU survivors, rather than prospective ICU patients.

Our results may also be prone to other selection biases. Compared to younger adults, unhealthy elderly adults might be less likely to be admitted to into ICU [22 34], to survive ICU treatment (possibly in part due to decisions around lifesaving treatment [35]) and to survive until follow-up. It was also unclear how many patients had pre-existing cognitive impairments where QoL measurement is more complex, although there was no indication that the proportion was large. Without further data on contextual variables, we would caution wider generalisations to all elderly ICU patients. Nonetheless, these results imply that at least some elderly ICU patients will have a relatively good QoL in the medium-to-long term.

In particular, no COVID-19 patients were included in the sample. COVID-19 pneumonitis has a specific pathophysiology that does not lead to a 'typical' acute respiratory syndrome and this can require a relatively high degree of multi systemic involvement. Future studies will need to consider elderly COVID survivors, who often require a relatively lengthy period of ICU treatment and post-ICU rehabilitation, especially if unvaccinated.

We were unable to assess quality of life as rigorously as we would have liked. This was partly because studies varied in their definitions of 'old age'. Most of the eligible studies defined this as 65+, following the World Health Organisation definition [36]. However, patients aged 65+ currently account for roughly half of all ICU admissions [37]. It is therefore likely that a higher threshold would be more relevant to investigate age-related syndromes. A consensus

For peer review only - http://bmjopen.bmj.com/site/about/guidelines.xhtml

Quality of Life in Older Adults following Intensive Care:
A Rapid Systematic Review of Cohort Studies.

on what should count as ‘very old’ would help data collection, analysis and interpretation within this field.

The scores for pre-ICU scores were determined by retrospective ratings from discharged patients or proxies. This is usual practice, but the reliability of proxies is contested [38 39]. Ideally, we would have analysed differences in QoL change scores between younger and elderly ICU survivors, at multiple time points from before ICU to follow up.

Finally, we observed moderate-to-high levels of heterogeneity between studies, which limits the generalisability of the results. We found that much of this variation may have been due to mortality rates and length of time post-discharge, which supports the view that age alone is not a strong predictor of QoL outcome. We also tried to ensure consistency of measurement by using a mapping function between SF-36 scores to EQ-5D scores, which is a common approach within NICE guidelines [14 40].

For peer review only

For peer review only - http://bmjopen.bmj.com/site/about/guidelines.xhtml

Quality of Life in Older Adults following Intensive Care:
A Rapid Systematic Review of Cohort Studies.

CONCLUSION

Our study reports the first known meta-analysis of quality of life in elderly patients following ICU. We report that on average, elderly survivors of ICU have slightly worse QoL compared to younger ICU survivors, based on physical rather than mental health. To a lesser extent, they may also have worse QoL compared to their own scores before ICU and compared to their community peers. These findings add rigour to the current literature and should inform debates around population level resource allocation and person-centred intensive care decision making during the current COVID-19 pandemic and after.

CORRESPONDENCE

Mr. Kevin Ariyo MSc (PhD candidate), Mental Health and Justice Project, Department of Psychological Medicine, Institute of Psychiatry, Psychology and Neuroscience, King's College London. 16 De Crespigny Park, Camberwell, London, SE5 8AF. Email: kevin.ariyo@kcl.ac.uk.

AUTHOR STATEMENT

K.A. led at each stage of the project, including drafting the document. G.O. was primary supervisor on the project, jointly formulated the research questions, led on writing the introduction section and made substantial contributions to all aspects of the study. S.C. advised on the initial protocol and provided critical revisions from an intensivist perspective. A.D., A.R.K. and provided additional supervision and critical revisions. S.W. also contributed to data collection and analysis, by providing previously unpublished data, and critical revisions.

The manuscript is a transparent account of the study being reported and adheres to PRISMA reporting guidelines. All listed authors have approved for the manuscript to be published in its current format and meet all the ICMJE criteria for authorship. The authors agree to be accountable for the contents of the paper and are jointly responsible for ensuring that all queries related to the accuracy or integrity of the project are investigated and resolved.

ACKNOWLEDGEMENTS

We are grateful to Margot Kuylen for her contributions to the reliability assessment and to John Brazier for advising on the SF-36 to EQ-5D mapping function.

DECLARATIONS OF INTEREST

Alex Ruck Keene is an adviser on the Faculty of Intensive Care Medicine's Legal and Ethical Policy Unit. Authors have no other conflict of interests.

ETHICAL APPROVAL

Not required.

DATA SHARING

For peer review only - http://bmjopen.bmj.com/site/about/guidelines.xhtml

Quality of Life in Older Adults following Intensive Care:
A Rapid Systematic Review of Cohort Studies.

The datasets generated and analysed during the current study are included in this published article and its supplementary information files. Any data queries may also be directed to the corresponding author on reasonable request.

FUNDING

Supported by the Mental Health and Justice Project, led by G.O., which is funded by a grant from the Wellcome Trust (203376/2/16/Z).

For peer review only

Quality of Life in Older Adults following Intensive Care:
A Rapid Systematic Review of Cohort Studies.

REFERENCES

1. Archard D, Caplan A. Is it wrong to prioritise younger patients with covid-19? *BMJ* 2020;369
2. Savulescu J, Cameron J, Wilkinson D. Equality or utility? Ethics and law of rationing ventilators. *British Journal of Anaesthesia* 2020
3. Hennessy D, Juzwishin K, Yergens D, et al. Critical care review. Outcomes of elderly survivors of intensive care: a review of the literature. *CHEST* 2005;127(5):1764-74.
4. Zaninotto P, Falaschetti E, Sacker A. Age trajectories of quality of life among older adults: results from the English Longitudinal Study of Ageing. *Quality of Life Research* 2009;18(10):1301-09.
5. Conti M, Merlani P, Ricou B. Prognosis and quality of life of elderly patients after intensive care. *Swiss Med Wkly* 2012;142:w13671. doi: 10.4414/smw.2012.13671 [published Online First: 2012/09/12]
6. Zhou F, Yu T, Du R, et al. Clinical course and risk factors for mortality of adult inpatients with COVID-19 in Wuhan, China: a retrospective cohort study. *The lancet* 2020
7. Willan J, King AJ, Jeffery K, et al. Challenges for NHS hospitals during covid-19 epidemic: British Medical Journal Publishing Group, 2020.
8. de Castro-Hamoy L, de Castro LD. Age Matters but it should not be Used to Discriminate Against the Elderly in Allocating Scarce Resources in the Context of COVID-19. *Asian Bioethics Review* 2020:1-10.
9. Haas LE, de Lange DW, van Dijk D, et al. Should we deny ICU admission to the elderly? Ethical considerations in times of COVID-19. *Critical Care* 2020;24(1):1-3.
10. Montero-Odasso M, Hogan DB, Lam R, et al. Age alone is not adequate to determine healthcare resource allocation during the COVID-19 pandemic. *Canadian Geriatrics Journal* 2020;23(1):152-54.
11. Kuylen MN, Kim SY, Keene AR, et al. Should age matter in COVID-19 triage? A deliberative study. *Journal of Medical Ethics* 2021;47(5):291-95.
12. Rubenfeld GD. Does the hospital make you older faster?: American Thoracic Society, 2012.
13. Landis JR, Koch GG. The measurement of observer agreement for categorical data. *biometrics* 1977:159-74.
14. Ara R, Brazier J. Deriving an algorithm to convert the eight mean SF-36 dimension scores into a mean EQ-5D preference-based score from published studies (where patient level data are not available). *Value in Health* 2008;11(7):1131-43.
15. Cohen J. Statistical power analysis for the behavioral sciences New York. *NY: Academic* 1988
16. Luo D, Wan X, Liu J, et al. Optimally estimating the sample mean from the sample size, median, mid-range, and/or mid-quartile range. *Statistical methods in medical research* 2018;27(6):1785-805.
17. Wan X, Wang W, Liu J, et al. Estimating the sample mean and standard deviation from the sample size, median, range and/or interquartile range. *BMC medical research methodology* 2014;14(1):135.
18. Ma J, Liu W, Hunter A, et al. Performing meta-analysis with incomplete statistical information in clinical trials. *BMC medical research methodology* 2008;8(1):56.
19. Kmet LM, Cook LS, Lee RC. Standard quality assessment criteria for evaluating primary research papers from a variety of fields. 2004

For peer review only

Quality of Life in Older Adults following Intensive Care:
A Rapid Systematic Review of Cohort Studies.

20. Chelluri L, Grenvik A, Silverman M. Intensive care for critically ill elderly: mortality, costs, and quality of life: review of the literature. *Archives of Internal Medicine* 1995;155(10):1013-22.
21. Wehler M. [Long-term outcome of elderly patients after intensive care treatment]. *Med Klin Intensivmed Notfmed* 2011;106(1):29-33. doi: 10.1007/s00063-011-0021-x [published Online First: 2011/10/07]
22. Escher M, Cullati S, Hudelson P, et al. Admission to intensive care: A qualitative study of triage and its determinants. *Health Serv Res* 2019;54(2):474-83. doi: 10.1111/1475-6773.13076 [published Online First: 2018/10/27]
23. Heyland DK, Garland A, Bagshaw SM, et al. Recovery after critical illness in patients aged 80 years or older: a multi-center prospective observational cohort study. *Intensive care medicine* 2015;41(11):1911-20.
24. Hewitt J, Carter B, Vilches-Moraga A, et al. The effect of frailty on survival in patients with COVID-19 (COPE): a multicentre, European, observational cohort study. *The Lancet Public Health* 2020
25. NICE CITIZENS COUNCIL REPORT ON AGE: National Institute for Care Excellence, 2020.
26. Williams A. Intergenerational equity: an exploration of the 'fair innings' argument. *Health economics* 1997;6(2):117-32.
27. Jeitziner MM, Hamers JP, Bürgin R, et al. Long-term consequences of pain, anxiety and agitation for critically ill older patients after an intensive care unit stay. *Journal of clinical nursing* 2015;24(17-18):2419-28.
28. Broslawski G, Elkins M, Algus M. Functional abilities of elderly survivors of intensive care. *The Journal of the American Osteopathic Association* 1995;95(12):712.
29. Chelluri L, Pinsky MR, Donahoe MP, et al. Long-term Outcome of Critically III Elderly Patients Requiring Intensive Care. *Jama* 1993;269(24):3119-23.
30. Drews T, Franck M, Radtke FM, et al. Postoperative delirium is an independent risk factor for posttraumatic stress disorder in the elderly patient: a prospective observational study. *European Journal of Anaesthesiology (EJA)* 2015;32(3):147-51.
31. Antonis T. Post-Traumatic Stress Disorder (Ptsd) In Patients with Covid 19. *Ann Clin Med Case Rep* 2021:V5.
32. de Graaf M, Antoni M, Ter Kuile M, et al. Short-term outpatient follow-up of COVID-19 patients: A multidisciplinary approach. *EClinicalMedicine* 2021;32:100731.
33. Weir CJ, Butcher I, Assi V, et al. Dealing with missing standard deviation and mean values in meta-analysis of continuous outcomes: a systematic review. *BMC medical research methodology* 2018;18(1):25.
34. Garrouste-Orgeas M, Timsit JF, Montuclard L, et al. Decision-making process, outcome, and 1-year quality of life of octogenarians referred for intensive care unit admission. *Intensive Care Med* 2006;32(7):1045-51. doi: 10.1007/s00134-006-0169-7 [published Online First: 2006/06/23]
35. Ferrand E, Robert R, Ingrand P, et al. Withholding and withdrawal of life support in intensive-care units in France: a prospective survey. *The Lancet* 2001;357(9249):9-14.
36. BRUNDTLAND G. Achieving health across the life span. *Men Aging and Health* 1999
37. Boumendil A, Somme D, Garrouste-Orgeas M, et al. Should elderly patients be admitted to the intensive care unit? *Intensive Care Med* 2007;33(7):1252. doi: 10.1007/s00134-007-0621-3 [published Online First: 2007/04/04]
38. Capuzzo M, Grasselli C, Carrer S, et al. Quality of life before intensive care admission: agreement between patient and relative assessment. *Intensive care medicine* 2000;26(9):1288-95.

For peer review only - http://bmjopen.bmj.com/site/about/guidelines.xhtml

39. Dinglas VD, Gifford JM, Husain N, et al. Quality of life before intensive care using EQ-5D: patient versus proxy responses. *Critical care medicine* 2013;41(1):9.
40. Longworth L, Rowen D. Mapping to obtain EQ-5D utility values for use in NICE health technology assessments. *Value in health* 2013;16(1):202-10.

For peer review only

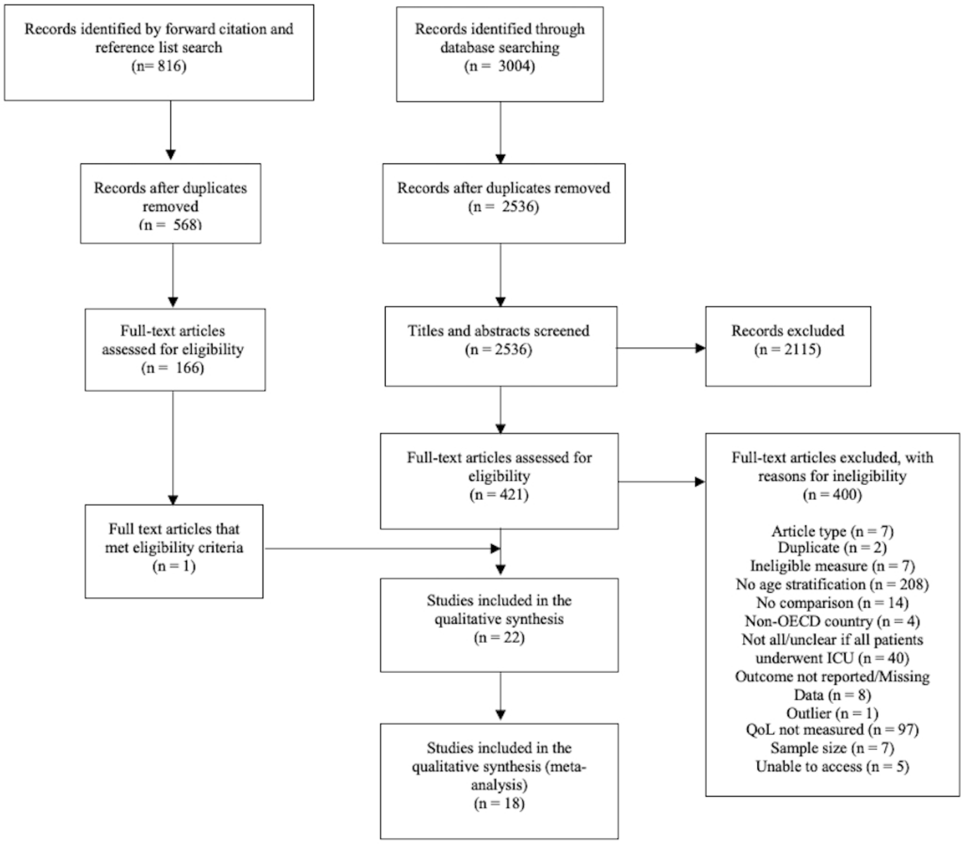

From: Moher D, Liberati A, Tetzlaff J, Altman DG, The PRISMA Group (2009). *P*referred *R*eporting *I*tems for *S*ystematic Reviews and *M*eta-

1. STUDY CHARACTERISTICS

1.1 Meta-Analysis

First Author	Year	Country	Study Design	Journal	Setting	Min Age	Avg. Age (SD)	% Male	Mortality	ICU LoS (SD)	HLoS (SD)	Severity	Raw Measure	Follow up	Comparison	Study Quality	Participant No.	Control No.	Effect Size	Variance
Abelha[39]	2007	Portugal	Cohort (unspecified)	BMC Anaesthesiology	Surgical ICU	65+		61.00%	28.00%				SF-36 [*]	6 months	ICU survivors younger than 65 years old	M	112	114	-.07	.02
Ali [38]	2018	Australia	Prospective Cohort	Journal of Critical Care	Medical-Surgical ICU	65+	73 (5)	80.00% [a]		4.64 (2.32)	16.29 (9.28)	.24	EQ-5D	12 months	Age-matched South Australian controls	H	32	572	.03	.03
Andersen [37]	2015	Norway	Retrospective Cohort	Annals of Intensive Care	General Hospital ICU	80+	87.4 (4)	69.00%	81.52%	1.9 (NR)		.27	EQ-5D	40.8 months	Age and sex-matched Norwegian population	M	53	170	-.18	.02
De Rooij [35]	2008	Netherlands	Retrospective Cohort	Journal of the American Geriatric Society	Medical-Surgical ICU	80+	81.7 (2.4)	51.00%	61.52%	1.29 (1.13)		.21	EQ-5D	44.4 months	Age-matched British population	M	187	142	-.24	.01
Eddleston [34]	2000	UK	Prospective Cohort	Critical Care Medicine	General Hospital ICU	65+		52.45%[a]					SF-36 [*]	3 months	ICU survivors younger than 65 years old	M	39	97	-.21	.04
Ferrao [33]	2015	Portugal	Retrospective Cohort	Critical Care	Medical-Surgical ICU	66+ [b]		26.00%					EQ-5D	27.6 months	ICU survivors younger than 65 years old	M	290	652	-.37	.01
Grace [31]	2007	Australia/NZ	Retrospective Cohort	Critical Care and Resuscitation	Mixed ICUs	60+		NR	60.00%			.28	EQ-5D	28 months	Retrospective patient ratings for one week before ICU	L	99	99	-.36	.02
Hofhuis [30]	2011	Netherlands	Prospective Cohort	Chest	Medical-Surgical ICU	80+ [b]	83 (3.06)	46.90%	40.83%	5.35 (2.29)	25.48 (16.04)	.25	SF-36 [*]	6 months	Age-matched Dutch population	M	49	49 [c]	.26	.04
															Retrospective proxy ratings for four weeks before ICU		49	49	.01	.04
Hosselmann	2015	Germany	Retrospective Cohort	Journal of Critical Care (part unpublished)	Mixed ICU (unpublished)	65+	75.84	53.00%	43.00%	2.58 (NR)			EQ-5D	12 months	ICU survivors younger than 65 years old	N/A (unpublished)	352	249	.90	.00
							75.16	54.00%	43.00%	2.34			EQ-5D	12 months	Age-matched German controls	N/A (unpublished)	291	828	.41	.00

For peer review only

[illegible]eitziner [29]	2015	Switzerland	Retrospective Cohort	Journal of Clinical Nursing	Medical-Surgical ICU	65+	68.72 (5.39)	73.00%		4.57 (5.81)		.29	SF-36 *	12 months	Age matched Swiss controls;	M	124	145	-.59	.02
															Retrospective patient ratings for one week before ICU		124	135	-.08	.01
Kaarola [28]	2006	Finland	Cross-Sectional	Critical Care Medicine	Medical-Surgical ICU	65+		75.00%	57.00%				EQ-5D	47 months	ICU survivors younger than 65 years old	M	299	800	-.67	.00
Levinson [26]	2016	Australia	Prospective Cohort	Internal Medicine Journal	Private ICU	80+	84.59 (NR)	58.00%[a]	21.45%	1.28 (NR)	12.91 (NR)		SF-36 *	24 months	Age and sex-matched Australian population	H	322	907	.04	.00
[illegible]erlani [25]	2007	Switzerland	Retrospective Cohort	Acta Anaesthesiologica Scandinavica	Surgical ICU	70+	78 (5)	52.00%	63.00%	3.00 (13.72)	22.50 (93.88)	.26	SF-36 *	24 months	Age-matched Swiss population	M	36	87	-.23	.04
[illegible]eyen [24]	2007	Netherlands	Prospective Cohort	Minerva Medica	Medical-Surgical ICU	80+	83 (3)	60.00%[a]	49.60%	3.35 (2.26)	26.93 (27.11)	.26	EQ-5D	12 months	Retrospective patient or proxy ratings for one week before ICU	M	63	63	-.30	.03
Sacanella [23]	2011	Spain	Prospective Cohort	Critical Care	Medical ICU	65+	73.4 (5.5)	57.00%	48.70%	9.4 (10.20)		.27	EQ-5D	12 months	Retrospective patient or proxy ratings before feeling ill and requiring ICU	M	112	112	-.49	.02
Schroder [22]	2011	Denmark	Cohort (unspecified)	Danish Medical Bulletin	Mixed ICUs	75+		56.00%	53.85%				SF-36 *	12 months	Age-matched Danish population	L	36	229	-.03	.03
Sznajer [21]	2001	France	Prospective Cohort	Intensive Care Medicine	Mixed ICUs	65+ [b]		55.90%[a]					EQ-5D	6 months	ICU survivors younger than 65 years old	M	65	53	-.16	.03
Villa [19]	2016	Spain	Prospective Cohort	Journal of the American Geriatric Society	Medical-Surgical ICU	75+	80.8 (3.3)	50.00%	43.18%			.23	SF-36 *	12 months	Spanish population aged 75+	M	54	1363 [d]	-.15	.02

Table A1 Full study characteristics for all effect sizes included in the meta-analysis

[a] Reported for study level only
[b] Combined elderly groups
[c] Assumed N based on matched sample
[d] Retrieved from López-García, E., Banegas, J. R., Graciani, A. P. R., Gutiérrez-Fisac, J. L., Alonso, J., & Rodríguez-Artalejo, F. (2003). Population-based reference values for the Spanish version of the SF-36 Health Survey in the elderly. Medicina clinica, 120(15), 568-573; a follow-up to the previous study, which was unavailable
[e] Unless specified, we do not report data where it is not representative of at least 66.67% of the included sample
[f] Abbreviations: Avg. Age (average age); ICU LoS (average length of stay in intensive care; days); HLoS (average length of stay in hospital; days); SD (standard deviation; sometimes estimated- see methods)

For peer review only

NOTE: If studies are reported in duplicate, for the second row, assume blank cells are the same value as the row above, unless otherwise specified.

1.2 Qualitative Only Studies

First Author	Year	Country	Study Design	Journal	Setting	Min Age	Participant No.	Avg. Age (SD)	% Male	ICU LoS (SD)	HLoS (SD)	Severity	Ineligible Measure	Follow up	Comparison
Cuthbertson	2010	Scotland	Prospective Cohort	Critical Care	Medical-Surgical ICU	65+	116						SF-36 (MCS/PCS only)	12 months (paper reports up to 60 months)	ICU survivors younger than 65 years old AND retrospective ratings for a period before ICU
Garrouste-Orgeas	2006	France	Prospective Cohort	Intensive Care Medicine	Medical ICU	80+	28	84 (3.92)		12.6 (15.5)		.28	Nottingham Health Profile (NHP)	12 months	Age and sex-matched French population controls
Kleinpell	2002	USA	Retrospective Cohort	Research in Nursing and Health	Mixed ICUs	66+	128		42.00%	4.2 (6.17)	10.28 (9.63)	.18	Quality of Life Index (QLI)	4-6 months	ICU survivors aged between 45 and 64 years old
Tabah	2010	France	Prospective Cohort	Critical Care	Medical-Surgical ICU	80+	23	84 (3)	73.90%	5.72 (4.74)	18.08 (15.01)	.23	WHO-QOL-BREF	16 months	Age and sex-matched French population controls

Table A2 Full study characteristics of all records that were only included in the qualitative synthesis
[a] Reported for study level only
[b] Abbreviations: Avg. Age (average age); ICU LoS (average length of stay in intensive care; days); HLoS (average length of stay in hospital; days), SD (standard deviation; sometimes estimated- see methods)
[c] Unless specified, we do not report data where it is not representative of at least 66.67% of the included sample.

For peer review only

2. SENSITIVITY ANALYSES FOR INFLUENTIAL CASES

2.1 Overview of Outliers: Meta-Analysis

Comparison	k	First Author	Cook's Distance (Critical d)	Leave out Effect Size	Leave out P value	I^2 Change	Effect Size Change
Community	11	Pavoni	.97 (.36)	-1.97	.27	-12%	+1.74
Community	10	Honselmann	.56 (.40)	-.13	.10	-21%	+.08

Table A3 A summary of cases that fit our criteria as potentially influential

[a] Excluded cases are highlighted in red

First Author	Year	Country	Study Design	Journal	Setting	Min Age	Participant No.	Avg. Age (SD)	% Male	ICU LoS (SD)	HLoS (SD)	Severity	Mortality	Follow up	Comparison
Pavoni	2012	Italy	Prospective Cohort	Archives of Gerontology and Geriatrics	Mixed ICUs	80+	143	86.51 [a] (1.81)	26.74% [a]	5.27 [a] (5.80)	14.20 [a] (8.96)	.20 [a]	50% [a]	12 months	Age-matched Italian retirement community population

Table A4 Study characteristics of the lone study excluded as an outlier

[a] Reported for study level only

[b] Abbreviations: Avg. Age (average age); ICU LoS (average length of stay in intensive care; days); HLoS (average length of stay in hospital; days), SD (standard deviation; sometimes estimated- see methods)

3. QUALITATIVE SYNTHESIS

3.1 Qualitative analysis procedure

Scale	Mental Health Subscale(s)	Physcial Health Subscale(s)	Additional Notes
EQ-5D	Anxiety/Depression	Mobility, Self-Care, Usual Activities, Pain/Discomfort	Raw scores scaled between 1-3
SF-36	Social Functioning, Role Emotional, Mental Health, Vitality	Physical Functioning, Bodily Pain, General Health, Role Physical	
NHP	Sleep, Emotional Reaction, Social Isolation	Pain, Energy, Physical Mobility	Reverse scoring
WHO-QOL-BREF	Psychological Health, Social Relationships	Overall perception of Health, Physical Health, Environment	
QLI	Socio-economic, Family, Psychological/Spiritual	Health and Functioning	Raw scores scaled between 0-30

Table A5 Subscales used to estimate mental and physical health QoL within the qualitative synthesis

For peer review only

4. SENSITIVITY ANALYSES FOR OBSERVED EFFECTS

4.1 Forest Plots

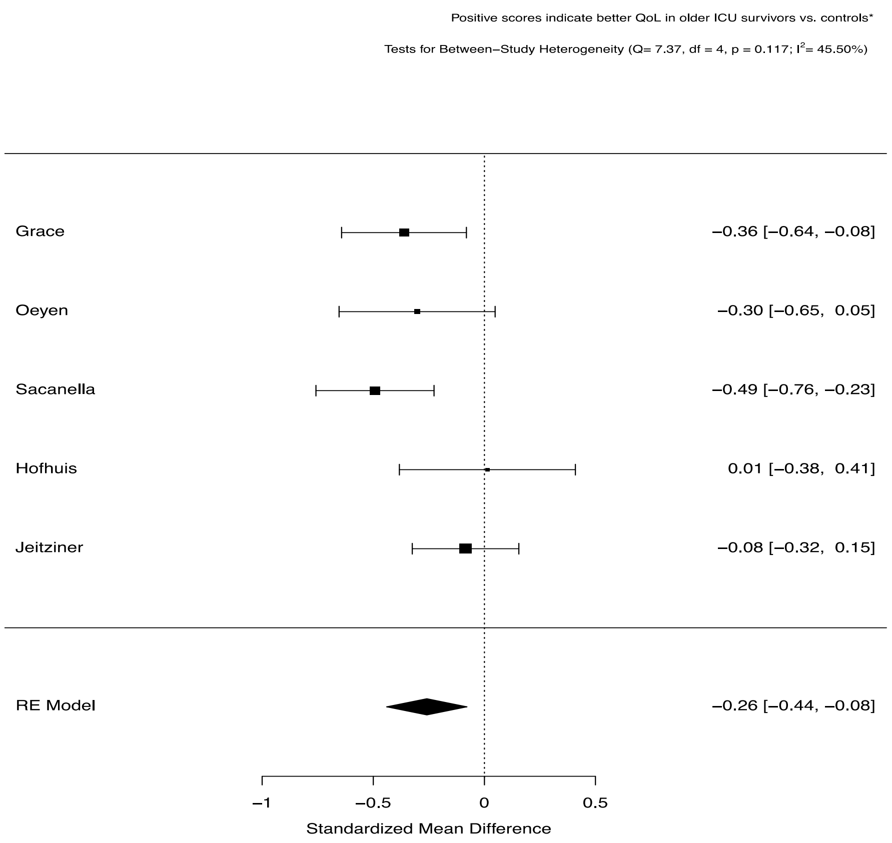

Fig. A1 Forest plot of differences in EQ-5D composite scores in elderly survivors, comparing pre-ICU and post-ICU scores

For peer review only - http://bmjopen.bmj.com/site/about/guidelines.xhtml

ICU Systematic Review: Appendix

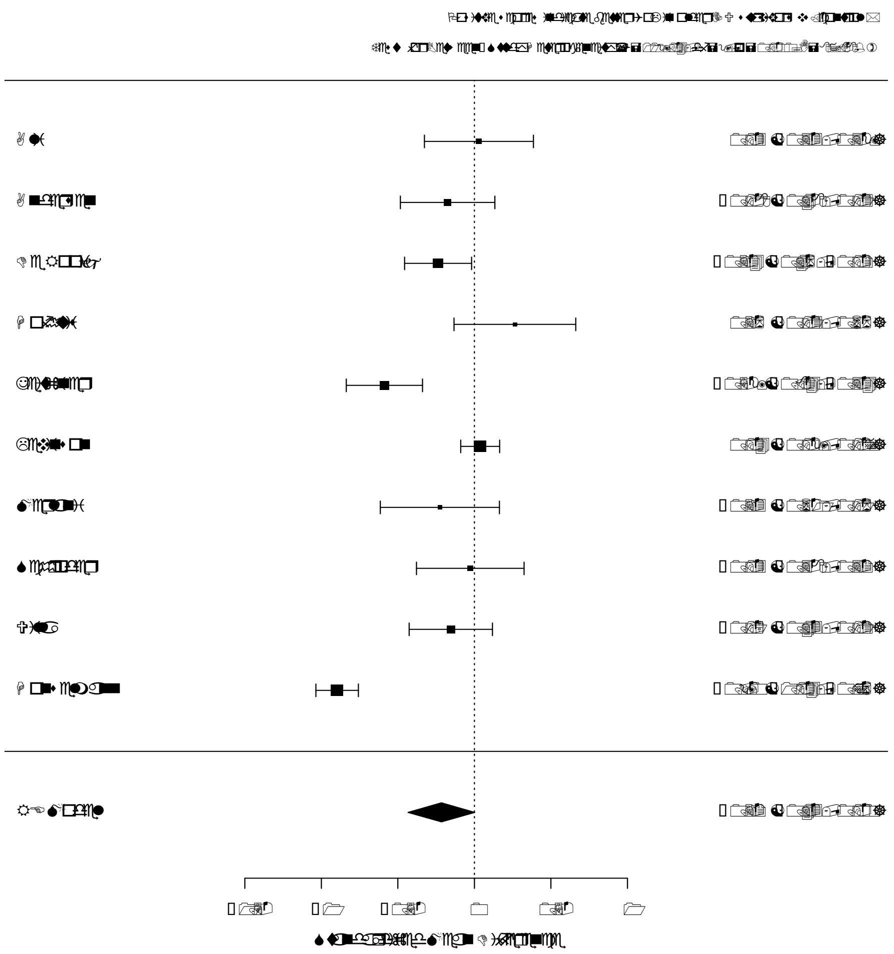

Fig. A2 Forest plot of differences in EQ-5D composite scores, comparing elderly ICU survivors at follow-up and age-matched community controls

For peer review only - http://bmjopen.bmj.com/site/about/guidelines.xhtml

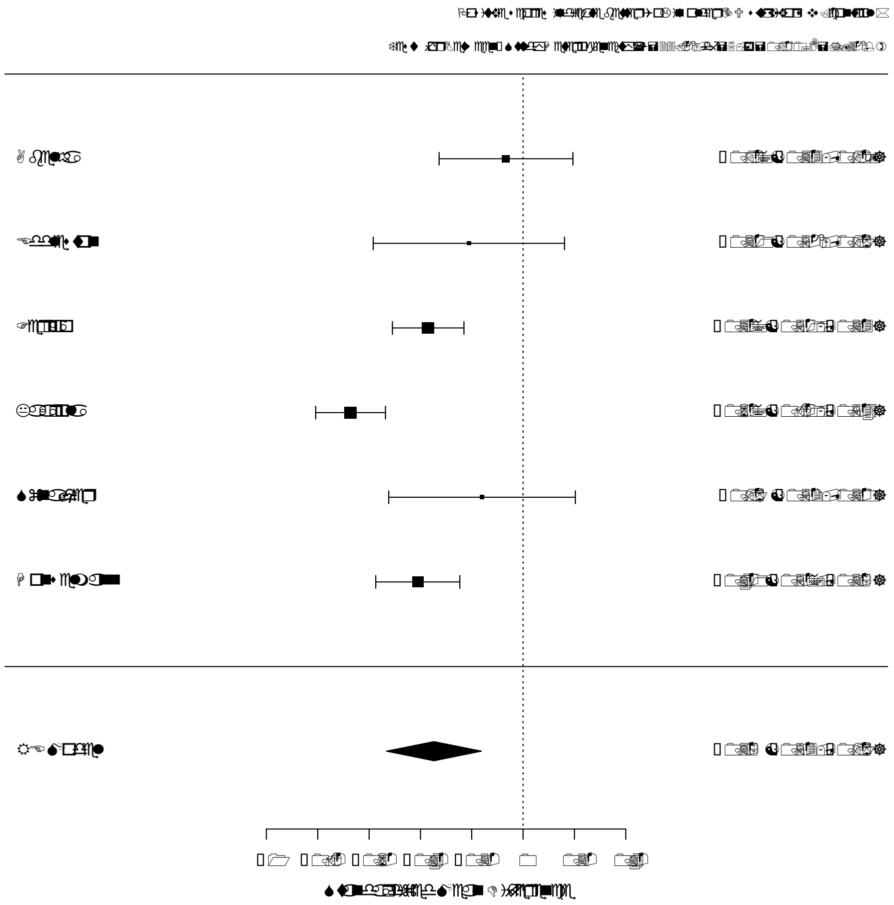

eview only

Fig. A3 Forest plot of differences in EQ-5D composite scores at follow-up, comparing elderly ICU survivors (aged 65+) and younger ICU survivors (aged under 65), both at follow-up

For peer review only - http://bmjopen.bmj.com/site/about/guidelines.xhtml

4.2 Funnel Plots

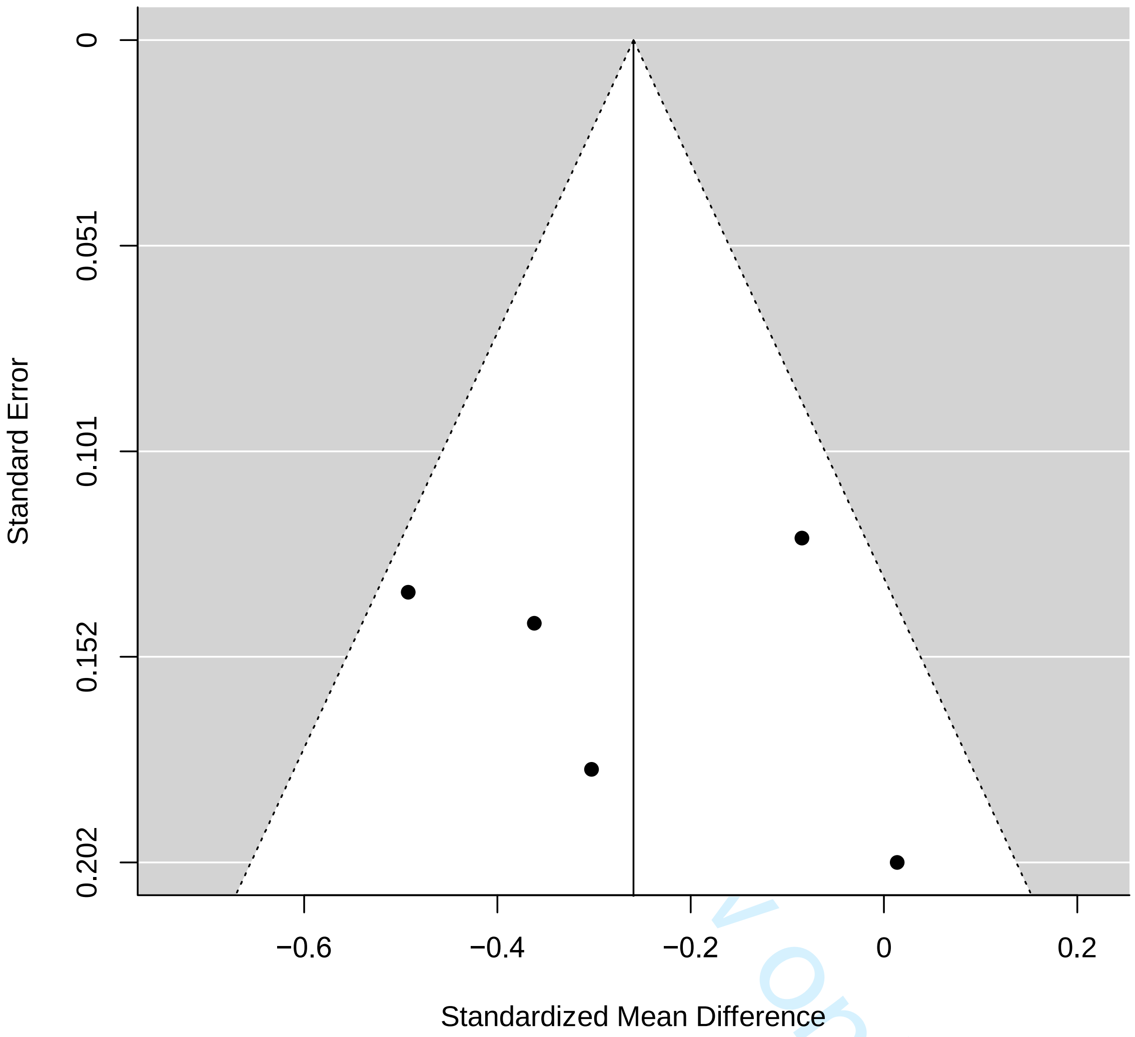

Fig. A4 Funnel plot of studies that investigated differences in EQ-5D composite scores in elderly survivors, comparing pre-ICU and post-ICU scores

For peer review only - http://bmjopen.bmj.com/site/about/guidelines.xhtml

Appendix: Disparity or Discrimination?
A systematic review of socio-demographic associations of insight

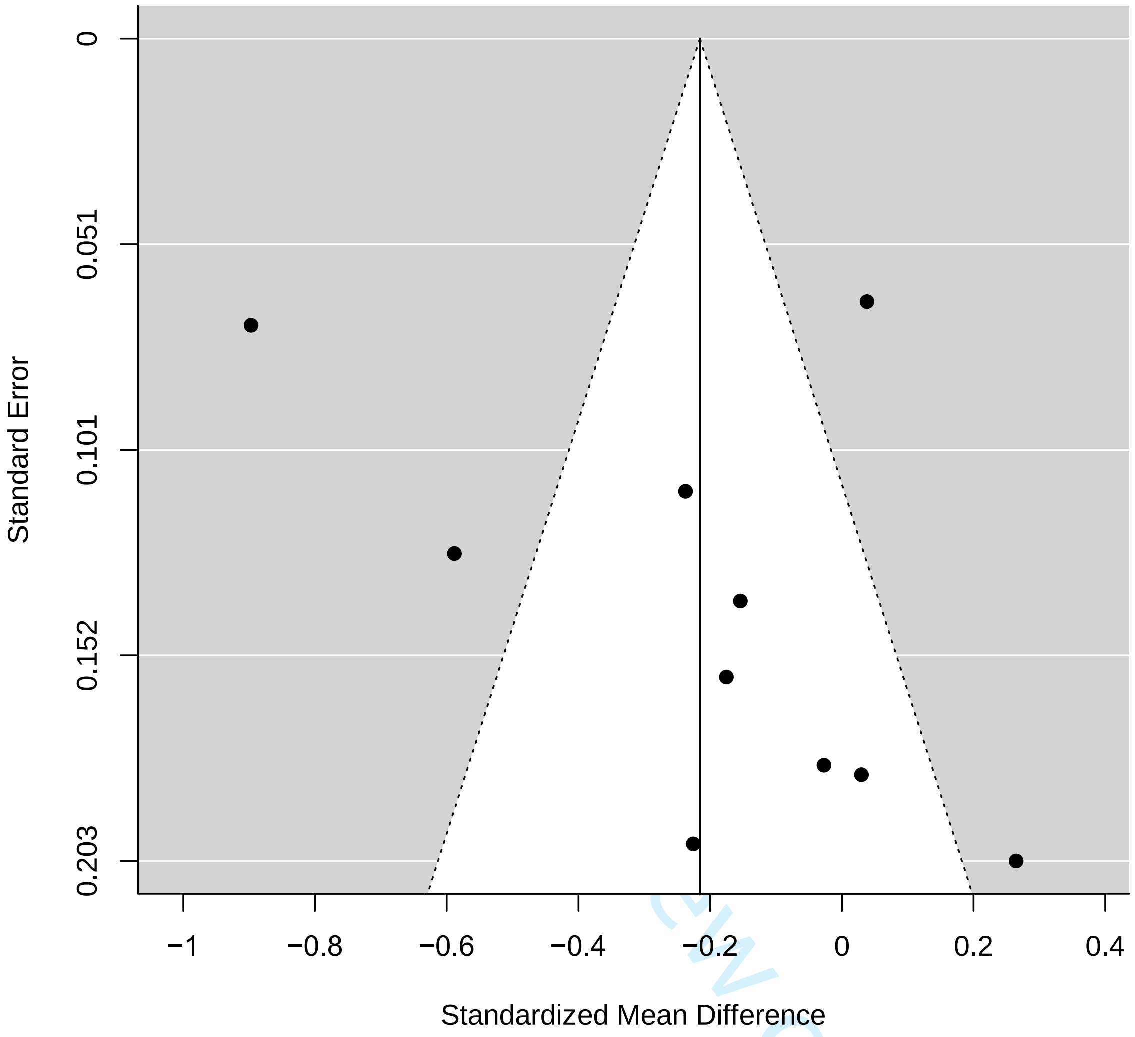

Fig. A5 Funnel plot of studies that compared EQ-5D scores in elderly ICU survivors at follow-up and age-matched community controls

For peer review only - http://bmjopen.bmj.com/site/about/guidelines.xhtml

Appendix: Disparity or Discrimination?
A systematic review of socio-demographic associations of insight

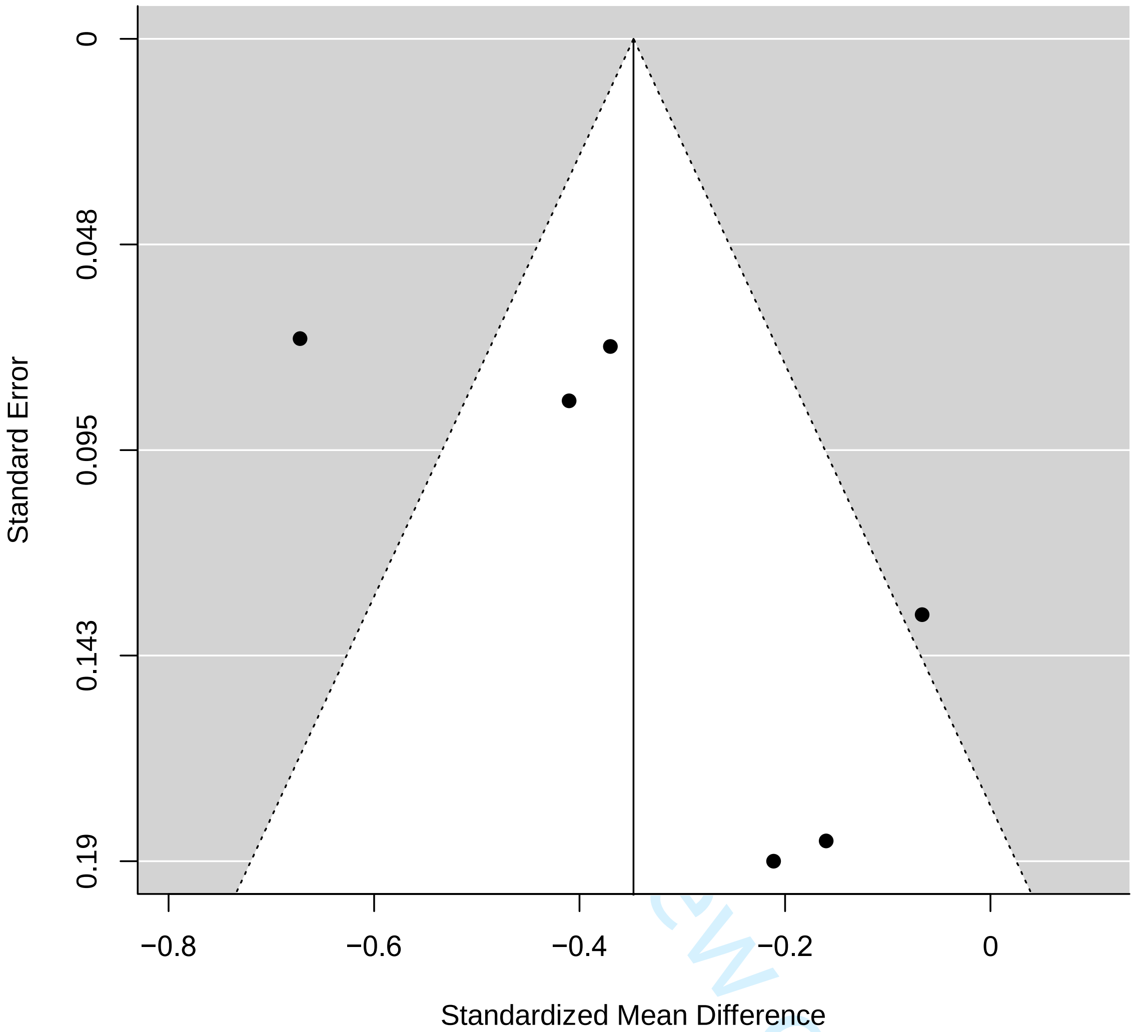

Fig. A6 Funnel plot of studies that compared EQ-5D scores in elderly ICU survivors (aged 65+) and younger ICU survivors (aged under 65), both at follow-up

For peer review only - http://bmjopen.bmj.com/site/about/guidelines.xhtml

4.3 Cook's Distance Plots

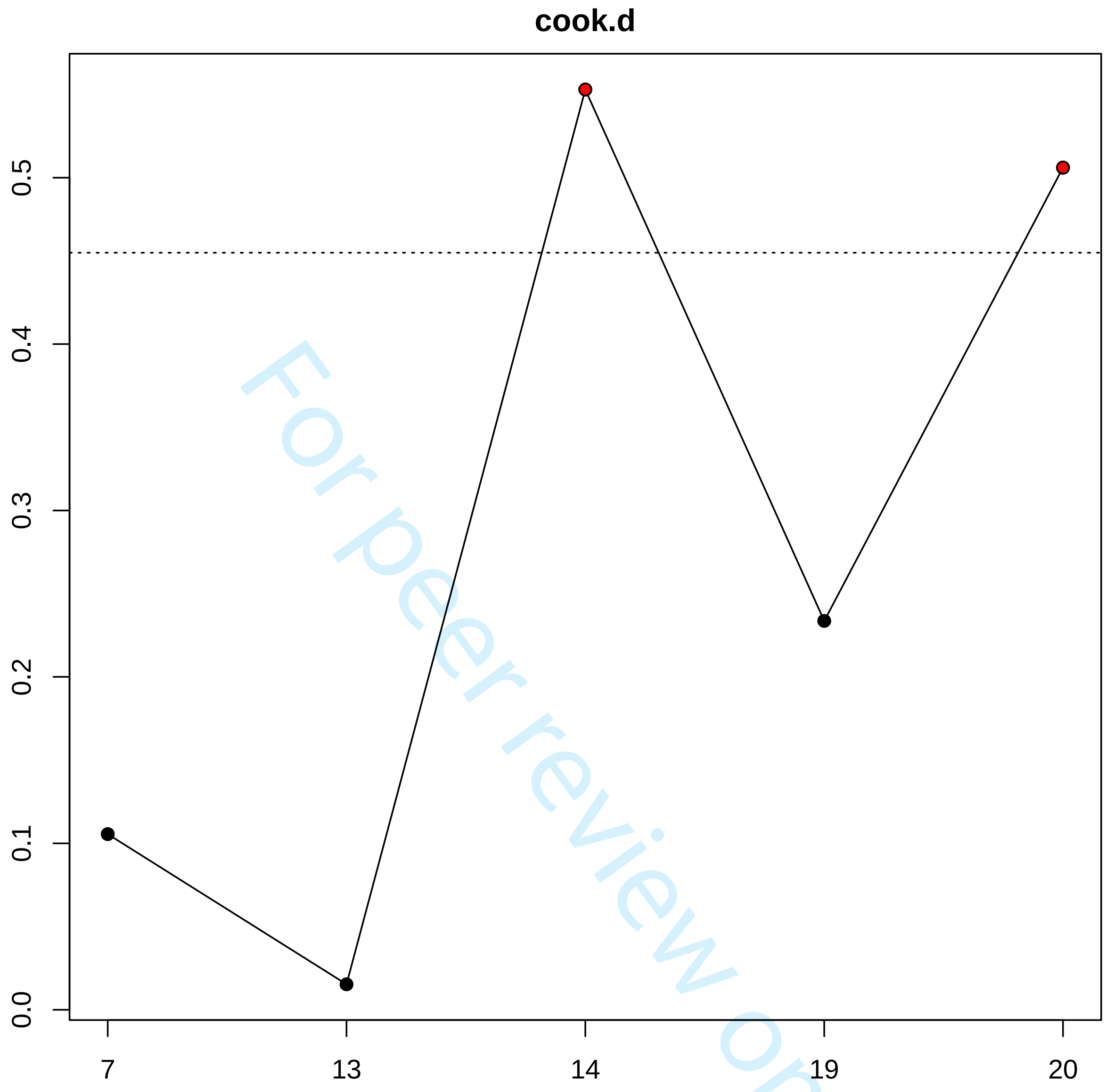

Fig. A7 Cook's distance plot of studies that investigated differences in EQ-5D composite scores in elderly survivors, comparing pre-ICU and post-ICU scores

For peer review only

For peer review only - http://bmjopen.bmj.com/site/about/guidelines.xhtml

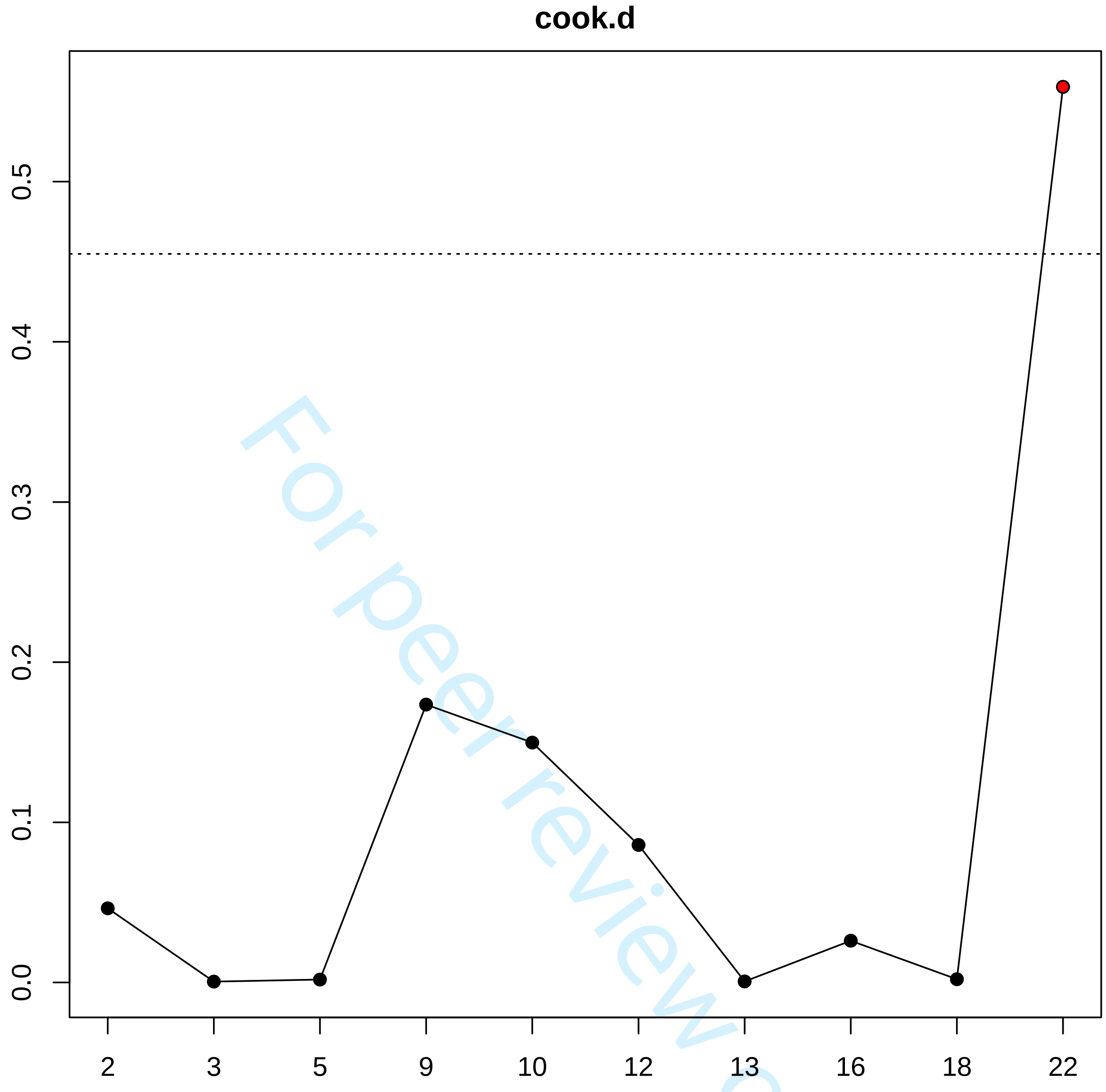

Fig. A8 Cook's distance plot of studies that compared EQ-5D scores in elderly ICU survivors at follow-up and age-matched community controls

For peer review only

Appendix: Disparity or Discrimination?
A systematic review of socio-demographic associations of insight

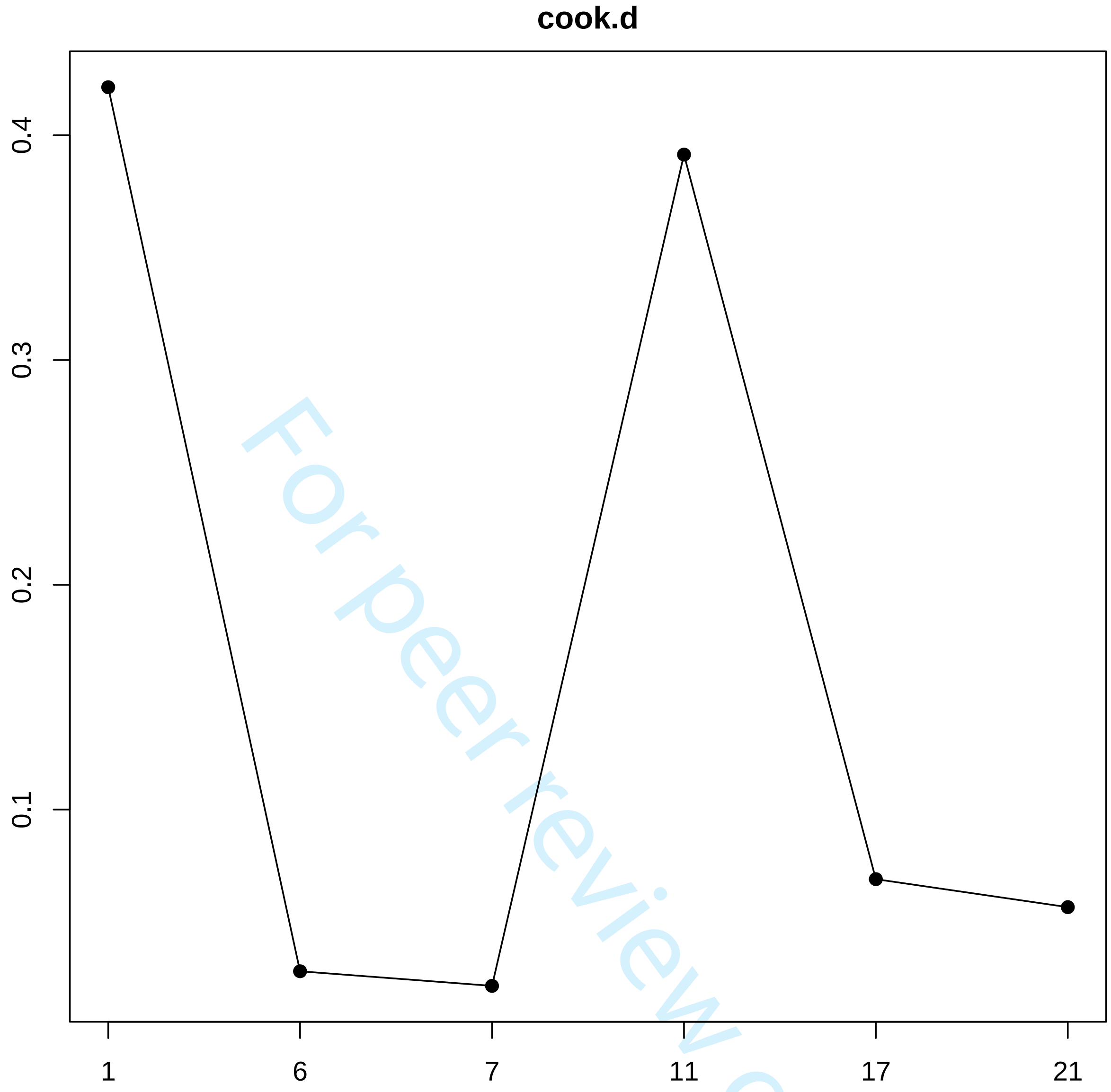

Fig. A9 Cook's distance plot of studies that compared EQ-5D scores in elderly ICU survivors (aged 65+) and younger ICU survivors (aged under 65), both at follow-up

For peer review only

Appendix: Disparity or Discrimination?
A systematic review of socio-demographic associations of insight

5. REVIEW PROTOCOL

5.1 ICU Review Protocol

Included	Excluded
**Design**	
Case note analyses (longitudinal)<br>Case control<br>Retrospective cohort<br>Prospective cohort<br>Unpublished dissertations of the above	Qualitative only studies<br>Systematic review or meta-analysis (categorise in separate folder)<br>Narrative review<br>Non-English language (if translation can't be found)<br>Commentaries<br>Case studies<br>Small N samples (<20 eligible participants)<br>Conference abstracts<br>Brief reports<br>Books
**Population**	
Patients aged 60+ who have undergone ICU<br>Medical, Surgical or Mixed ICU settings	<20 eligible patients aged 60+<br>Veteran, trauma or emergency care setting<br>Non-OECD country<br>Non-human participants<br>Palliative care<br>Non-ICU patients
**Focus**	
Patients aged 60+ who have undergone ICU	Neurological ICU patients only<br>Cardiosurgical ICU patients only
Follow up of at least 3 months	No follow up/Follow up less than three months
At least one of the following comparison groups:<br>• Age-matched community controls<br>• Scores taken before ICU<br>• Younger ICU patients	No comparison group
QoL at follow up measured by patients (carers may help but cannot do assessment on their own)	QoL at follow up all measured by proxy (ie. doctors or carers)
**Data/Outcomes**	
Validated QoL measure (EQ-5D, SF-36, NHP, WHOQOLBREF, QLI or variants of these)	Non-validated QoL measure only (eg. a simple question of whether QoL improved)
QoL summary score reported in paper for both groups, or:<br>• Subscores can be used to calculate summary scores<br>• Study references data for age-matched control that is fully reported elsewhere	No eligible data on QoL (or insufficient data to calculate summary scores)<br>QoL not reported for both groups (regression analyses do not count)

For peer review only - http://bmjopen.bmj.com/site/about/guidelines.xhtml

Appendix: Disparity or Discrimination?
A systematic review of socio-demographic associations of insight

6. REVIEW SEARCH TERMS

6.1 MEDLINE

(("intensive care"[title/abstract] OR "critical care"[title/abstract] OR "critical illness"[title/abstract] OR "Respiratory Distress Syndrome"[title/abstract] OR "Sepsis"[title/abstract] OR intensive care[MeSH Terms] OR critical care[MeSH Terms] OR "critical illness"[MeSH Terms] OR "Sepsis"[MeSH Terms]))

AND (("elderly"[title/abstract] OR "older adult*"[title/abstract] OR "geriatr*"[title/abstract] OR "dement*"[title/abstract] OR "Alzheimer*"[title/abstract] OR "parkinson's disease"[title/abstract] OR elderly [MeSH Terms] OR older adult*[MeSH Terms] OR geriatr*[MeSH Terms] OR dement*[MeSH Terms] OR septugenaria*[All Fields] OR octogenaria*[All Fields] OR nonagenaria*[All Fields] OR "over 5*"[title/abstract] OR "over 6*"[title/abstract] OR "over 7*"[title/abstract] OR "over 8*"[title/abstract] OR "over 9*"[title/abstract] OR "over 5*"[title/abstract] OR "over 6*"[title/abstract] OR "over 7*"[title/abstract] OR "over 8*"[title/abstract] OR "over 9*"[title/abstract]))

AND (("quality of life"[title/abstract] OR "EuroQol*"[All Fields] OR "Nottingham Health Profile"[All Fields] OR "NHP*"[All Fields] OR "SF-36"[All Fields] OR "RAND-36*"[All Fields]))

Filters: English Language, Humans, 01/01/2000 to 23/04/2020

6.2 Cochrane Database for Systematic Reviews & Cochrane Controlled Register of Trials (CENTRAL)

#1 ("intensive care" OR "critical care" OR "critical illness" OR "Respiratory Distress Syndrome" OR "Sepsis"):ti,ab,kw
#2 ("elderly" OR "older adult*" OR "geriatr*" OR "dement*" OR "Alzheimer*" OR "parkinson's disease"):ti,ab,kw
#3 (critical care OR critical illness OR Sepsis)
#4 (Aged OR geriatrics OR dementia)
#5 ("quality of life")
#6 ("EuroQol" OR "Nottingham Health Profile" OR "NHP" OR "SF-36" OR "RAND-36")
#7 MeSH descriptor: [Aged]
#8 MeSH descriptor: [Geriatrics]
#9 MeSH descriptor: [Dementia]
#10 MeSH descriptor: [Critical Care]
#11 MeSH descriptor: [Critical Illness]
#12 MeSH descriptor: [Sepsis]
#13 #1 OR #3 OR #10 OR #11 OR #12
#14 #2 OR #4 OR #7 OR #8 OR #9
#15 #5 AND #6
#16 #13 AND #14 AND #15= 124 (78 reviews, 36 trials).

6.3 Web of Science

Indexes = SCI-EXPANDED, SSCI, CPCI-S, CPCI-SHH, ESCI. **LANGUAGE** = English, **DOCUMENT TYPES** = (Article OR Abstract of Published Item), Timespan = All years (2000-2020)

#1 ALL=("intensive care" OR "critical care" OR "critical illness" OR "Respiratory Distress Syndrome" OR "Sepsis" OR "ICU")
#2 ALL=("elderly" OR "older adult*" OR "geriatr*" OR "dement*" OR "Alzheimer*" OR "parkinson's disease")
#3 ALL= ("quality of life" OR "EuroQol" OR "Nottingham Health Profile" OR "NHP" OR "SF-36" OR "RAND-36")
#4 #1 AND #2 AND #3
#5 #4 AND **LANGUAGE**: (English) AND **DOCUMENT TYPES**: (Article OR Abstract of Published Item) AND **Timespan**= 2000-2020

For peer review only - http://bmjopen.bmj.com/site/about/guidelines.xhtml

Appendix: Disparity or Discrimination?
A systematic review of socio-demographic associations of insight

6.4 EMBASE (& EMBASE Classic)

Dates: 2000-2020, Limits: Human participants only, English language, Articles only

#1 All Field: "intensive care" or "critical care" or "critical illness" or "Respiratory Distress Syndrome" or Sepsis or "ICU"
#2 Text Word: elderly or "older adult*" or "geriatr*" or "dement*" or "Alzheimer*" or "parkinson*"
#3 All Field: "quality of life" or EuroQol or Nottingham Health Profile or NHP or SF-36 OR RAND-36

6.5 CINAHL

Limits: English language only, Human participants, All adult, Peer-reviewed, Jan 2000 – April 2020

#1 TX: "intensive care" or "critical care" or "critical illness" or "Respiratory Distress Syndrome" or Sepsis or "ICU"
#2: SU: "Intensive Care Units" or "Intensive Care Units or Neonatal" or "Critical Care Nursing" or "Respiratory Distress Syndrome" or Acute or "Neonatal Intensive Care Nursing" or "Critical Care or Critical Path" or "Canadian Association of Critical Care Nurses" or "British Association of Critical Care Nurses" or "ventilator patients"
#3: TX: elderly or "older adult*" or "geriatr*" or "dement*" or "Alzheimer*" or "parkinson*"
#4: SU: "Older Adult Care (Saba CCC)" or "Frail Elderly" or "elderly patients" or "ventilator patients"
#5: TX: "quality of life" or EuroQol or “Nottingham Health Profile” or NHP or SF-36 OR RAND-36
#6: (S1 OR S2) AND (S3 OR S4) AND S5

6.6 PsycINFO

Limits: Date filter (2000-2020), English language, Human participants, Peer Reviewed Journal

#1 All Fields: "intensive care" or "critical care" or "critical illness" or "Respiratory Distress Syndrome" or Sepsis or "ICU"
#2 Text Word: elderly or "older adult*" or "geriatr*" or "dement*" or "Alzheimer*" or "parkinson*"
#3 All Fields: "quality of life" or EuroQol or Nottingham Health Profile or NHP or SF-36 OR RAND-36

For peer review only - http://bmjopen.bmj.com/site/about/guidelines.xhtml

PRISMA 2009 Checklist

Section/topic	#	Checklist item	Reported on page #
**TITLE**			
Title	1	Identify the report as a systematic review, meta-analysis, or both.	1
**ABSTRACT**			
Structured summary	2	Provide a structured summary including, as applicable: background; objectives; data sources; study eligibility criteria, participants, and interventions; study appraisal and synthesis methods; results; limitations; conclusions and implications of key findings; systematic review registration number.	1
**INTRODUCTION**			
Rationale	3	Describe the rationale for the review in the context of what is already known.	3
Objectives	4	Provide an explicit statement of questions being addressed with reference to participants, interventions, comparisons, outcomes, and study design (PICOS).	3
**METHODS**			
Protocol and registration	5	Indicate if a review protocol exists, if and where it can be accessed (e.g., Web address), and, if available, provide registration information including registration number.	1 & 4
Eligibility criteria	6	Specify study characteristics (e.g., PICOS, length of follow-up) and report characteristics (e.g., years considered, language, publication status) used as criteria for eligibility, giving rationale.	4, 8 & Appendix
Information sources	7	Describe all information sources (e.g., databases with dates of coverage, contact with study authors to identify additional studies) in the search and date last searched.	4 & Appendix
Search	8	Present full electronic search strategy for at least one database, including any limits used, such that it could be repeated.	4 & Appendix
Study selection	9	State the process for selecting studies (i.e., screening, eligibility, included in systematic review, and, if applicable, included in the meta-analysis).	4-6 & Appendix
Data collection process	10	Describe method of data extraction from reports (e.g., piloted forms, independently, in duplicate) and any processes for obtaining and confirming data from investigators.	4-5 & Appendix
Data items	11	List and define all variables for which data were sought (e.g., PICOS, funding sources) and any assumptions and simplifications made.	4 & Appendix
Risk of bias in individual studies	12	Describe methods used for assessing risk of bias of individual studies (including specification of whether this was done at the study or outcome level), and how this information is to be used in any data synthesis.	5, 9, 10 and Appendix
Summary measures	13	State the principal summary measures (e.g., risk ratio, difference in means).	5

For peer review only - http://bmjopen.bmj.com/site/about/guidelines.xhtml

PRISMA 2009 Checklist

Synthesis of results	14	Describe the methods of handling data and combining results of studies, if done, including measures of consistency (e.g., I^2) for each meta-analysis.	5

Section/topic	#	Checklist item	Reported on page #
Risk of bias across studies	15	Specify any assessment of risk of bias that may affect the cumulative evidence (e.g., publication bias, selective reporting within studies).	5, 9, 10 and Appendix
Additional analyses	16	Describe methods of additional analyses (e.g., sensitivity or subgroup analyses, meta-regression), if done, indicating which were pre-specified.	5
**RESULTS**			
Study selection	17	Give numbers of studies screened, assessed for eligibility, and included in the review, with reasons for exclusions at each stage, ideally with a flow diagram.	6
Study characteristics	18	For each study, present characteristics for which data were extracted (e.g., study size, PICOS, follow-up period) and provide the citations.	8 & Appendix
Risk of bias within studies	19	Present data on risk of bias of each study and, if available, any outcome level assessment (see item 12).	8-10 & Appendix
Results of individual studies	20	For all outcomes considered (benefits or harms), present, for each study: (a) simple summary data for each intervention group (b) effect estimates and confidence intervals, ideally with a forest plot.	9 & Appendix
Synthesis of results	21	Present results of each meta-analysis done, including confidence intervals and measures of consistency.	9 & Appendix
Risk of bias across studies	22	Present results of any assessment of risk of bias across studies (see Item 15).	9-10 & Appendix
Additional analysis	23	Give results of additional analyses, if done (e.g., sensitivity or subgroup analyses, meta-regression [see Item 16]).	9-10 & Appendix
**DISCUSSION**			
Summary of evidence	24	Summarize the main findings including the strength of evidence for each main outcome; consider their relevance to key groups (e.g., healthcare providers, users, and policy makers).	12
Limitations	25	Discuss limitations at study and outcome level (e.g., risk of bias), and at review-level (e.g., incomplete retrieval of identified research, reporting bias).	13-14
Conclusions	26	Provide a general interpretation of the results in the context of other evidence, and implications for future research.	13, 15

For peer review only

For peer review only - http://bmjopen.bmj.com/site/about/guidelines.xhtml

PRISMA 2009 Checklist

FUNDING			
Funding	27	Describe sources of funding for the systematic review and other support (e.g., supply of data); role of funders for the systematic review.	15

From: Moher D, Liberati A, Tetzlaff J, Altman DG, The PRISMA Group (2009). Preferred Reporting Items for Systematic Reviews and Meta-Analyses: The PRISMA Statement. PLoS Med 6(7): e1000097. doi:10.1371/journal.pmed1000097

For more information, visit: **www.prisma-statement.org**.

For peer review only
